# Pathway-Masked Kolmogorov–Arnold Networks Yield an Interpretable Transcriptomic Score Associated with Colorectal Cancer Progression Independently of Clinical Stage

**DOI:** 10.3390/life16101743

**Published:** 2026-10-17

**Authors:** Khaled S. Allemailem, Hajed Obaid Alharbi, Faris Alrumaihi, Ahmad Almatroudi

**Affiliations:** Department of Medical Laboratories, College of Applied Medical Sciences, Qassim University, Buraydah 51452, Saudi Arabia; hajed.alharbi@qu.edu.sa (H.O.A.); f_alrumaihi@qu.edu.sa (F.A.); aamtrody@qu.edu.sa (A.A.)

**Keywords:** colorectal cancer, prognostic biomarker, gene expression signature, Kolmogorov–Arnold network, interpretable machine learning, pathway-informed deep learning, progression-free interval, external validation, precision oncology, gastrointestinal cancer

## Abstract

Background: Prognostic gene expression signatures in colorectal cancer are usually reported by standalone discrimination, a standard that random gene sets can meet, and are rarely tested for what they add to routine clinical variables. Methods: TCGA-COAD and TCGA-READ were pooled into a single colorectal cohort (596 patients, 153 progression-free events). PathKAN places learnable univariate spline functions on a sparse gene-to-pathway topology drawn from MSigDB and BioGRID, reducing 20,681,472 dense weights to 127,985 edges, and is fitted under a Cox partial-likelihood objective. Performance was assessed by nested, repeated stratified cross-validation with dependence-corrected inference against clinical, elastic-net Cox and random survival forest models, and the frozen network was applied to four independent cohorts (1012 patients, 306 events) on two array platforms. Results: The out-of-fold score was associated with progression-free interval independently of stage, age, sex and tumour site (adjusted HR 1.25 per SD, 95% CI 1.06–1.47; likelihood-ratio *p* = 0.008), but raised concordance only from 0.699 to 0.711, a gain whose 95% confidence interval included zero. The frozen model remained independently prognostic in the two larger treatment-naive validation cohorts (*p* = 0.003 and *p* = 0.008) but not in the two smaller cohorts, and was not prognostic in oesophageal or lung adenocarcinoma. Individual learned edge functions were often non-monotonic but were not reproducible across refits, whereas gene-level partial-dependence curves were stable and largely monotone, and only some of the top-ranked genes recurred consistently. Of the 50 highest-ranked genes, 42 were differentially expressed between tumour and adjacent normal tissue. Conclusions: A pathway-masked Kolmogorov–Arnold survival model yields an interpretable transcriptomic score associated with colorectal cancer progression independently of clinical stage. It improves model fit over clinical variables, but its gain in discrimination is small and not statistically established; its transportability depends partly on outcome-blind, cohort-level standardisation of the inputs, and its clinical utility remains to be established.

## Simple Summary

Doctors decide how aggressively to treat colorectal cancer largely on the basis of tumour stage, yet patients with the same stage often have very different outcomes. Molecular tests have been proposed to fill that gap, but most report how well they predict outcomes on their own rather than whether they add anything to the information a clinician already has. This study builds a model that reads tumour gene activity through known biological pathways, and that shows how each gene-to-pathway connection transforms gene activity on its way to the risk estimate. In 596 patients, the resulting score was associated with disease progression after accounting for stage, age, sex and tumour location, although it improved the ranking of patients by risk only slightly. The same association was found in two of four further groups of patients measured on different technology, the two larger ones, and not in patients with oesophageal or lung cancer. In those groups, each gene measurement was first standardised against the rest of the group, so the score cannot yet be calculated for a single new patient until a fixed reference for that step is established. Most of the genes the model relied on are expressed differently in colorectal tumours than in normal bowel tissue. The score is a candidate for further development rather than a test ready for clinical use.

## 1. Introduction

Colorectal cancer accounts for roughly one in ten cancer diagnoses and one in ten cancer deaths worldwide, placing it third for incidence and second for mortality [1]. Incidence in adults under fifty has been rising for three decades even as overall rates fall, so the disease is reaching a younger population than the screening programmes were designed around [2]. Most patients present with localised disease and are treated with surgery, sometimes followed by adjuvant chemotherapy, and roughly a third will nonetheless relapse [3]. That decision, whether to give adjuvant treatment after resection, still rests largely on anatomical stage together with a short list of pathological risk features, and current European guidance acknowledges how imperfectly those features separate the patients who benefit from those who do not [4]. Molecular markers have long been proposed to close the gap, and the field is now substantial [5]: transcriptional taxonomies divide colorectal tumours into consensus subtypes with distinct outcomes [6]; the integrated genomic characterisation of colon and rectal tumours established that the two sites are molecularly one disease once hypermutation is accounted for [7] and microsatellite instability entered routine practice as both a prognostic and a predictive marker [8]. Among tissue-based scores, the Immunoscore is the clearest example of a molecular measurement earning a place beside the TNM classification through international validation [9].

Against that, the number of published expression signatures vastly exceeds the number in clinical use, and part of the explanation is how they are evaluated. Venet and colleagues demonstrated that most randomly chosen gene sets of sufficient size are significantly associated with outcome, because expression tracks proliferation and proliferation tracks stage [10]. A signature reported by its own concordance may therefore be restating stage in molecular language. Reporting standards for prediction models ask instead for the incremental contribution over the variables already available to the clinician [11], which is a considerably harder test and a more useful one. Machine learning has been applied to this problem repeatedly, and the recurring difficulty is interpretability rather than accuracy. Kolmogorov–Arnold networks offer one route: instead of fixed activation functions and scalar weights, each connection carries its own learnable univariate function [12], an idea whose theoretical basis has been revisited in the context of modern networks [13]. Applied to molecular data, this formulation has been combined with protein interaction graphs for pan-cancer classification [14]. Separately, constraining a network to known pathway structure produced a biologically informed model in prostate cancer whose internal units could be interrogated directly [15]. Deep survival models brought the Cox objective to neural networks [16,17] and to multi-omics integration [18], but retained densely connected, scalar-weighted layers. Careful benchmarking has since shown that well-tuned conventional baselines match these architectures on bulk expression survival tasks [19], which shifts the burden of proof: a new architecture must justify itself by what it reveals, not only by where it ranks.

The methodological gap is specific. Conventional Cox regression [20] and its penalised extensions [21] give each gene one coefficient, and so assume that the log-hazard changes linearly across a gene’s whole expression range. Dense deep-survival networks [16,17] and multi-omics survival models [18] relax that assumption, but distribute each gene’s effect across many scalar weights that cannot be read individually. Pathway-informed networks make internal units interpretable as pathways [15], and Kolmogorov–Arnold layers have been placed on molecular graphs [14], but these models were built for classification rather than time-to-event outcomes. PathKAN combines the sparse prior-knowledge topology of pathway-informed networks with a learnable univariate function on every retained connection, fitted to a Cox objective, so that the transformation applied on each gene-to-pathway edge can be inspected directly. We evaluated it along three dimensions, each with a defined assessment. Incremental prognostic value: whether an out-of-fold score adds to stage, age, sex and tumour site, judged by likelihood-ratio tests, adjusted hazard ratios and the cross-validated change in concordance. External transportability: whether the network, frozen after fitting on sequencing data, remains associated with outcome in independent microarray cohorts, judged by concordance and covariate-adjusted Cox regression in each cohort. Disease relevance: whether the genes and pathways the model prioritises relate to colorectal cancer biology, judged by tumour-versus-normal expression, colorectal-annotated cancer genes under a connectivity-matched null, molecular subtype, and the behaviour of the score in non-colorectal cohorts.

## 2. Materials and Methods

### 2.1. Discovery Cohort and Endpoint

RNA sequencing and clinical records for TCGA-COAD and TCGA-READ were retrieved with TCGAbiolinks 2.39.0 [22] under R 4.6.0 (R Foundation for Statistical Computing, Vienna, Austria) on 30 April 2026, corresponding to GDC data release 45.0, and normalised with the DESeq2 1.51.7 variance-stabilising transformation [23]. Colon and rectal tumours were analysed together as a single colorectal cohort. That decision follows the TCGA integrated analysis of colorectal carcinoma [7], which found the two sites molecularly indistinguishable once hypermutation status is taken into account. Anatomical site was nevertheless carried through every model as a covariate and used to stratify all cross-validation splits, so the assumption is testable rather than assumed away. Because the two projects were variance-stabilised independently, the rectal block was quantile-aligned onto the colon reference distribution before the matrices were merged. Version suffixes were stripped from Ensembl identifiers and the 44 pseudoautosomal duplicates removed. Genes clearing the transformation floor in at least a quarter of samples were retained, leaving 39,589 of 60,616, and these were mapped to approved HGNC symbols [24]; where several identifiers were mapped to one symbol, the transcript with the highest mean expression was kept. Analysis was restricted to primary tumours, and patients contributing more than one aliquot were reduced to a single sample so that no individual could appear on both sides of a cross-validation split. The resulting discovery cohort contains 596 patients, 438 colonic and 158 rectal. The primary endpoint is the progression-free interval as curated in the TCGA Clinical Data Resource [25]. That resource recommends this endpoint specifically for colon and rectal adenocarcinoma: overall survival in these two projects is sparse in events and short in follow-up, and in a cohort of median age 68, a substantial share of deaths have nothing to do with the tumour. Progression-free interval yields 153 events, all with positive follow-up, and restricts those events to disease progression. Vital status records 124 deaths, but four have a recorded survival time of zero or none and cannot enter a time-to-event analysis, so overall survival provides 120 analysable events among 588 patients. Overall survival was retained as a secondary endpoint. AJCC stage was taken from the GDC clinical record and, for the 112 patients whose records were empty, from the curated stage in the TCGA Clinical Data Resource [25]; 20 patients remained unstaged and were assigned the median stage in the clinical models. A missing-stage indicator, stage as a categorical variable, and a complete-case analysis were examined as sensitivity analyses.

### 2.2. Independent Validation Cohorts

Four public colorectal series were obtained directly from the GEO FTP archive [26] as series matrices and annotated from the corresponding platform tables. Selection required primary colorectal tumours, curated follow-up in the deposited characteristics, and coverage of both colonic and rectal disease across more than one array platform. GSE39582 (*n* = 556, 187 events) is the French CIT series and serves as the principal validation set [27]; GSE17536 (*n* = 177, 73 events) provides an independent American series [28]; GSE33113 (*n* = 89, 18 events) is restricted to stage II disease [29]; and GSE87211 (*n* = 190, 28 events) contributes rectal tumours profiled on an Agilent platform [30]. Together, these supply 1012 patients and 306 events, twice the event count of the discovery cohort. Non-tumour samples and cases without usable follow-up were excluded, and probes were collapsed to HGNC symbols by taking the brightest probe set per gene. Every patient in GSE87211 received preoperative chemoradiation, so that series addresses prognosis after treatment rather than the treatment-naive question the model was trained on, and it is reported separately throughout.

### 2.3. Expression Representation

Training and validation share one representation. For each sample, genes were ranked by expression, and the ranks were converted to normal quantiles,

(1) $${\tilde{x}}_{gi}=\Phi^{-1}\left(\frac{r_{gi}-0.5}{p}\right)$$

where *r* is the rank of gene *g* within sample *i*, *p* is the number of genes, and *Φ* is the standard normal distribution function. Each gene was then centred and scaled; during cross-validation, the mean and standard deviation were estimated on the training partition of each fold and applied unchanged to its test partition. Only the ordering of genes within a patient survives this transform, which is what allows a model fitted on sequencing counts to be applied to two different microarray platforms. Fitting on variance-stabilised counts and predicting on ranks would impose a shift the network was never trained to absorb. Four preprocessing steps preceded cross-validation and were fitted on the pooled discovery matrix: the variance-stabilising transformation, quantile alignment of the rectal block, the expression filter and the choice of one transcript per gene symbol. None uses outcome or any other clinical variable. The first two are monotone within each sample and therefore leave unchanged the within-sample ranks that form the model input; the last two influence which genes are modelled, but no fitted parameter. In each external cohort, the rank transform was followed by per-gene centring and scaling estimated within that cohort, again without outcome data, while every network weight stayed fixed. Because this step uses the external cohort’s own expression distribution, transfer was also evaluated with centring and scaling fixed at the discovery-cohort values. Because the within-cohort step requires a group of samples, the external results describe the frozen network together with this cohort-level preprocessing, and they do not by themselves support scoring a single patient.

### 2.4. Prior-Knowledge Network

The gene-to-pathway incidence matrix was assembled from the MSigDB v2026.1 Hallmark, Reactome and KEGG-MEDICUS collections [31,32,33,34] and restricted to sets of 10 to 300 genes. The gene space was further narrowed to transcripts measured on the sequencing platform and on all four validation arrays. Without that intersection, the frozen model meets missing inputs at validation and must impute them, which quietly erodes transfer. The final input space holds 9749 genes and 1792 pathways connected by 76,925 edges, a density of 0.44 per cent, and gives complete gene coverage in every external series. Pathway-to-pathway adjacency was derived from the 469,270 human physical interactions in BioGRID 5.0.257 [35] that fall among masked genes. Raw interaction overlap links roughly 94 per cent of pathway pairs and therefore constrains nothing, so each pair was scored by shared interactions normalised for set size, and every pathway kept its fifteen strongest partners. That leaves 51,060 edges at 1.59 per cent density. The COSMIC Cancer Gene Census v103 [36,37] and OncoKB [38] gene lists were excluded from the model entirely. They are used only after fitting, to ask whether the prioritised genes recover established cancer biology, and holding them out is what makes that question meaningful.

### 2.5. PathKAN Architecture

#### 2.5.1. Masked Kolmogorov–Arnold Layers

A Cox model assigns each gene a scalar coefficient, so the most it can say is that higher expression carries more risk, in proportion. A conventional neural network can represent nonlinear effects, but spreads them across many scalar weights that cannot be read individually. PathKAN replaces those scalars with functions, following the Kolmogorov–Arnold network formulation [12,13]. Each surviving connection between input *i* and output *j* carries its own learnable univariate map,

(2) $$\varphi_{ij}\left(x\right)=b_{ij}\cdot SiLU\left(x\right)+\sum_{k=1}^{K}c_{ijk}B_{k}\left(x\right)$$

in which *B* denotes a cubic B-spline basis on a fixed grid of *K* knots, *b* is a scalar on a sigmoid-weighted linear unit that keeps the edge trainable where spline support is thin, and *c* are the spline coefficients. The output of a layer is the sum of these functions over the connections that prior knowledge sanctions,

(3) $$h_{j}=\sum_{i\in N\left(j\right)}g_{ij}\varphi_{ij}\left(x_{i}\right)$$

with N(*j*) representing the inputs linked to unit *j* by the mask and *g* a multiplicative gate on each edge. The mask is stored as an edge list rather than a dense matrix, so memory scales with the number of retained connections rather than with the product of the layer dimensions.

#### 2.5.2. Topology and Objective

Genes feed pathways, pathways feed the pathway-interaction layer, and a bias-free linear map returns a log-hazard. Layer normalisation and dropout [39] sit between the two masked layers. Two auxiliary linear heads predict microsatellite instability status and TCGA expression subtype from the shared pathway representation; they carry no weight at prediction time and exist to regularise that representation during fitting. Masking reduces the two layers from 20,681,472 dense weights to 127,985 edges, just over half a per cent of the unconstrained parameterisation. Risk is fitted by the Breslow approximation [40] to the negative Cox partial log-likelihood [20],

(4) $$L_{cox}=-\frac{1}{\left|D\right|}\sum_{i\in D}\left(\eta_{i}-log\sum_{k\in R\left(i\right)}exp\left(\eta_{k}\right)\right)$$

where *η* is the predicted log-hazard, *D* indexes patients with an observed event and *R*(*i*) is the risk set at that event time. The quantity minimised during training adds the auxiliary terms and an L1 penalty on the gates, which allows the network to switch off connections that carry nothing and leaves a readable subnetwork behind,

(5) $$L=L_{cox}+\lambda_{1}L_{msi}+\lambda_{2}L_{sub}+\lambda_{3}\frac{1}{E}\sum_{\left(i,j\right)\in E}\left|g_{ij}\right|$$

with *E* indicating the retained edge set. Batches containing no events return zero rather than an undefined gradient, which matters at a 26 per cent event rate.

#### 2.5.3. Efficient Spline Evaluation

Evaluating the Cox–de Boor recursion once per sample and per edge builds an intermediate tensor of several hundred million elements and dominated runtime in early profiling. The basis depends only on the input value, so it is computed once on a fixed 256-point grid; each forward pass contracts that basis with the spline coefficients into a per-edge lookup table and reads the table by linear interpolation. This is a numerical approximation to the B-spline, and we quantified it by re-evaluating every saved network with the exact recursion. Across all 127,985 edge functions of each of the ten cross-validation and five final networks, the largest absolute difference was 1.2 × 10^−5^, at most 0.06% of an edge function’s amplitude; the largest change in any predicted log-hazard was 1.5 × 10^−3^, the rank correlation between the two sets of predictions was at least 0.99999, and concordance changed by at most 0.0005 in any fold or cohort. A full-batch epoch fell from 13.8 s to 1.1 s on CPU and to 0.19 s on an NVIDIA RTX 4060 (NVIDIA Corporation, Santa Clara, CA, USA), which is what made repeated cross-validation and a hyperparameter search practical on a single workstation.

### 2.6. Training and Model Selection

Early stopping proved unusable here. A held-out fifth of a training fold carries roughly 25 events; its concordance estimate swings widely from epoch to epoch, and an early lucky reading was repeatedly being restored as the best model. Networks are therefore trained for a fixed epoch budget, and the weights of the final 30 epochs are averaged, a form of stochastic weight averaging [41]; the inner split is monitored but never selects the model. Optimisation used AdamW, which decouples weight decay from the gradient update [42], with a cosine-annealed learning rate and gradient-norm clipping at unity. Three independently seeded networks were averaged in each fold to damp initialisation variance. Learning rate, L1 weight, dropout, weight decay and epoch budget were chosen from 24 configurations by fully nested cross-validation [43]. Within each outer training partition, every configuration was scored by a three-fold cross-validation of that partition alone; the best configuration was then refit on the whole partition with three seeds and applied once to the outer test fold, which therefore took no part in tuning or fitting. The configuration chosen in each outer fold was recorded, and the nested estimates were compared with a non-nested variant in which the search was run once on the whole discovery cohort. The network used for interpretation and external validation was fitted to the whole discovery cohort with the configuration selected by the same search on the full cohort.

### 2.7. Reference Models

Three comparators were fitted on identical folds. The first uses only stage, age, sex and anatomical site, which is the information an oncologist already holds before any assay is ordered; a transcriptomic score that merely matches it has added nothing, so this model is both a benchmark and the reference against which incremental value is judged. The second is an elastic-net Cox model [21] at L1 ratios of 0.9 and 0.5, with the penalty chosen by inner three-fold cross-validation along a warm-started descending path, since fitting a single small penalty from a cold start diverges. The third is a random survival forest of 300 trees [44]. Genes entering the penalised and forest models were screened by a univariate Cox score statistic computed inside the training fold only; screening on the assembled cohort is the most common route to an inflated concordance in published expression signatures [10]. The clinical model is a Cox model with a small ridge penalty (0.1) on stage (ordinal), age, sex and anatomical site.

### 2.8. Performance Assessment

Discrimination was measured by the Harrell concordance index [45,46] under repeated stratified five-fold cross-validation with two repetitions, stratified jointly based on event status and anatomical site so that rectal cases, a quarter of the cohort, appear in every fold. Confidence intervals are bootstrap percentiles from 5000 resamples over folds. Fold-level estimates from repeated cross-validation are not independent, because training sets overlap and every patient appears once in each repetition. Models were therefore compared with the corrected resampled *t*-test of Nadeau and Bengio, which inflates the fold-level variance by the ratio of test to training size [47]; naive paired t and Wilcoxon signed-rank tests are reported alongside, together with intervals corrected in the same way. Incremental value was assessed with out-of-fold risk scores, so no patient contributes to the model that scores them. Each patient receives exactly one out-of-fold prediction in each of the two repetitions, each from a network that never saw that patient, and the two were averaged to give one score per patient. Clinical and clinical-plus-molecular Cox models were compared on cross-validated concordance over the same folds, by the likelihood-ratio test, and by the hazard ratio per standard deviation of the score after adjustment for stage, age, sex and site. The concordance gain was also bootstrapped over patients (2000 resamples) from the pooled out-of-fold predictions of the two Cox models, which avoids the dependence between folds. Proportional hazards were checked with scaled Schoenfeld residuals [48] under rank and Kaplan–Meier time transforms, supplemented by models stratified by stage and by a landmark split of follow-up at 24 months, and heterogeneity of the effect across stage was tested by adding a score × stage interaction, with stage ordinal (1 df) and categorical (3 df), and by stage-specific hazard ratios. For external validation, the seed-averaged ensemble was refitted on the entire discovery cohort and its weights frozen. Within each external series, the score was tested by multivariable Cox regression at two levels of adjustment. The primary model adjusted for stage and age where recorded: both in GSE39582 and GSE17536, age alone in GSE87211, which records only post-treatment pathological stage, and no covariate in GSE33113, which is restricted to stage II and records no age. The extended model added every further variable the series records: sex and tumour site in GSE39582, sex in GSE17536 and GSE33113, and sex with post-treatment ypT, ypN and M category in GSE87211; patients missing any of these were excluded. In the extended model, clinical-only and clinical-plus-score models were compared by the likelihood-ratio test and by concordance under 20 repeats of five-fold cross-validation in which only the Cox coefficients were refitted. Outcome was also compared across tertiles of the score in each external cohort.

### 2.9. Interpretation of the Fitted Network

Because every retained connection carries a function rather than a coefficient, the fitted model reports the shape of each gene’s contribution. Edge influence was quantified as the amplitude of the learned function over the observed expression range,

(6) $$A_{ij}={max}_{x}\varphi_{ij}\left(x\right)-{min}_{x}\varphi_{ij}\left(x\right)$$

and gene-level influence as the gated sum of these amplitudes. Ranking genes on that sum alone rewards connectivity: a gene belonging to three hundred pathways accumulates more total influence than an equally informative gene belonging to four, and the resulting list fills with ubiquitin and proteasome subunits. Dividing by edge count overcorrects in the opposite direction and promotes genes that belong to a single set. Genes are therefore scored against others of comparable connectivity by standardising within bins of log degree,

(7) $$S_{g}=\sum_{j\in N\left(g\right)}|g_{gj}|A_{gj},\;z_{g}=\frac{S_{g}-\mu_{b}}{\sigma_{b}}$$

where *μ* and *σ* are the mean and standard deviation of the summed influence within the degree bin *b* containing gene *g*. Genes were ordered by log2 degree and divided into 12 bins of equal size (812 or 813 genes); because many genes share a degree, ties were broken alphabetically, so that genes of the lowest degrees span more than one bin. Pathways were ranked the same way, standardised within eight equal-size bins of set size. Enrichment of the ranked genes for the held-out cancer gene lists was tested both by Fisher’s exact test and against a degree-matched permutation null of 2000 to 5000 random gene sets drawn to reproduce the connectivity profile of the selected genes. The second test is the informative one. Curated cancer gene lists are themselves enriched for well-studied, heavily connected genes, so any ranking with a connectivity bias will clear the naive threshold whether or not it has learned anything. Because the binning is a modelling choice, the ranking and the enrichment tests were repeated with 4 to 24 equal-count bins, with bins that never separate genes of equal degree, and with a continuous standardisation against the *k* genes of nearest degree (*k* = 250, 500 and 1000), which uses no bins at all. Stability of the ranking and of the learned functions was assessed across the ten cross-validation networks, which differ in training data, the five seeds of the final ensemble, which differ in initialisation, and 20 bootstrap refits of the final configuration. An edge function is an internal component of a multilayer network: a gene can act through several pathways and share predictive information with correlated genes. For the genes discussed in the text, we therefore also computed the partial dependence of the predicted log-hazard, the mean change in model output as one gene’s input is moved across its range with every other input held at its observed value.

### 2.10. Colorectal Cancer Relevance

Pan-cancer driver lists answer whether the selected genes are known oncogenes, not whether they matter in this disease. Four further analyses addressed the second question. COSMIC records the tumour types each census gene is implicated in; restricting to entries annotated for colorectal, colonic, large intestinal, bowel or rectal tumours gives 86 genes, of which 70 fall in the modelled space, and enrichment was retested against the same degree-matched null. Second, each prioritised gene was compared between tumour and normal mucosa using all 647 primary-tumour aliquots retrieved from TCGA-COAD and TCGA-READ, from 624 patients, and the 51 adjacent normal samples, from 51 patients. This comparison uses no outcome data and was therefore not restricted to the discovery cohort, which excludes 28 patients without positive progression-free follow-up and keeps one aliquot per patient (Section 2.1); it was repeated on the 596 discovery tumours as a sensitivity analysis. Genes were compared by Mann–Whitney test with Benjamini–Hochberg control of the false discovery rate [49]. Third, pathway activity was scored as the mean standardised expression of member genes and compared across microsatellite instability status and TCGA expression subtype by Kruskal–Wallis test. Because both variables were also auxiliary training targets (Section 2.5.2), this comparison is descriptive and not independent of the fitting. Fourth, to ask whether the score reflects a prognostic programme shared across tumour types, the frozen ensemble was applied, exactly as it was to the validation series, to the TCGA oesophageal carcinoma (ESCA; 184 patients, 87 events) and lung adenocarcinoma (LUAD; 504 patients, 207 events) cohorts processed through the same GDC pipeline, with the TCGA-CDR progression-free interval as outcome [25] and adjustment for stage and age.

### 2.11. Software and Reproducibility

Retrieval and normalisation used R 4.6.0 with TCGAbiolinks 2.39.0 and DESeq2 1.51.7. Modelling used Python 3.12 (Python Software Foundation, Wilmington, DE, USA) with PyTorch 2.13 under CUDA 13.0, built on NumPy [50] and SciPy [51], with survival analysis through scikit-survival and lifelines [52], on a single NVIDIA RTX 4060. A fixed random seed governs every split, initialisation and permutation. All primary and validation data are public, and a single driver script regenerates every result from the raw downloads.

## 3. Results

### 3.1. A Pooled Colorectal Cohort with Twice Its Own Event Count in Validation

**Figure 1:**
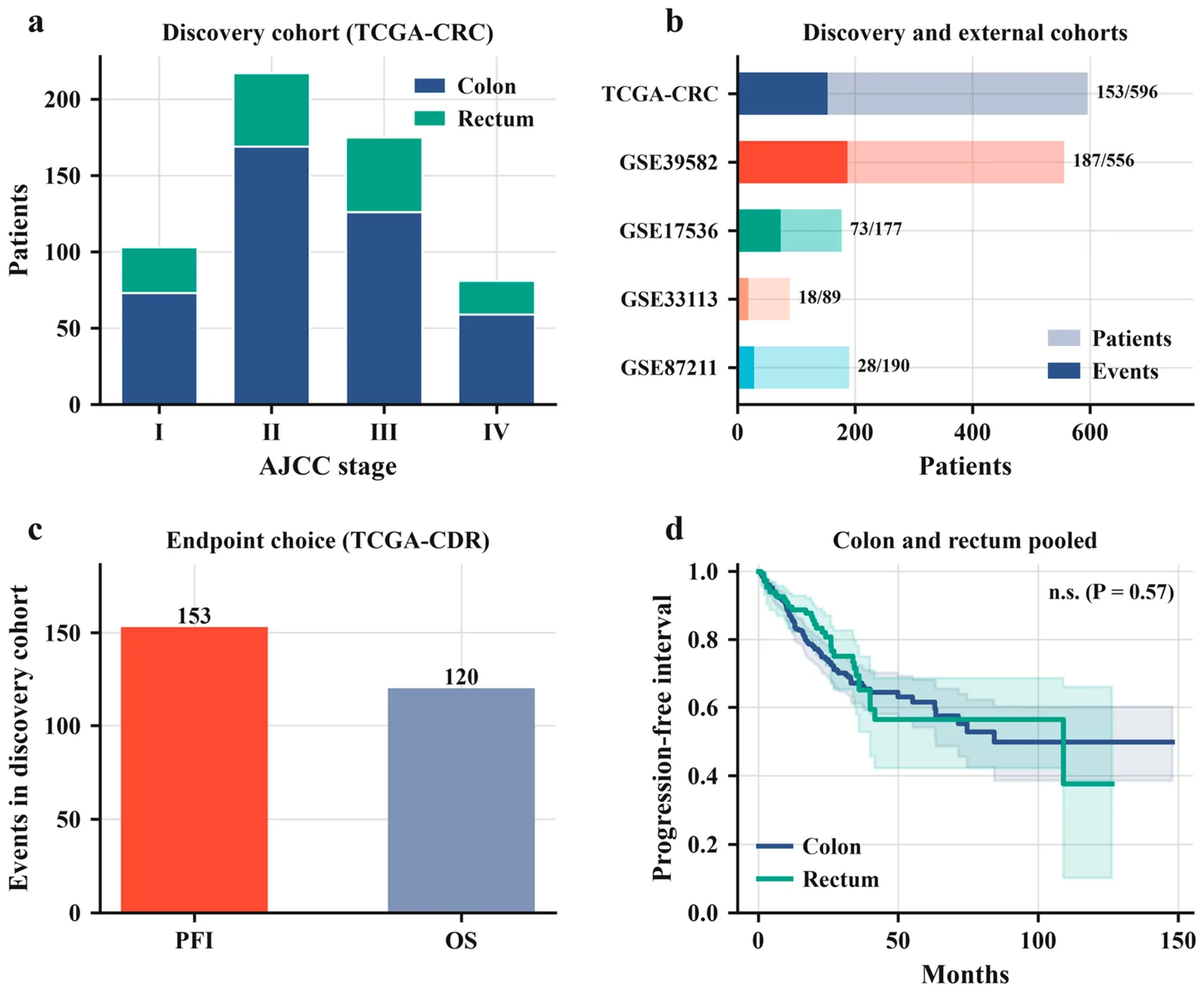
Study design and cohorts. (**a**) Stage and anatomical site composition of the pooled TCGA colorectal discovery cohort. (**b**) Patients (pale) and events (solid) in the discovery cohort and in the four independent validation series, with each cohort shown in a different colour; the external cohorts contribute twice the discovery event count. (**c**) The progression-free interval endpoint recovers 153 events against 120 for overall survival in the same patients (red, progression-free interval; grey, overall survival); overall-survival events are counted among patients with a positive survival time, and 4 of the 124 deaths recorded in Table 1 lack one. (**d**) Progression-free interval does not differ between colon and rectum, supporting analysis as a single disease.

The merged TCGA discovery set comprises 596 patients, 438 colonic and 158 rectal, with 153 progression-free events (25.7%) and a median age of 68 (Table 1). With stage missing from the GDC record, completed from the TCGA Clinical Data Resource, stage II and stage III disease together account for 66% of cases, which is the population in which a prognostic marker would actually be used, and 20 patients (3.4%) remain unstaged. Progression-free interval did not differ between colon and rectum (log-rank *p* = 0.57, Figure 1d), supporting the decision to analyse the two sites as one disease rather than splitting an already modest cohort in two. Quantile alignment of the two variance-stabilised blocks is shown in Figure S1. Choosing progression-free interval over overall survival recovered more than a quarter more events, 153 against 120 analysable overall-survival events (Figure 1c and Figure S2), and confined those events to disease progression rather than to deaths from any cause in a cohort with a median age of 68. Four independent series supplied 1012 further patients carrying 306 events, twice the discovery total (Figure 1b, Table 2). Their provenance is recorded in Table S1. Two array platforms and three distinct endpoints are represented, so the validation is a test of transportability and not a second look at the same data.

**Table 1:** Characteristics of the pooled TCGA colorectal discovery cohort (*n* = 596). Vital status lists every recorded death; 120 of the 124 have a positive overall-survival time and enter the survival analyses.

Variable	Level	n	%
N		596	
Age, median (IQR)		68	58–75
Sex	male	322	54.0%
Sex	female	274	46.0%
Tissue site	colon	438	73.5%
Tissue site	rectum	158	26.5%
AJCC stage	II	217	36.4%
AJCC stage	III	175	29.4%
AJCC stage	I	103	17.3%
AJCC stage	IV	81	13.6%
AJCC stage	not available	20	3.4%
MSI status		343	57.6%
MSI status	MSS	176	29.5%
MSI status	MSI-L	41	6.9%
MSI status	MSI-H	36	6.0%
Expression subtype		395	66.3%
Expression subtype	CIN	79	13.3%
Expression subtype	MSI/CIMP	66	11.1%
Expression subtype	Invasive	56	9.4%
Vital status	Alive	472	79.2%
Vital status	Dead	124	20.8%
PFI events		153	25.7%

**Table 2:** Independent validation cohorts. Endpoints differ between series (OS, overall survival; DFS, disease-free survival; DSS, disease-specific survival), and the two platforms are Affymetrix GPL570 and Agilent GPL13497.

GEO Accession	Platform	Endpoint	N	Events	Event Rate	Median Follow-Up (Months)
GSE39582	GPL570	OS	556	187	33.6%	52.0
GSE17536	GPL570	OS	177	73	41.2%	42.3
GSE33113	GPL570	DFS	89	18	20.2%	38.9
GSE87211	GPL13497	DSS	190	28	14.7%	63.5

### 3.2. Prior Knowledge Removes Almost All of a Dense Network

**Figure 2:**
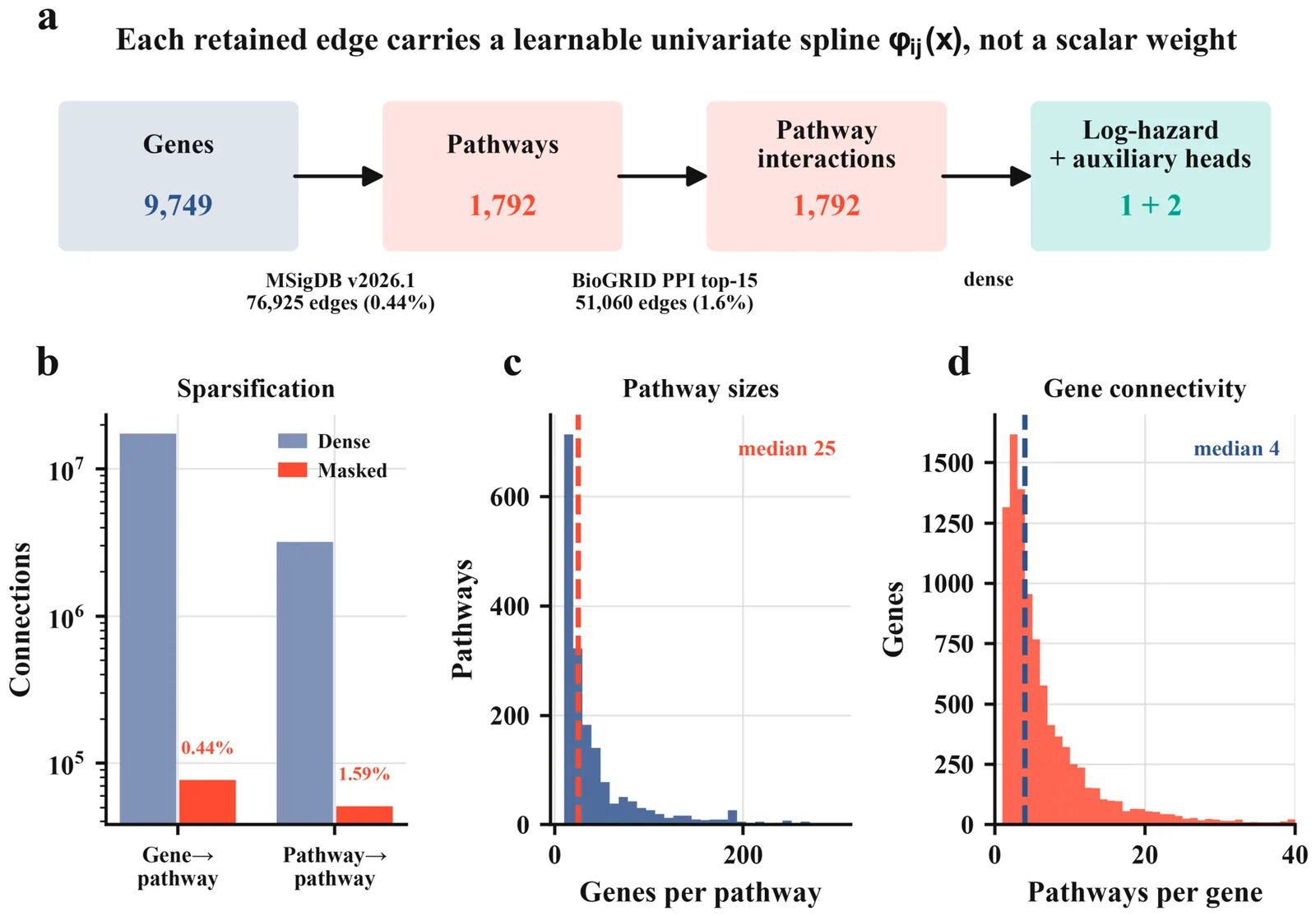
PathKAN architecture and prior-knowledge sparsification. (**a**) Genes feed pathways, pathways feed a pathway-interaction layer, and a bias-free map returns a log-hazard; every retained connection carries a learnable univariate spline rather than a scalar weight. (**b**) Masked against dense connection counts on a logarithmic scale. (**c**) Distribution of pathway sizes. (**d**) Distribution of gene connectivity.

After restricting to transcripts measured on the sequencing platform and on every validation array, 9749 genes and 1792 pathways remained, joined by 76,925 gene-to-pathway edges. A dense layer over the same dimensions would carry 17,470,208 weights, so the incidence mask retains 0.44% of them. The pathway-interaction layer keeps 51,060 of 3,211,264 possible links, or 1.59%. Across both layers, the model holds 127,985 connections where an unconstrained network would hold 20,681,472, a reduction of more than 160-fold (Figure 2b). The retained topology is not uniform. Pathways carry a median of 25 genes and genes belong to a median of 4 pathways, with a long tail of highly connected transcripts (Figure 2c,d and Figure S3). That tail later turns out to matter for how gene importance must be scored.

### 3.3. Standalone Discrimination Was Modest and Similar Across Molecular Models

**Figure 3:**
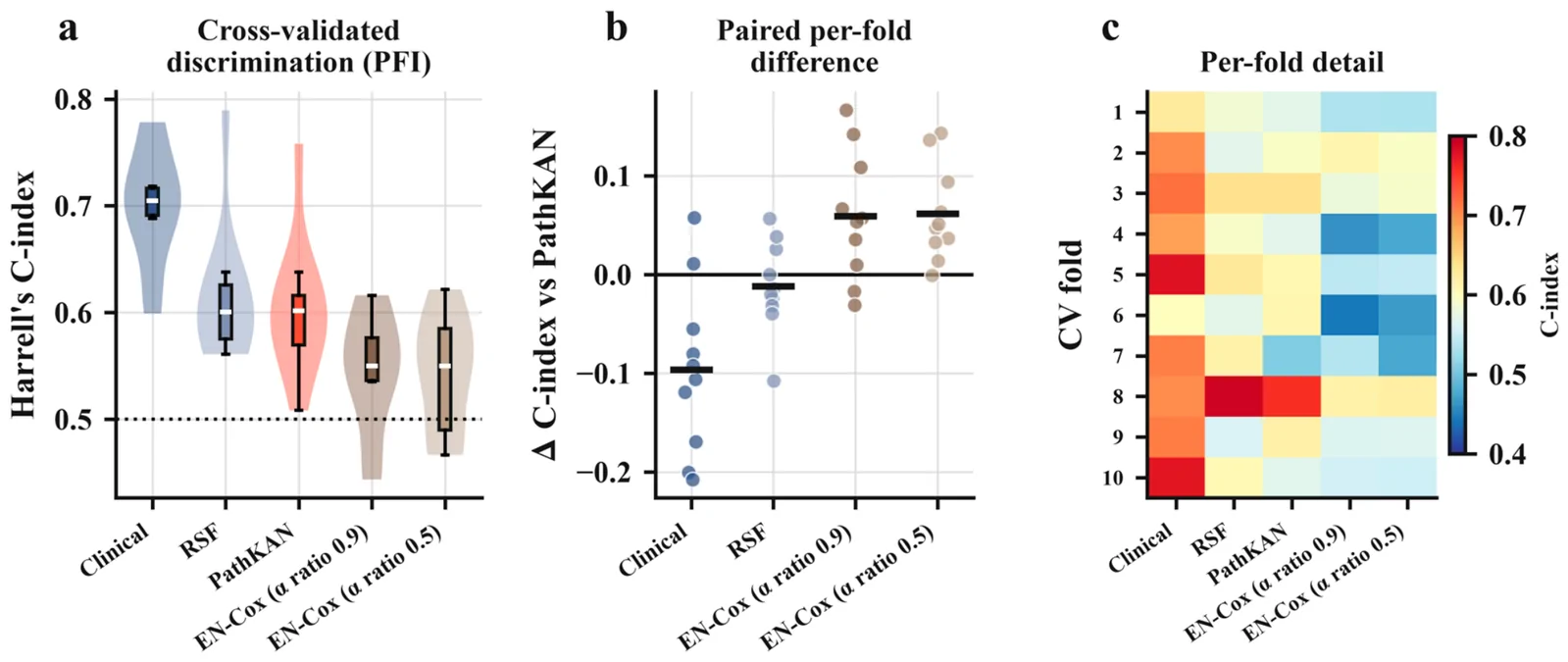
Cross-validated discrimination on the discovery cohort under nested, repeated stratified five-fold cross-validation. (**a**) Fold distributions for every model. (**b**) Paired per-fold differences against PathKAN; the horizontal bar marks the mean. (**c**) Per-fold concordance for all five models on identical splits. PathKAN hyperparameters were tuned within each outer training partition; the clinical model uses stage completed from the TCGA Clinical Data Resource.

Under nested, repeated stratified cross-validation, PathKAN reached a concordance index of 0.604 (SD 0.065), similar to the random survival forest at 0.616 (SD 0.066) and above both penalised Cox models (Table 3, Figure 3a). The clinical model, with stage completed from the TCGA Clinical Data Resource, reached 0.700 (SD 0.056) and outperformed every molecular model on its own. The fold-wise advantage of PathKAN over elastic-net Cox (ΔC = +0.059 and +0.062) was significant on naive paired tests (*p* = 0.018 and 0.003) but not after correction for the overlap between cross-validation training sets (corrected *p* = 0.16 and 0.060), and differences from the forest fell well inside the fold-to-fold variation (Table 4 and Table S16, Figure 3b). Per-fold values for every model are given in Figure 3c and Table S3. Tuning inside each outer training partition changed the estimate little: the non-nested search had given 0.606, and the configuration chosen differed between outer folds (Table S15). This agrees with the flat tuning surface, on which the full 24-configuration grid spanned 0.593 to 0.608 (Figure S4, Table S2). A flat surface shows that these hyperparameters matter little at this sample size, but on its own it does not show what limits performance. A learning curve does not support the view that the number of events limits performance within the range we could examine: subsampling every outer training partition from 40% to 100% (on average 49 to 122 training events) left PathKAN concordance essentially unchanged (0.597 to 0.606; slope +0.010 per doubling of events, *p* = 0.61), as it did for the clinical model (Figure S11, Table S26). Whether much larger cohorts would help cannot be judged from these data. The question pursued here is therefore not which model scores highest on its own, but whether the molecular score adds to what the clinic already knows.

**Table 3:** Cross-validated discrimination for the progression-free interval endpoint. Confidence intervals are bootstrap percentiles over folds; intervals corrected for the dependence between folds are given in Table S17. PathKAN was tuned by nested cross-validation, and the clinical model uses stage completed from the TCGA Clinical Data Resource.

Model	C-Index (Mean)	SD	Median	95% CI Lower	95% CI Upper
Clinical only	0.700	0.056	0.705	0.667	0.732
Random survival forest	0.616	0.066	0.600	0.585	0.659
PathKAN	0.604	0.065	0.602	0.569	0.645
Elastic-net Cox (α ratio 0.9)	0.545	0.057	0.550	0.511	0.576
Elastic-net Cox (α ratio 0.5)	0.542	0.055	0.550	0.509	0.574

**Table 4:** Paired comparison of PathKAN against each reference model on the shared cross-validation folds (ten folds). The corrected *t*-test inflates the fold-level variance for the overlap between training sets (Nadeau–Bengio).

Comparison	Mean ΔC-Index	*p* (Paired t)	*p* (Wilcoxon)	*p* (Corrected t)
PathKAN vs. clinical	−0.0962	0.0063	0.0137	0.0908
PathKAN vs. random survival forest	−0.0116	0.4482	0.6523	0.6816
PathKAN vs. elastic-net Cox (α ratio 0.9)	+0.0592	0.0179	0.0195	0.1570
PathKAN vs. elastic-net Cox (α ratio 0.5)	+0.0618	0.0030	0.0039	0.0604

### 3.4. The Score Is Associated with Progression Independently of Clinical Variables, with a Small Gain in Discrimination

**Figure 4:**
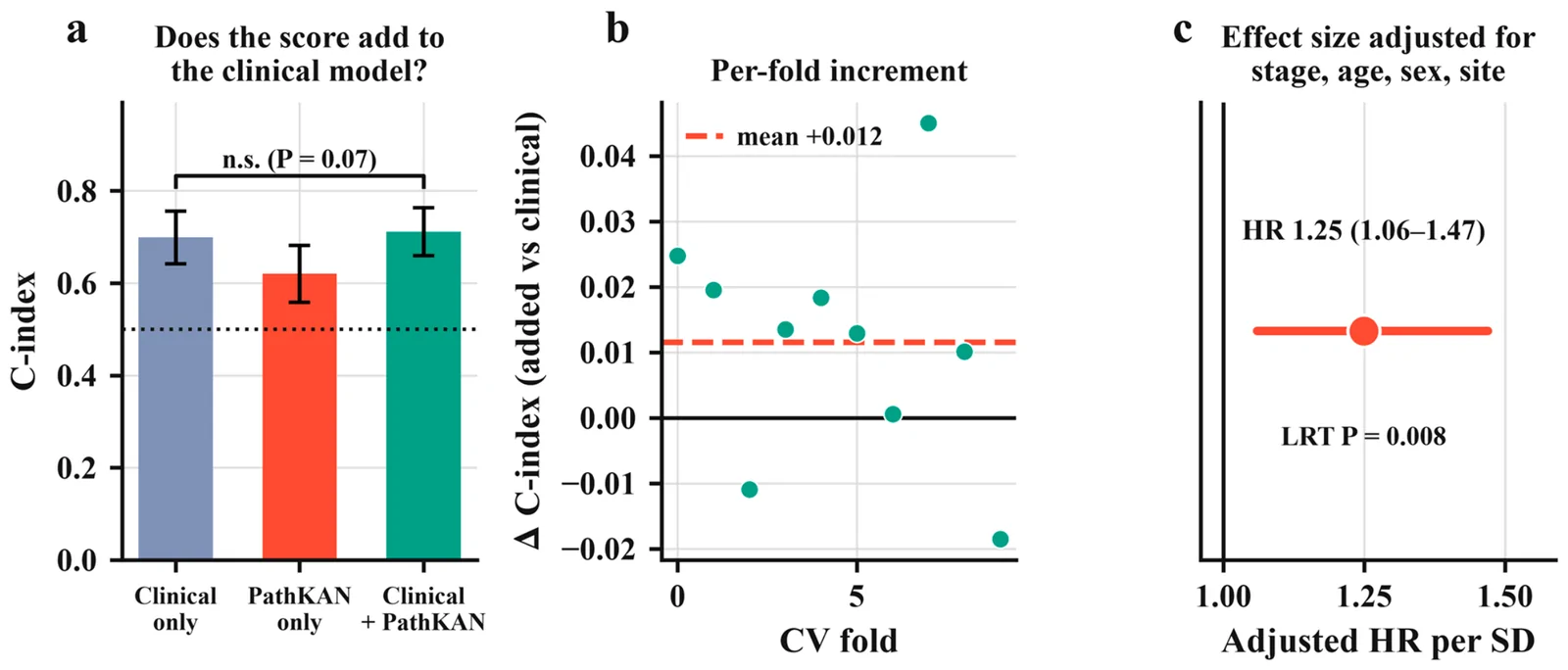
Incremental value over the clinical model, computed from out-of-fold scores so that no patient contributes to the model that scores them. (**a**) Concordance for the clinical model, PathKAN alone, and the two combined, with standard deviations across folds. (**b**) Per-fold gain from adding the molecular score. (**c**) Adjusted hazard ratio per standard deviation of the score, with the likelihood-ratio test for its addition. The clinical model uses stage completed from the TCGA Clinical Data Resource, and the score comes from nested cross-validation.

Stage, age, sex and anatomical site together reached a concordance of 0.699. Adding the out-of-fold PathKAN score raised it to 0.711, a gain of 0.012 that was positive in 8 of ten folds but was not statistically supported (paired *p* = 0.075; corrected *p* = 0.31). A patient-level bootstrap of the pooled out-of-fold predictions gave a gain of 0.012 (95% CI −0.004 to 0.028) (Figure 4a,b, Table 5, Tables S4 and S16). The score nevertheless improved model fit (likelihood-ratio chi-square 7.14, *p* = 0.008), and each standard deviation carried a hazard ratio of 1.25 (95% CI 1.06–1.47) after adjustment for stage, age, sex and site (Figure 4c). The score is therefore independently associated with outcome, but its contribution to discrimination over a clinical model that already reaches 0.699 is small. Scaled Schoenfeld residuals gave no evidence of non-proportional hazards for any clinical covariate, but borderline evidence for the score (*p* = 0.051 with rank and 0.031 with Kaplan–Meier time). Split at 24 months, the adjusted hazard ratio was 1.32 (1.09–1.59) for the 115 early events and 1.01 (0.72–1.41) for the 38 later ones. This decline was not seen in GSE39582, where the hazard ratio after 24 months was 1.26 (*p* = 0.013), and stratifying the baseline hazard by stage left the hazard ratio unchanged in every cohort (Tables S18–S20). The association may therefore be concentrated in early progression, but the data do not establish this. The association did not depend on how unstaged patients were handled: with a missing-stage indicator, with stage as a categorical variable, or among the 576 patients with known stage, the adjusted hazard ratio ranged from 1.22 to 1.28 and the likelihood-ratio *p* from 0.004 to 0.017 (Table S12). Imputing the median stage for the 112 patients without a GDC stage, instead of using the curated stage, weakened the clinical model to a concordance of 0.602 and inflated the apparent gain to 0.038 (Table S12), which shows how strongly incremental-value estimates depend on the completeness of the clinical baseline. Splitting the discovery cohort by out-of-fold risk tertile separated progression-free interval (log-rank *p* = 2.2 × 10^−4^).

**Table 5:** Incremental value of the PathKAN score over a clinical model containing stage, age, sex and anatomical site, evaluated on identical folds. *p* (paired t) is shown naive and, in parentheses, corrected for the dependence between folds. Stage was completed from the TCGA Clinical Data Resource. The PathKAN-only row uses the out-of-fold score averaged over the two repetitions, whereas Table 3 reports each fold’s network on its own test fold; averaging two independent predictions explains the small difference between the two.

Model	C-Index (Mean)	SD	Δ vs. Clinical	*p*, Paired t (Corrected)	LRT χ^2^	LRT *p*	HR per SD	HR 95% CI
Clinical only	0.699	0.057	0.000					
PathKAN only	0.620	0.062	−0.079					
Clinical + PathKAN	0.711	0.052	0.012	0.075 (0.309)	7.14	0.0075	1.249	1.06–1.47

### 3.5. The Frozen Model Remained Prognostic in Two of Four External Cohorts

**Figure 5:**
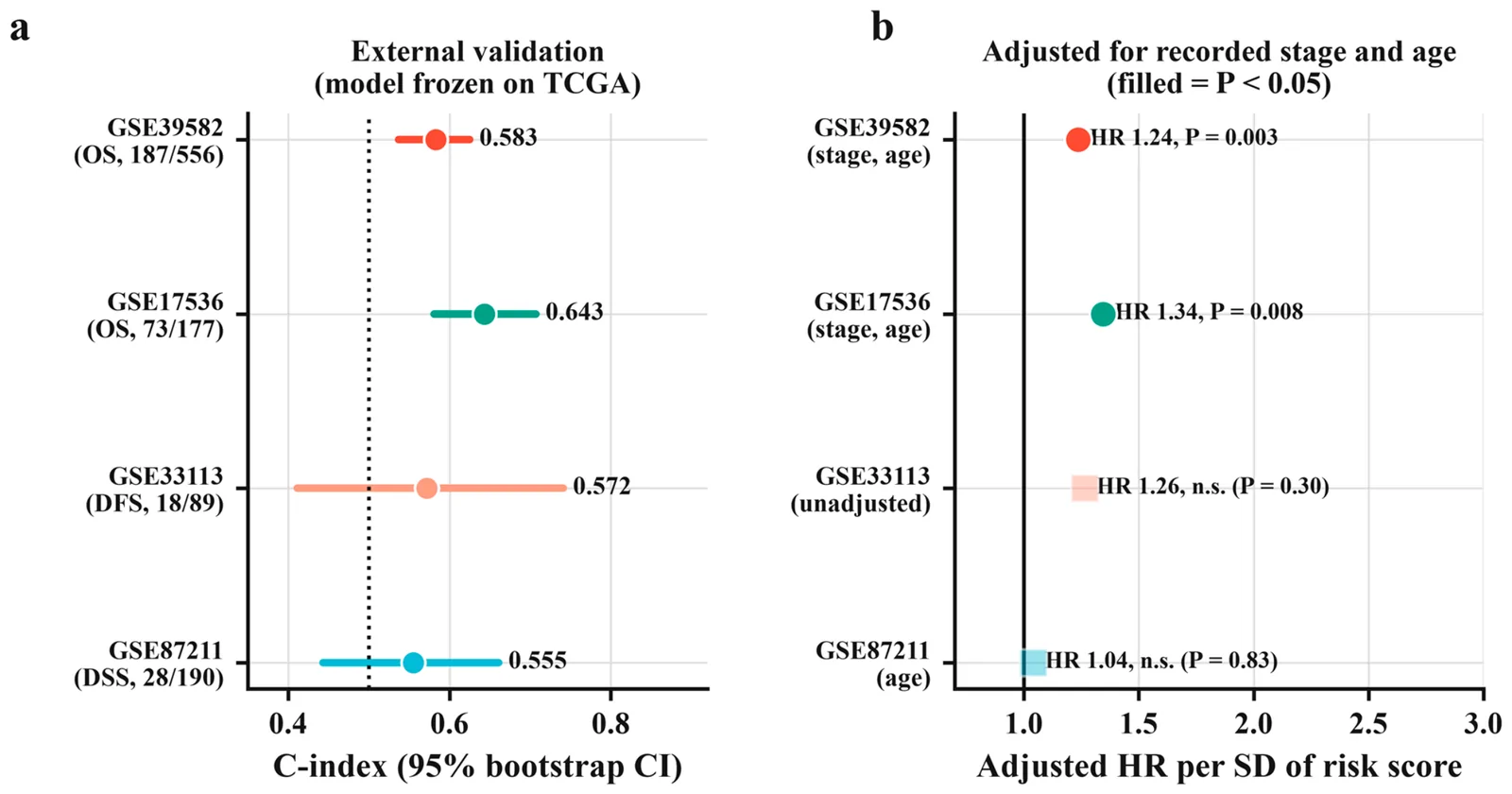
External validation of the frozen model in four independent cohorts. (**a**) Concordance with 95% bootstrap intervals; the dotted line marks chance. (**b**) Hazard ratio per standard deviation of the risk score after adjustment for the stage and age recorded in each cohort (stage and age in GSE39582 and GSE17536, age in GSE87211, none in GSE33113, which is restricted to stage II and records no age; Table 6), with filled markers denoting *p* < 0.05. Hazard ratios under extended adjustment are given in Table S13.

The seed-averaged ensemble was refitted on the whole discovery cohort, its weights frozen, and applied to each external series after the within-cohort input standardisation described in Section 2.3. Because the input space had been restricted to transcripts common to all platforms, gene coverage was complete in every cohort, and no value had to be imputed (Table 6). In GSE39582, the largest series at 556 patients and 187 events, the score reached a concordance of 0.583 (95% CI 0.537–0.625) and remained an independent predictor after adjustment for stage and age (HR 1.24 per standard deviation, *p* = 0.003). In GSE17536, it reached 0.643 (95% CI 0.581–0.707) with an adjusted hazard ratio of 1.34 (*p* = 0.008). Adjustment for the extended covariate set, adding sex and tumour site in GSE39582 and sex in GSE17536, gave hazard ratios of 1.22 (*p* = 0.005) and 1.34 (*p* = 0.010) (Table S13). The association was therefore replicated in the two larger treatment-naive cohorts, on a different platform, continent and endpoint from those the model was trained on (Figure 5). In both, adding the score to the clinical variables recorded in that cohort improved model fit (likelihood-ratio *p* = 0.005 and 0.010), and cross-validated concordance rose by 0.007 and 0.006, gains as small as in the discovery cohort (Table S13). In the two remaining series, the evidence is inconclusive: GSE33113 and GSE87211 carry only 18 and 28 events, their concordance intervals include 0.5, and their hazard ratios were not significant under either level of adjustment (Table 6, Table S13). GSE87211 differs further in that every patient received preoperative chemoradiation, so it tests prognosis after treatment rather than the treatment-naive question the model addresses; it is examined separately in Figure S7. The scale of the three platforms differs substantially (Figure S6), which is what the within-sample rank representation was designed to absorb. Per-gene centring and scaling within each external cohort is nevertheless part of the transfer. With these parameters fixed at the discovery-cohort values, the association persisted in GSE17536 (concordance 0.601; adjusted HR 1.32, *p* = 0.008) but was attenuated and no longer significant in GSE39582 (concordance 0.554; adjusted HR 1.08, *p* = 0.28) (Table S13). The transportability reported here is therefore conditional. The network weights transfer unchanged, but the inputs depend on an outcome-blind standardisation estimated at the cohort level, and in the largest cohort, the association was materially weaker without it. A single future patient brings no cohort from which to estimate these parameters, so the present results do not yet describe a score that can be applied to an individual patient; that would require a fixed external standardisation reference to be established and shown to reproduce the within-cohort results.

**Table 6:** External validation of the frozen model. Gene coverage is complete in every cohort because the input space was restricted to transcripts measured on all platforms. Network weights are frozen; per-gene centring and scaling were estimated within each cohort without outcome data (results with discovery-fixed scaling and clinical-only comparisons in Table S13). The reported transportability is therefore conditional on this cohort-level step and does not yet extend to individual patients. The hazard ratio is adjusted for the covariates listed: GSE33113 contains only stage II tumours and records no age, and GSE87211 records only post-treatment pathological stage. Under the extended adjustment of Table S13 it was 1.22 (*p* = 0.005; stage, age, sex and site; *n* = 551) in GSE39582, 1.34 (*p* = 0.010; stage, age and sex) in GSE17536, 1.26 (*p* = 0.30; sex) in GSE33113 and 0.95 (*p* = 0.81; age, sex, ypT, ypN and M; *n* = 189) in GSE87211.

GEO Accession	Endpoint	N	Events	Gene Coverage	C-Index (95% CI)	Adjusted HR per SD	*p*	Adjusted for
GSE39582	OS	556	187	100.0%	0.583 (0.537–0.625)	1.24	0.003	stage + age
GSE17536	OS	177	73	100.0%	0.643 (0.581–0.707)	1.34	0.0085	stage + age
GSE33113	DFS	89	18	100.0%	0.572 (0.411–0.741)	1.26	0.2985	none
GSE87211	DSS	190	28	100.0%	0.555 (0.443–0.661)	1.04	0.8341	age

### 3.6. Risk Tertiles Separate Outcome in the Two Larger Validation Cohorts

**Figure 6:**
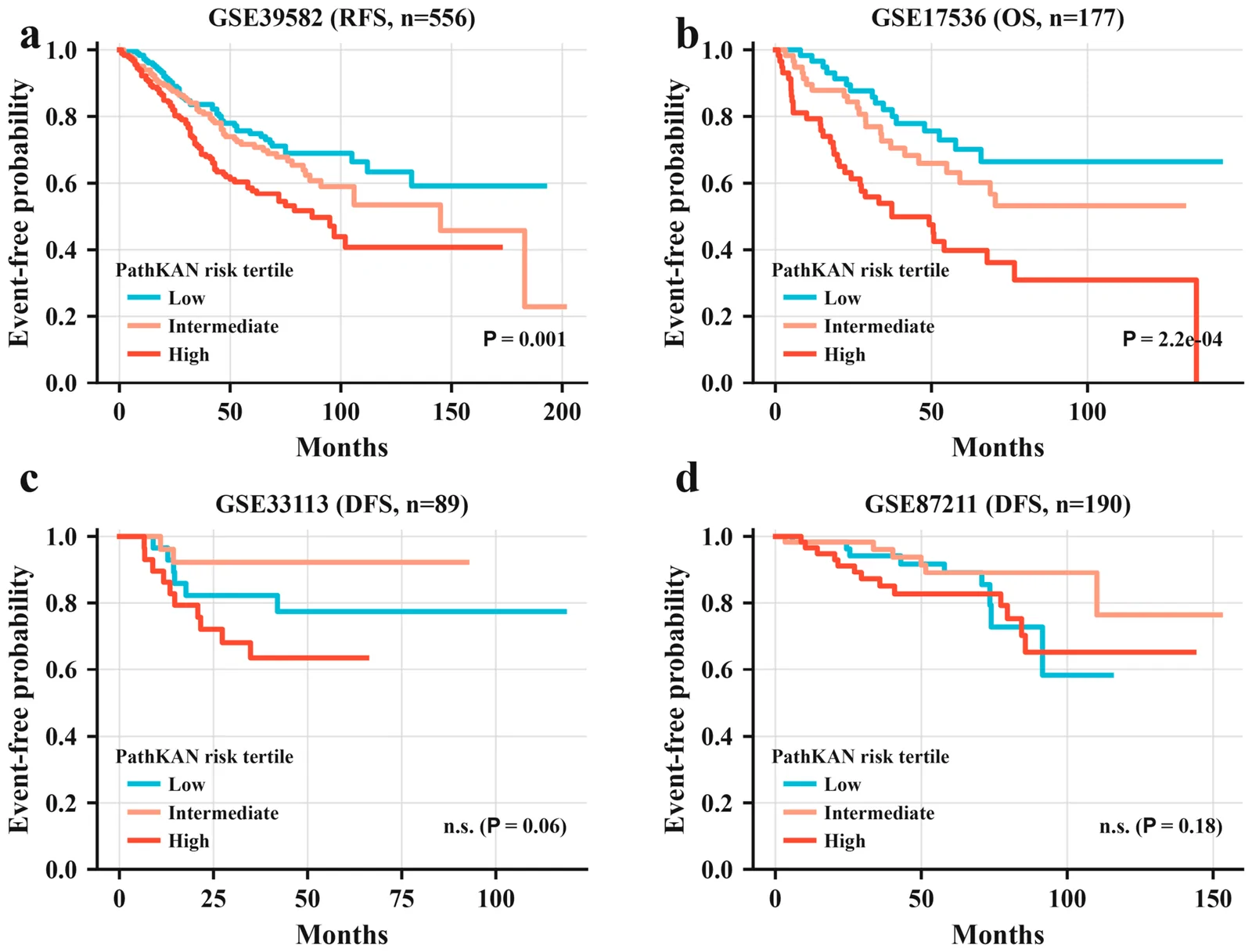
Risk stratification by tertile of the frozen PathKAN score in each external cohort. (**a**) GSE39582, (**b**) GSE17536, (**c**) GSE33113, and (**d**) GSE87211. Endpoints differ between series as listed in Table 2.

Dividing each external cohort at the tertiles of the frozen score produced ordered survival curves in the two large treatment-naive series, with the high-risk third separating early and remaining separated throughout follow-up (Figure 6). In the two smaller cohorts, with 18 and 28 events, the data neither support nor exclude an association. Risk-score distributions in patients with and without events are shown in Figure S8.

### 3.7. The Model Exposes the Shape of Each Learned Transformation

**Figure 7:**
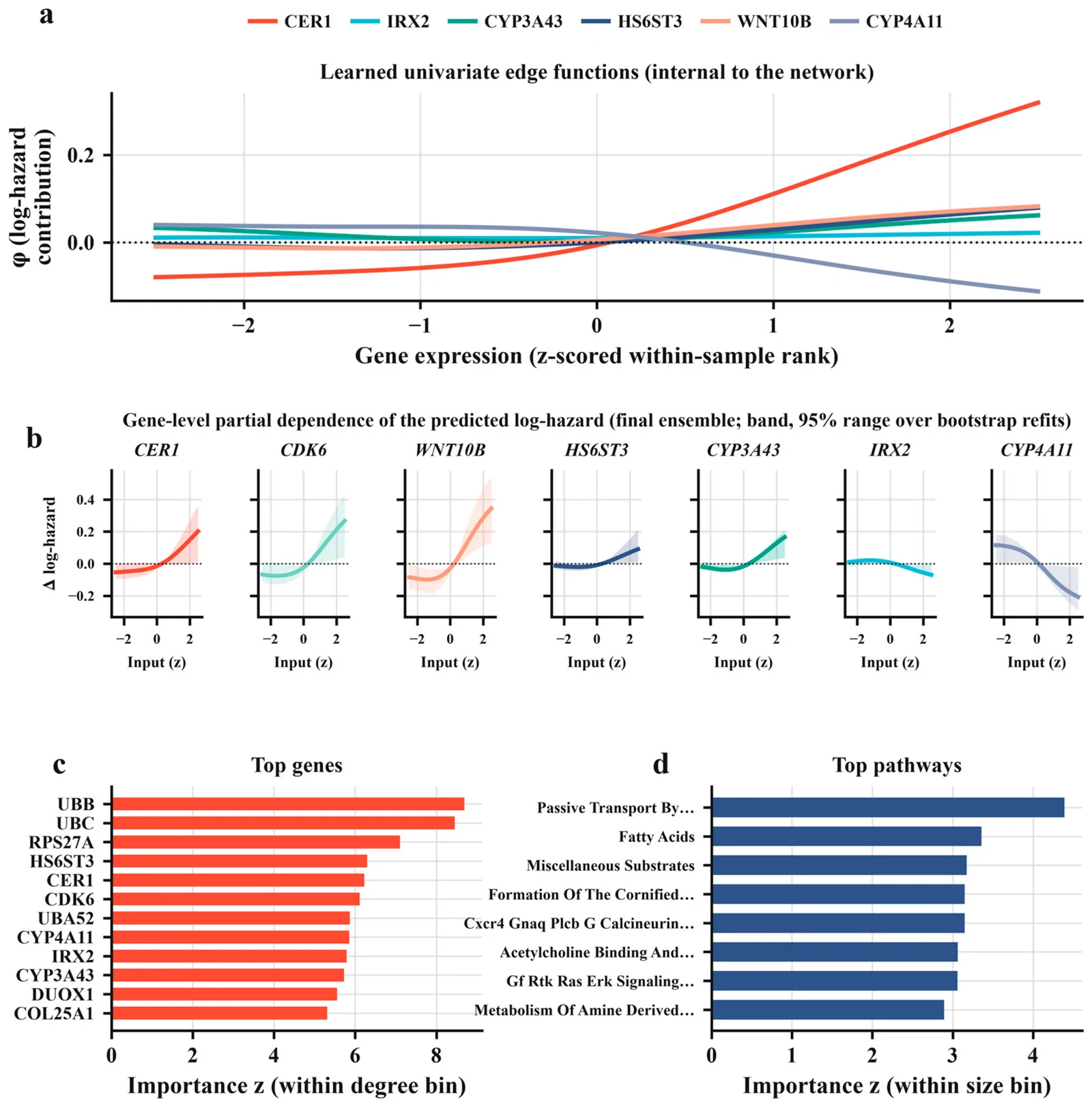
Interpretability of the fitted network. (**a**) Learned univariate edge functions for six representative genes in the final ensemble, showing the transformation learned on each gene-to-pathway edge across the observed expression range; these are internal functions of the network, not direct gene effects, and their stability is shown in Figure S12. (**b**) Gene-level partial dependence of the predicted log-hazard for seven of the genes discussed in the text: the mean change in the output of the final ensemble as one gene’s standardised input is moved across its range with all other inputs at their observed values (line), with the 2.5–97.5 percentile range across 20 bootstrap refits (band). This is the stable quantity on which the biological interpretation rests. (**c**) Highest-ranked genes after residualisation on connectivity. (**d**) Highest-ranked pathways after residualisation on set size; long pathway names are truncated in the panel and are given in full in Table S6.

Because every retained connection carries a function rather than a coefficient, the fitted network exposes the transformation learned on each gene-to-pathway edge. In the final ensemble, the learned edge functions were not uniformly monotonic (Figure 7a): the CER1 edge contributed little across most of its range and rose above the cohort median, and the CYP4A11 edge changed direction across its upper range. As individual quantities, however, edge functions were not reproducible. Across the ten cross-validation networks, four of the six edges in Figure 7a kept their shape (median correlation with the ensemble curve above 0.9), but across bootstrap refits, three of the six reversed sign at the median, because downstream layers can compensate for one another (Figure S12a). The gene-level partial dependence of the predicted log-hazard, which integrates all of a gene’s edges and the downstream layers, was far more stable (median correlation with the ensemble curve at least 0.97 in every group of refits; Figure 7b and Figure S12b). At that level, the relationships were smooth and mostly monotone: predicted risk rose with CER1, CDK6 and WNT10B and fell with CYP4A11, with steeper change above the median for CER1 and shallow J-shapes for HS6ST3, CYP3A43 and WNT10B. Effects were modest, between 0.05 and 0.37 on the log-hazard scale across ±2.5 standard deviations of expression. Ranking genes required care. Summed edge influence rewards connectivity, filling the list with ubiquitin and proteasome subunits that belong to more than three hundred pathways each; dividing by edge count overcorrects and promotes genes belonging to a single set. Scoring each gene against others of comparable connectivity resolves both failure modes, and the resulting list mixes those high-degree transcripts with specific low-degree candidates such as HS6ST3, CER1, CYP4A11 and IRX2, alongside the cell-cycle regulator CDK6 (Table 7 and Table S5). The four high-connectivity ubiquitin genes ranked within the top 50 in every refit, and CER1 and CDK6 were usually within the top 500 (CER1 in 10/10 cross-validation networks and 10/20 bootstrap refits; CDK6 in 8/10 and 18/20), whereas most of the remaining low-degree genes were not consistently reproduced (Table S24, Figure S12c). Two refits shared on average 8 of their top 50 genes, against 0.26 expected by chance. At the pathway level, aquaporin-mediated transport, fatty-acid metabolism and cornified-envelope formation ranked highest once set size was accounted for (Figure 7d, Table S6). Influence values for the 2000 strongest individual connections are given in Table S7.

**Table 7:** The fifteen highest-ranked genes after residualising influence on pathway connectivity. The full ranking is given in Table S5.

Rank	Gene	Importance z	Pathway Edges	COSMIC CGC	OncoKB
1	UBB	8.69	324	No	No
2	UBC	8.453	312	No	No
3	RPS27A	7.105	325	No	No
4	HS6ST3	6.296	4	No	No
5	CER1	6.227	7	No	No
6	CDK6	6.109	18	Yes	Yes
7	UBA52	5.867	322	No	No
8	CYP4A11	5.855	11	No	No
9	IRX2	5.793	2	No	No
10	CYP3A43	5.727	5	No	No
11	DUOX1	5.554	2	No	No
12	COL25A1	5.31	5	No	No
13	GATM	5.091	1	No	No
14	DNAH10	5.036	3	No	No
15	OTUD1	4.949	4	No	No

### 3.8. The Selected Genes and Pathways Relate to Colorectal Cancer Biology

**Figure 8:**
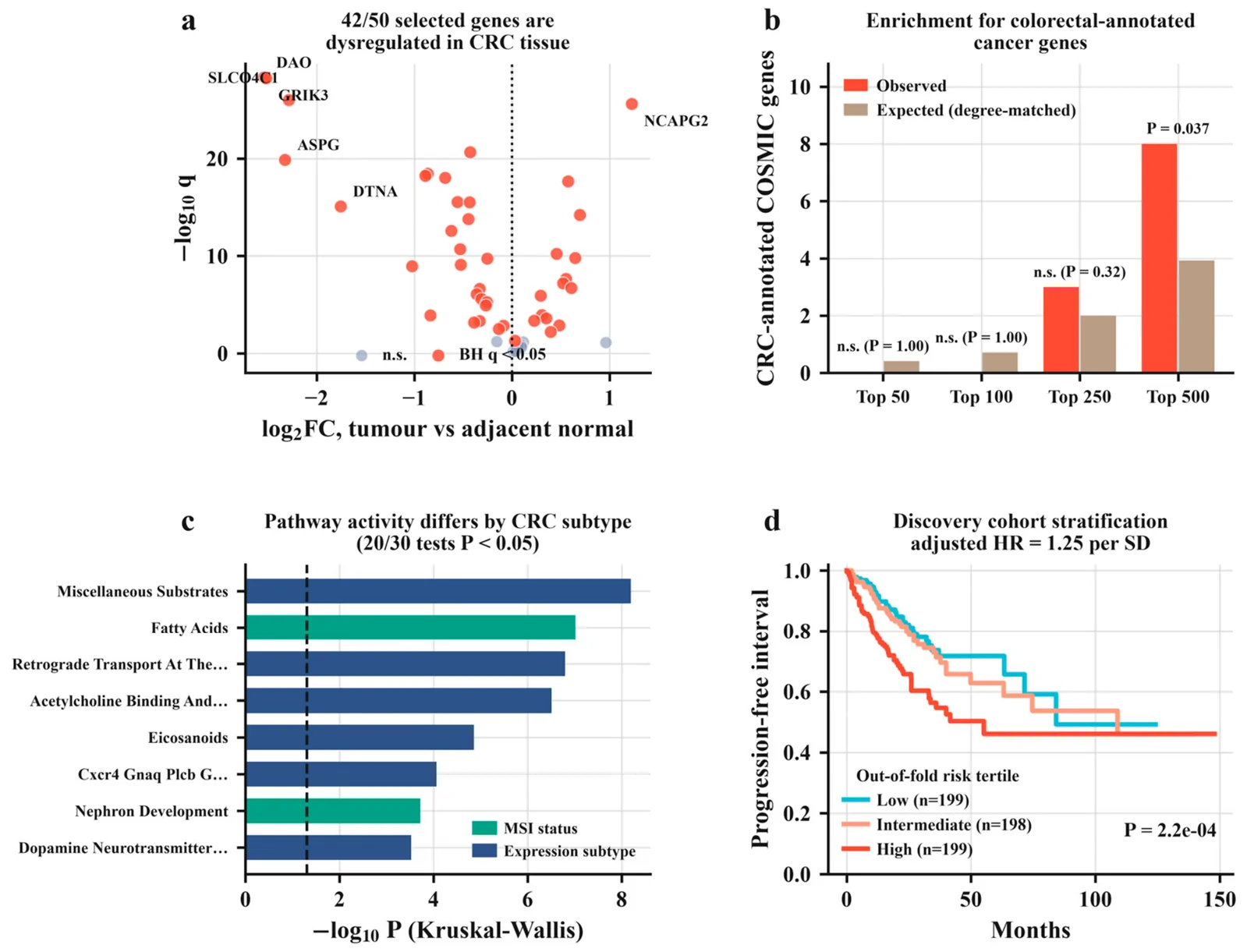
Colorectal cancer relevance of the selected genes and pathways. (**a**) Differential expression of the fifty highest-ranked genes between primary tumours and adjacent normal mucosa; the dotted vertical line marks no fold change. (**b**) Overlap with COSMIC genes annotated for colorectal tumours, against a connectivity-matched null. (**c**) Highest-ranked pathways ordered by how strongly their activity separates microsatellite instability status or expression subtype; both were auxiliary training targets, so this panel is descriptive; the dashed line marks *P* = 0.05, and long pathway names are truncated in the panel and given in full in Table S9. (**d**) Progression-free interval by out-of-fold risk tertile in the discovery cohort.

A prognostic signature is more persuasive when its members are demonstrably part of the disease rather than incidental correlates of outcome. Four analyses addressed that directly. First, 42 of the 50 highest-ranked genes (84%) were differentially expressed between the 647 primary tumour samples (624 patients; Section 2.10) and the 51 adjacent normal samples at a false discovery rate below 5% (Figure 8a, Table 8 and Table S8); restricting the tumours to the 596 patients of the discovery cohort identified the same 42 genes, with log2 fold changes differing by at most 0.04. The largest shifts were reductions in tumour tissue: DAO fell by 2.54 on the log2 scale, GRIK3 by 2.52, ASPG by 2.33 and SLCO4C1 by 2.29, while the condensin subunit NCAPG2 rose by 1.23. These genes are altered in colorectal tumour tissue, which shows relevance to the disease but not specificity relative to other cancers. Second, restricting the COSMIC census to the 86 genes annotated for colorectal, colonic, large-intestinal, bowel or rectal tumours, the top 500 contained 8 such genes against 3.9 expected under a degree-matched null, a two-fold enrichment (*p* = 0.037; Figure 8b, Table 9). This enrichment was confined to the top 500, with none among the top 50 or 100 and no significant excess among the top 250, and it depended on how connectivity was adjusted: across 13 alternative binning schemes, the top-500 permutation *p* ranged from 0.0004 to 0.49 (Table S23). It is therefore weak supporting evidence. The pan-cancer driver lists showed no enrichment under the matched null (Figure S9, Table S10). Third, pathway activity tracked the molecular axes that define colorectal cancer. Of 30 tests across the fifteen highest-ranked pathways, 20 were significant against microsatellite instability status or TCGA expression subtype (Figure 8c, Table S9). Fatty-acid metabolism separated most strongly by instability status, followed by retrograde trans-Golgi transport, eicosanoid metabolism and CXCR4-GNAQ-PLC-beta-calcineurin signalling. Because both variables were auxiliary targets during training, this association is partly built into the fitting and is not independent evidence of disease relevance. Fourth, the score carried no prognostic information outside colorectal cancer. Applied in the same way to TCGA oesophageal carcinoma (184 patients, 87 events) and lung adenocarcinoma (504 patients, 207 events), it reached concordances of 0.513 (95% CI 0.438–0.588) and 0.526 (0.485–0.567), with stage- and age-adjusted hazard ratios of 1.11 (0.85–1.44; *p* = 0.44) and 1.01 (0.88–1.17; *p* = 0.85) (Table S25). The score does not behave as a generic marker of tumour aggressiveness, although two comparator tumour types cannot establish specificity to colorectal cancer. The separation of progression-free interval by out-of-fold risk tertile in the discovery cohort (Section 3.4) is shown in Figure 8d. Annotation of the fifty highest-ranked genes, combining connectivity-adjusted influence, colorectal cancer-gene status and differential expression, is given in Table 8.

**Table 8:** Colorectal annotation of the twenty highest-ranked genes. Fold changes compare 647 primary tumour samples from 624 patients with 51 adjacent normal samples (Section 2.10). The full set of fifty is given in Table S8.

Gene	Importance z	CRC Cancer Gene (COSMIC)	log2FC Tumour vs. Normal	BH q (Tumour vs. Normal)
UBB	8.69	No	−0.53	<1 × 10^−16^
UBC	8.45	No	−0.26	<1 × 10^−16^
RPS27A	7.11	No	+0.30	<1 × 10^−16^
HS6ST3	6.30	No	−0.89	<1 × 10^−16^
CER1	6.23	No	+0.12	6.64 × 10^−2^
CDK6	6.11	No	+0.56	<1 × 10^−16^
UBA52	5.87	No	+0.46	<1 × 10^−16^
CYP4A11	5.86	No	+0.03	4.81 × 10^−2^
IRX2	5.79	No	+0.96	7.32 × 10^−2^
CYP3A43	5.73	No	+0.04	6.20 × 10^−1^
DUOX1	5.55	No	+0.40	6.30 × 10^−3^
COL25A1	5.31	No	−0.56	<1 × 10^−16^
GATM	5.09	No	−1.02	<1 × 10^−16^
DNAH10	5.04	No	+0.52	<1 × 10^−16^
OTUD1	4.95	No	−0.62	<1 × 10^−16^
IL36G	4.95	No	+0.23	4.00 × 10^−4^
DAO	4.94	No	−2.54	<1 × 10^−16^
CHMP4A	4.80	No	+0.09	1.40 × 10^−1^
LIPJ	4.67	No	−0.25	<1 × 10^−16^
GJB2	4.62	No	−0.39	6.00 × 10^−4^

**Table 9:** Enrichment of the ranked genes for COSMIC census genes annotated in colorectal tumours, tested against a null matched on pathway connectivity. Results under alternative connectivity adjustments are given in Table S23.

Top N	Observed	Expected (Degree-Matched)	Fold Enrichment	*p* (Degree-Matched)
50	0	0.41	0.00	1.000
100	0	0.71	0.00	1.000
250	3	2.0	1.50	0.325
500	8	3.92	2.04	0.037

### 3.9. Stage-Specific Effects Remain Uncertain

With stage completed from the TCGA Clinical Data Resource, the stage II subgroup comprised 217 patients with 46 events, and the score did not separate outcomes within it (concordance 0.534; HR 1.06 per SD, *p* = 0.70; Figure S10, Table S11). Age-adjusted hazard ratios per standard deviation were 1.06 (0.46–2.44), 1.06 (0.78–1.46), 1.32 (0.99–1.77) and 1.41 (1.04–1.90) in stages I to IV (Table S21). A formal test found no evidence that the effect differed by stage (score × stage interaction, likelihood-ratio *p* = 0.31 with stage ordinal and 0.73 with stage categorical), and the same was true in GSE39582 (*p* = 0.074; stage II HR 1.15, *p* = 0.26) and GSE17536 (*p* = 0.22). With 7 to 50 events per stage, these analyses can establish neither a uniform effect nor a stage-restricted one. Whether the score is informative in stage II disease, where it would matter most for adjuvant decisions, remains uncertain.

## 4. Discussion

Staging remains the backbone of colorectal cancer prognosis, and any molecular marker proposed alongside it has to answer a narrow question: does it say something the stage does not already say? On 596 patients with 153 progression-free events, an out-of-fold pathway-masked Kolmogorov–Arnold score remained associated with progression after adjustment for stage, age, sex and anatomical site (hazard ratio 1.25 per standard deviation; likelihood-ratio *p* = 0.008), and the frozen network replicated that association in the two larger treatment-naive external cohorts. The gain in discrimination was small: concordance rose from 0.699 to 0.711 in the discovery cohort, with a confidence interval that included no gain, and by 0.007 and 0.006 in the two external cohorts. Three findings should be kept apart here. The score is associated with outcome after adjustment, and adding it significantly improves model fit; an improvement in discrimination, by contrast, has not been statistically established. Neither the association nor the improvement in fit amounts to clinical utility. A hazard ratio of this size shifts individual risk estimates only modestly, and whether the score would change a treatment decision, or improve net benefit at a clinically relevant threshold [53], has not been examined here. Reporting incremental value rather than standalone accuracy is a deliberate choice, and it is the choice the field has been moving towards. Venet and colleagues showed that most random gene sets of sufficient size are significantly associated with breast cancer outcome, simply because expression correlates with proliferation and proliferation correlates with stage [10]. A signature that reaches a respectable concordance on its own may therefore be restating the clinical variables in molecular language. The likelihood-ratio test against a clinical model is a stricter and more informative standard, and it is the one the TRIPOD reporting framework asks for [11]. Our own data show how much such a test depends on the clinical baseline: when stage missing from the GDC record was imputed rather than taken from the curated clinical resource, the clinical model lost about 0.10 in concordance and the apparent gain from the score more than tripled. The precedent for a molecular marker earning a place beside staging in this disease is the Immunoscore, which was validated internationally and shown to carry prognostic information independent of the TNM classification [9]. The score reported here is at a much earlier stage of development: its association with outcome is independent of stage in the discovery cohort and in two of four validation series, with adjusted hazard ratios of 1.24 and 1.34 per standard deviation in those two, and its added discriminative value is small.

The architectural difference is easy to state. A proportional hazards model assigns each gene a single coefficient, so it is committed in advance to the claim that the log-hazard changes linearly with expression. A conventional neural network is not limited in that way: with nonlinear activations, it can represent nonlinear and interacting effects, but those effects are spread across many scalar weights and cannot be read from any one of them. Placing a learnable univariate function on every connection keeps the nonlinearity and makes it inspectable at the level of individual gene-to-pathway edges [12,13]. Several of the strongest edges were not monotonic. In the final ensemble, the CER1 edge contributed little across most of the expression range before rising above the cohort median, and the CYP4A11 edge changed direction across its upper range. These are model-identified nonlinear associations, not biological thresholds. An edge function is an internal component of a multilayer network: a gene acts through several pathways, correlated genes can substitute for one another, and downstream layers can rescale or reverse an edge’s contribution. Across refits, the individual edge curves were not reproducible, and several reversed sign between bootstrap samples, whereas the gene-level partial dependence was stable and mostly monotone: predicted risk rose with CER1 and fell with CYP4A11, with only mild curvature. The interpretable quantity is therefore the gene-level response of the whole network rather than any single edge. A cut-point for a clinical assay would have to be derived and validated directly against outcome, not read from an edge function. Kolmogorov–Arnold layers have been combined with molecular networks before, most directly for pan-cancer classification from multi-omics data [14]. Biologically informed architectures have separately been used to good effect in prostate cancer, where constraining a network to known pathway structure produced a model whose internal nodes could be interrogated [15]. Deep survival models of the DeepSurv and Cox-nnet families brought time-to-event objectives to neural networks but retained dense, scalar-weighted layers [16,17], and multi-omics survival models have followed a similar pattern [18]. What has not been done is to put function-valued edges on a sparse, biologically sanctioned topology and fit them to a time-to-event objective, which is the combination examined here.

The genes the model prioritised are not the genes that dominate the colorectal cancer literature. Once the null was matched on pathway connectivity, overlap with pan-cancer driver lists was indistinguishable from chance, whereas the same comparison against an unmatched null produced an apparent four- to five-fold enrichment. Curated driver catalogues are themselves enriched for heavily studied and heavily connected genes, so any ranking with a connectivity bias will clear the naive threshold, and enrichment statistics computed without that control are difficult to interpret. Restricting the census to genes annotated in colorectal tumours gave a two-fold enrichment among the top 500 genes, but not among the top 50 to 250, and its significance depended on how connectivity was adjusted, so we treat it as weak supporting evidence. The more direct observations are that 42 of the 50 highest-ranked genes are differentially expressed between tumour and adjacent normal mucosa, and that the score carried no prognostic information in oesophageal carcinoma or lung adenocarcinoma. Together they point to a signature associated with colorectal cancer biology rather than a generic marker of aggressiveness. They do not establish specificity: tumour-versus-normal differences show relevance to colorectal tissue, not uniqueness relative to other cancers; two comparator tumour types are few; and the pathway-by-subtype analysis is not independent of the fitting, because microsatellite instability and expression subtype were auxiliary training targets. With those caveats, the leading pathways are biologically plausible. Fatty-acid metabolism separated microsatellite instability classes most strongly, and lipid reprogramming is increasingly recognised as a targetable metabolic dependency in colorectal tumours [54]. CXCR4 signalling, also highly ranked, governs invasion and metastatic homing in this disease and is under active therapeutic investigation [55]. Aquaporin-mediated transport, the single highest-ranked pathway after adjustment for set size, has been linked to proliferation and migration across several tumour types [56]. Stromal content is a well-established determinant of poor outcome in colorectal cancer and plausibly underlies part of what the model detects [57], as does eicosanoid metabolism downstream of arachidonic acid [58]. Read together, these are programmes of the tumour microenvironment and of metabolic adaptation [59], not of the mutational events that initiate the disease.

A 24-configuration search moved cross-validated concordance across a band of roughly 0.015, and the configuration chosen varied between the outer folds of the nested analysis without changing the estimate. A flat surface of that kind shows that these hyperparameters matter little at this sample size, but it does not by itself identify what limits performance. A learning curve over 49 to 122 training events was also flat, so within the range we could examine, neither tuning nor event count explains the modest discrimination; larger cohorts would be needed to tell whether more data, a different architecture or a limited prognostic signal in bulk expression sets the ceiling. Independent benchmarking has found that carefully tuned baselines match elaborate representations on bulk expression survival tasks [19]. The practical consequence is that comparisons of standalone concordance between architectures on cohorts of this size carry little information. Two models separated by 0.01 concordance and overlapping fold distributions are not distinguishable, and naive fold-level tests overstate the evidence, because cross-validation folds share most of their training data: after correction for that dependence, even the advantage of PathKAN over elastic-net Cox was no longer significant. Reporting the paired fold-level comparison, the corrected test and the incremental test makes that visible, and it is the reason this study frames its result in terms of what the score adds rather than where it ranks.

Two design choices did most of the work in getting a model fitted on sequencing counts to function on microarrays. The first was to train and validate on the same representation: within-sample gene ranks mapped to normal quantiles, so only the ordering of genes inside a patient crosses between technologies. The second was to restrict the input space in advance to transcripts measured on every platform, which meant the frozen model never encountered a missing input and never had to impute one. A third element also mattered: per-gene centring and scaling within each external cohort. With those parameters fixed at the discovery values, the association was preserved in GSE17536 but attenuated in GSE39582, plausibly because probe-specific effects shift a gene’s typical within-sample rank on an array relative to sequencing. What transfers is therefore a set of frozen weights together with an outcome-blind, cohort-level standardisation, and the transportability reported here depends partly on that preprocessing step rather than on the weights alone. Each external cohort supplied its own per-gene mean and standard deviation, estimated from tens to hundreds of samples, whereas a patient seen in practice arrives alone and offers no cohort from which to estimate them. Direct application of the score to individual future patients is therefore not supported by the present results. It would require a fixed external standardisation reference, such as a panel of calibrator samples or per-gene reference distributions derived from a sufficiently large series on the intended assay platform, and the adequacy of that reference would itself have to be tested against outcome before use. The attenuation seen in GSE39582 under discovery-fixed scaling shows that the choice of reference is not a formality. Colon and rectal tumours were analysed together throughout, following the molecular equivalence established by the integrated TCGA analysis [7], and reflected in the consensus subtypes that span both sites [6]. Progression-free interval did not differ between them in this cohort, and anatomical site was carried as a covariate and used to stratify every split rather than being assumed away.

Three lines of work would move this from a replicated association towards something usable. The first is prospective evaluation on sequencing data, since the validation cohorts here are archival and array-based; the rank representation was built precisely so that a sequencing-based test could reuse the same frozen model, provided a fixed external standardisation reference is first established and validated, because the cohort-level centring and scaling used here cannot be reproduced for a single patient. The second is a treatment-aware analysis. The score was fitted to prognosis, and prognosis and treatment benefit are different quantities: the cohort in which a marker would change management is the one where adjuvant chemotherapy is genuinely optional [4], and answering that question requires randomised data rather than observational follow-up. The third is assay reduction. Influence is resolved here at the level of individual gene-pathway connections, so the same framework can identify a compact panel whose members and expression ranges would then need validation in their own right. The wider point concerns how prognostic models are reported. Prognostic biomarker development in colorectal cancer has produced many signatures, and few adopted tests [5], and the gap is rarely one of algorithmic sophistication. Stating what a model adds to the variables already in the clinic, against a complete clinical baseline, showing the shape of each contribution rather than a ranked list, and testing biological claims against a null that controls for how well studied a gene is would narrow that gap more reliably than another increment of concordance.

This study has further limitations. The discovery cohort carries 153 events, while the network fits 127,985 edge functions to 9749 inputs; with far fewer events than parameters, the final network reaches a concordance of 0.98 on its own training data, so only out-of-fold and external estimates are informative, and the cohort is well below the size recommended for developing a clinical prediction model [60]. Cross-validation estimates are correlated across folds and repetitions; corrected tests and patient-level bootstrap intervals reduce, but do not remove, that problem. External replication was positive in two of four cohorts: the other two were too small to be informative, and one was confounded by preoperative treatment, and the external endpoints (overall, disease-free and disease-specific survival) differ from the progression-free interval used for training. The external results also depend partly on outcome-blind, cohort-level centring and scaling of the inputs: with scaling fixed at the discovery values, the association was attenuated in GSE39582 (Table S13), so the transportability shown here is that of the frozen weights together with a cohort-level preprocessing step, not of a score that can yet be computed for an individual patient without a fixed external standardisation reference. Clinical covariates were incomplete or differently defined across cohorts, so incremental value could be assessed externally only with the variables each cohort recorded. Stage was missing from the GDC record for 19% of the discovery cohort, and the curated stage that replaced it may itself contain errors. Interpretation rests on internal edge functions that are not causal gene effects and were not individually reproducible across refits, and on a gene ranking whose lower-degree members varied between refits and with the connectivity adjustment. Finally, calibration, net benefit and treatment interaction were not assessed, so nothing here establishes clinical utility.

## 5. Conclusions

A pathway-masked Kolmogorov–Arnold survival model yields a transcriptomic score that is associated with progression in colorectal cancer independently of stage, age, sex and tumour site (adjusted hazard ratio 1.25 per standard deviation; likelihood-ratio *p* = 0.008). Adding the score to these variables improved model fit, but the gain in concordance was small (0.699 to 0.711), and its 95% confidence interval included zero, so an improvement in discrimination has not been statistically established. Neither association nor improved fit establishes clinical utility, which remains untested. With network weights frozen and inputs standardised within each cohort, the score remained independently prognostic in two of four external cohorts, the two larger treatment-naive series; the two smaller cohorts were inconclusive. This transportability depends partly on outcome-blind, cohort-level standardisation of the inputs, and direct application to individual future patients will require a fixed external standardisation reference to be established and validated. Because every retained connection carries a function rather than a coefficient, the fitted model exposes the shape of each learned transformation; individual edge functions were not reproducible across refits, whereas gene-level partial-dependence curves were stable, smooth and mostly monotone. These are model-identified associations that require direct validation before any biological or clinical threshold is inferred. Most prioritised genes are differentially expressed between tumour and normal mucosa, and the score is not prognostic in oesophageal carcinoma or lung adenocarcinoma, which supports relevance to colorectal cancer biology without establishing specificity. Pathway-constrained, interpretable models are a reasonable route to candidate prognostic biomarkers, provided their added value is judged against a complete clinical baseline.

## Data Availability

All data analysed are publicly available. Expression and clinical data for TCGA-COAD and TCGA-READ were obtained from the Genomic Data Commons (release 45.0). Validation cohorts are available from the Gene Expression Omnibus under accessions GSE39582, GSE17536, GSE33113 and GSE87211. The code is publicly available at https://github.com/kallemailem/crc-pathkan.git, accessed on 10 October 2026.

## Supplementary Materials

The following supporting information can be downloaded at: https://www.mdpi.com/article/10.3390/life16101743/s1, Figure S1: Alignment of the two variance-stabilised expression blocks; Figure S2: Endpoint comparison in the discovery cohort; Figure S3: Structure of the prior-knowledge network; Figure S4: Inner cross-validated concordance across the 24-configuration hyperparameter sweep, shown separately for each searched parameter; Figure S5: Concordance of every model in each cross-validation fold, on identical splits; Figure S6: Expression distributions of the discovery cohort and the four validation series on their native scales; Figure S7: The post-chemoradiation rectal cohort; Figure S8: Distribution of the frozen risk score in patients with and without a recorded event, in each validation cohort; Figure S9: Overlap of the ranked genes with COSMIC, OncoKB and their union, compared with a random null and with a null matched on pathway connectivity; Figure S10: Stage II subgroup of the discovery cohort; Figure S11: Learning curve; Figure S12: Stability of the interpretability readout across the ten cross-validation networks, the five final-ensemble seeds and 20 bootstrap refits; Table S1: Provenance of the four independent validation series, recorded at download; Table S2: Full hyperparameter sweep: 24 configurations evaluated under inner three-fold cross-validation of the discovery cohort; Table S3: Concordance of every model in each of the ten cross-validation folds, computed on identical splits; Table S4: Per-fold concordance for the clinical model, the molecular score alone, and the two combined; Table S5: Gene-level influence with connectivity adjustment, for the 1000 highest-ranked genes; Table S6: Pathway-level influence with adjustment for set size, for all 1792 retained pathways; Table S7: The 2000 most influential individual gene-to-pathway connections; Table S8: Differential expression of the fifty highest-ranked genes between 647 primary tumour samples (all primary-tumour aliquots retrieved, from 624 patients) and 51 adjacent normal samples; Table S9: Activity of the fifteen highest-ranked pathways across microsatellite instability status and TCGA expression subtype; Table S10: Overlap of the ranked genes with pan-cancer driver lists under a random null and a connectivity-matched null; Table S11: Performance of the score within the stage II subgroup of the discovery cohort; Table S12: Sensitivity of the incremental-value analysis to the coding of AJCC stage; Table S13: External cohorts: input scaling and incremental value; Table S14: Lookup-table versus exact Cox–de Boor evaluation of the edge splines, for the ten cross-validation networks on their own test folds and for the final ensemble; Table S15: Fully nested cross-validation; Table S16: Inference that accounts for the dependence between cross-validation folds; Table S17: Fold-mean concordance of each model with naive and dependence-corrected 95% t intervals (Nadeau–Bengio correction); Table S18: Proportional-hazards diagnostics: scaled Schoenfeld residual tests (Grambsch–Therneau) for the adjusted clinical + PathKAN Cox model in each cohort, with rank and Kaplan–Meier time transforms; Table S19: PathKAN hazard ratio with the baseline hazard stratified by stage, which removes any proportionality assumption for stage; Table S20: Landmark split of follow-up at 24 months: adjusted hazard ratio of the score for events up to 24 months and, among patients event-free and under follow-up at 24 months, for later events; Table S21: Stage-specific hazard ratios of the PathKAN score (adjusted for age) and likelihood-ratio tests of a score × stage interaction added to the adjusted model; Table S22: Specification of the connectivity adjustment: the 12 equal-count bins of log2 pathway degree used for the reported gene ranking (ties in degree broken alphabetically); Table S23: Sensitivity of the gene ranking and of the colorectal COSMIC enrichment (top 500) to the connectivity adjustment; Table S24: Stability of the fifteen reported top genes across the ten cross-validation networks, the five final-ensemble seeds and 20 bootstrap refits; Table S25: The frozen PathKAN ensemble applied to two non-colorectal TCGA cohorts processed through the same GDC pipeline (primary tumours, one sample per patient), with the TCGA-CDR progression-free interval as outcome; Table S26: Learning curve over the ten outer folds (two seeds per PathKAN fit); and a workbook containing the complete gene, pathway and edge influence tables.

## References

[1] Bray F., Laversanne M., Sung H., Ferlay J., Siegel R.L., Soerjomataram I., Jemal A. (2024). Global cancer statistics 2022: GLOBOCAN estimates of incidence and mortality worldwide for 36 cancers in 185 countries. CA Cancer J. Clin..

[2] Siegel R.L., Wagle N.S., Cercek A., Smith R.A., Jemal A. (2023). Colorectal cancer statistics, 2023. CA Cancer J. Clin..

[3] Dekker E., Tanis P.J., Vleugels J.L.A., Kasi P.M., Wallace M.B. (2019). Colorectal cancer. Lancet.

[4] Argiles G., Tabernero J., Labianca R., Hochhauser D., Salazar R., Iveson T., Laurent-Puig P., Quirke P., Yoshino T., Taieb J. (2020). Localised colon cancer: ESMO Clinical Practice Guidelines for diagnosis, treatment and follow-up. Ann. Oncol..

[5] Manne U., Kumar Putcha B.D., Samuel T., Srivastava S. (2017). Prognostic and Predictive Biomarkers for Colorectal Cancer. Biomarkers in Cancer Screening and Early Detection.

[6] Guinney J., Dienstmann R., Wang X., de Reynies A., Schlicker A., Soneson C., Marisa L., Roepman P., Nyamundanda G., Angelino P. (2015). The consensus molecular subtypes of colorectal cancer. Nat. Med..

[7] The Cancer Genome Atlas Network (2012). Comprehensive molecular characterization of human colon and rectal cancer. Nature.

[8] Parine N.R., Varsha R.S., Alanazi M.S. (2016). Microsatellite Instability in Colorectal Cancer. Microsatellite Markers.

[9] Pages F., Mlecnik B., Marliot F., Bindea G., Ou F.S., Bifulco C., Lugli A., Zlobec I., Rau T.T., Berger M.D. (2018). International validation of the consensus Immunoscore for the classification of colon cancer: A prognostic and accuracy study. Lancet.

[10] Venet D., Dumont J.E., Detours V. (2011). Most random gene expression signatures are significantly associated with breast cancer outcome. PLoS Comput. Biol..

[11] Collins G.S., Reitsma J.B., Altman D.G., Moons K.G. (2015). Transparent reporting of a multivariable prediction model for individual prognosis or diagnosis (TRIPOD): The TRIPOD statement. BMJ.

[12] Martinez-Heredia A.M., Ortiz A. (2026). Kolmogorov-Arnold Networks for Sensor Data Processing: A Comprehensive Survey of Architectures, Applications, and Open Challenges. Sensors.

[13] Schmidt-Hieber J. (2021). The Kolmogorov-Arnold representation theorem revisited. Neural Netw..

[14] Alharbi F., Budhiraja N., Vakanski A., Zhang B., Elbashir M.K., Guduru H., Mohammed M. (2025). Interpretable graph Kolmogorov-Arnold networks for multi-cancer classification and biomarker identification using multi-omics data. Sci. Rep..

[15] Elmarakeby H.A., Hwang J., Arafeh R., Crowdis J., Gang S., Liu D., AlDubayan S.H., Salari K., Kregel S., Richter C. (2021). Biologically informed deep neural network for prostate cancer discovery. Nature.

[16] Katzman J.L., Shaham U., Cloninger A., Bates J., Jiang T., Kluger Y. (2018). DeepSurv: Personalized treatment recommender system using a Cox proportional hazards deep neural network. BMC Med. Res. Methodol..

[17] Ching T., Zhu X., Garmire L.X. (2018). Cox-nnet: An artificial neural network method for prognosis prediction of high-throughput omics data. PLoS Comput. Biol..

[18] Chaudhary K., Poirion O.B., Lu L., Garmire L.X. (2018). Deep Learning-Based Multi-Omics Integration Robustly Predicts Survival in Liver Cancer. Clin. Cancer Res..

[19] Gross B., Dauvin A., Cabeli V., Kmetzsch V., El Khoury J., Dissez G., Ouardini K., Grouard S., Davi A., Loeb R. (2024). Robust evaluation of deep learning-based representation methods for survival and gene essentiality prediction on bulk RNA-seq data. Sci. Rep..

[20] Cox D.R. (1972). Regression Models and Life-Tables. J. R. Stat. Soc. Ser. B Stat. Methodol..

[21] Simon N., Friedman J., Hastie T., Tibshirani R. (2011). Regularization Paths for Cox’s Proportional Hazards Model via Coordinate Descent. J. Stat. Softw..

[22] Colaprico A., Silva T.C., Olsen C., Garofano L., Cava C., Garolini D., Sabedot T.S., Malta T.M., Pagnotta S.M., Castiglioni I. (2016). TCGAbiolinks: An R/Bioconductor package for integrative analysis of TCGA data. Nucleic Acids Res..

[23] Love M.I., Huber W., Anders S. (2014). Moderated estimation of fold change and dispersion for RNA-seq data with DESeq2. Genome Biol..

[24] Seal R.L., Braschi B., Gray K., Jones T.E.M., Tweedie S., Haim-Vilmovsky L., Bruford E.A. (2023). Genenames.org: The HGNC resources in 2023. Nucleic Acids Res..

[25] Liu J., Lichtenberg T., Hoadley K.A., Poisson L.M., Lazar A.J., Cherniack A.D., Kovatich A.J., Benz C.C., Levine D.A., Lee A.V. (2018). An Integrated TCGA Pan-Cancer Clinical Data Resource to Drive High-Quality Survival Outcome Analytics. Cell.

[26] Barrett T., Wilhite S.E., Ledoux P., Evangelista C., Kim I.F., Tomashevsky M., Marshall K.A., Phillippy K.H., Sherman P.M., Holko M. (2013). NCBI GEO: Archive for functional genomics data sets—Update. Nucleic Acids Res..

[27] Marisa L., de Reynies A., Duval A., Selves J., Gaub M.P., Vescovo L., Etienne-Grimaldi M.C., Schiappa R., Guenot D., Ayadi M. (2013). Gene expression classification of colon cancer into molecular subtypes: Characterization, validation, and prognostic value. PLoS Med..

[28] Smith J.J., Deane N.G., Wu F., Merchant N.B., Zhang B., Jiang A., Lu P., Johnson J.C., Schmidt C., Bailey C.E. (2010). Experimentally derived metastasis gene expression profile predicts recurrence and death in patients with colon cancer. Gastroenterology.

[29] de Sousa E.M.F., Colak S., Buikhuisen J., Koster J., Cameron K., de Jong J.H., Tuynman J.B., Prasetyanti P.R., Fessler E., van den Bergh S.P. (2011). Methylation of cancer-stem-cell-associated Wnt target genes predicts poor prognosis in colorectal cancer patients. Cell Stem Cell.

[30] Hu Y., Gaedcke J., Emons G., Beissbarth T., Grade M., Jo P., Yeager M., Chanock S.J., Wolff H., Camps J. (2018). Colorectal cancer susceptibility loci as predictive markers of rectal cancer prognosis after surgery. Genes Chromosomes Cancer.

[31] Subramanian A., Tamayo P., Mootha V.K., Mukherjee S., Ebert B.L., Gillette M.A., Paulovich A., Pomeroy S.L., Golub T.R., Lander E.S. (2005). Gene set enrichment analysis: A knowledge-based approach for interpreting genome-wide expression profiles. Proc. Natl. Acad. Sci. USA.

[32] Liberzon A., Birger C., Thorvaldsdottir H., Ghandi M., Mesirov J.P., Tamayo P. (2015). The Molecular Signatures Database (MSigDB) hallmark gene set collection. Cell Syst..

[33] Milacic M., Beavers D., Conley P., Gong C., Gillespie M., Griss J., Haw R., Jassal B., Matthews L., May B. (2024). The Reactome Pathway Knowledgebase 2024. Nucleic Acids Res..

[34] Kanehisa M., Furumichi M., Sato Y., Ishiguro-Watanabe M., Tanabe M. (2021). KEGG: Integrating viruses and cellular organisms. Nucleic Acids Res..

[35] Oughtred R., Rust J., Chang C., Breitkreutz B.J., Stark C., Willems A., Boucher L., Leung G., Kolas N., Zhang F. (2021). The BioGRID database: A comprehensive biomedical resource of curated protein, genetic, and chemical interactions. Protein Sci..

[36] Sondka Z., Dhir N.B., Carvalho-Silva D., Jupe S., Madhumita, McLaren K., Starkey M., Ward S., Wilding J., Ahmed M. (2024). COSMIC: A curated database of somatic variants and clinical data for cancer. Nucleic Acids Res..

[37] Futreal P.A., Coin L., Marshall M., Down T., Hubbard T., Wooster R., Rahman N., Stratton M.R. (2004). A census of human cancer genes. Nat. Rev. Cancer.

[38] Chakravarty D., Gao J., Phillips S.M., Kundra R., Zhang H., Wang J., Rudolph J.E., Yaeger R., Soumerai T., Nissan M.H. (2017). OncoKB: A Precision Oncology Knowledge Base. JCO Precis. Oncol..

[39] LeCun Y., Bengio Y., Hinton G. (2015). Deep learning. Nature.

[40] Breslow N. (1974). Covariance analysis of censored survival data. Biometrics.

[41] Gu X., Zhang Z., Jiang Y., Luo T., Zhang R., Cui S., Li Z. (2024). Hierarchical Weight Averaging for Deep Neural Networks. IEEE Trans. Neural Netw. Learn. Syst..

[42] Nakamura K., Hong B.-W. (2019). Adaptive Weight Decay for Deep Neural Networks. IEEE Access.

[43] Varma S., Simon R. (2006). Bias in error estimation when using cross-validation for model selection. BMC Bioinform..

[44] Ishwaran H., Kogalur U.B., Blackstone E.H., Lauer M.S. (2008). Random survival forests. Ann. Appl. Stat..

[45] Harrell F.E., Califf R.M., Pryor D.B., Lee K.L., Rosati R.A. (1982). Evaluating the yield of medical tests. JAMA.

[46] Harrell F.E., Lee K.L., Mark D.B. (1996). Multivariable prognostic models: Issues in developing models, evaluating assumptions and adequacy, and measuring and reducing errors. Stat. Med..

[47] Nadeau C., Bengio Y. (2003). Inference for the generalization error. Mach. Learn..

[48] Grambsch P.M., Therneau T.M. (1994). Proportional hazards tests and diagnostics based on weighted residuals. Biometrika.

[49] Benjamini Y., Hochberg Y. (1995). Controlling the False Discovery Rate: A Practical and Powerful Approach to Multiple Testing. J. R. Stat. Soc. Ser. B Stat. Methodol..

[50] Harris C.R., Millman K.J., van der Walt S.J., Gommers R., Virtanen P., Cournapeau D., Wieser E., Taylor J., Berg S., Smith N.J. (2020). Array programming with NumPy. Nature.

[51] Virtanen P., Gommers R., Oliphant T.E., Haberland M., Reddy T., Cournapeau D., Burovski E., Peterson P., Weckesser W., Bright J. (2020). SciPy 1.0: Fundamental algorithms for scientific computing in Python. Nat. Methods.

[52] Davidson-Pilon C. (2019). lifelines: Survival analysis in Python. J. Open Source Softw..

[53] Vickers A.J., Elkin E.B. (2006). Decision curve analysis: A novel method for evaluating prediction models. Med. Decis. Mak..

[54] Zaytseva Y. (2021). Lipid Metabolism as a Targetable Metabolic Vulnerability in Colorectal Cancer. Cancers.

[55] Khare T., Bissonnette M., Khare S. (2021). CXCL12-CXCR4/CXCR7 Axis in Colorectal Cancer: Therapeutic Target in Preclinical and Clinical Studies. Int. J. Mol. Sci..

[56] Moon C.S., Moon D., Kang S.K. (2022). Aquaporins in Cancer Biology. Front. Oncol..

[57] Calon A., Lonardo E., Berenguer-Llergo A., Espinet E., Hernando-Momblona X., Iglesias M., Sevillano M., Palomo-Ponce S., Tauriello D.V., Byrom D. (2015). Stromal gene expression defines poor-prognosis subtypes in colorectal cancer. Nat. Genet..

[58] Xu C., Gu L., Hu L., Jiang C., Li Q., Sun L., Zhou H., Liu Y., Xue H., Li J. (2023). FADS1-arachidonic acid axis enhances arachidonic acid metabolism by altering intestinal microecology in colorectal cancer. Nat. Commun..

[59] Qiu M., Lan C., Lin M., Ma H. (2026). Key immune cells in the tumor immune microenvironment of colorectal cancer: Roles and research advances (Review). Oncol. Rep..

[60] Riley R.D., Ensor J., Snell K.I.E., Harrell F.E., Martin G.P., Reitsma J.B., Moons K.G.M., Collins G., van Smeden M. (2020). Calculating the sample size required for developing a clinical prediction model. BMJ.

