# Supplementary material for: Pathway-Masked Kolmogorov–Arnold Networks Yield an Interpretable Transcriptomic Score Associated with Colorectal Cancer Progression Independently of Clinical Stage
Source: Life (Basel). 2026 Oct 17;16(10):1743. doi: 10.3390/life16101743 (PMC13706471; doi:10.3390/life16101743)
Supplement: Supplementary file 1 [file life-16-01743-s001.zip › life-4523181-supplementary.pdf]

Supplementary Materials

Pathway-Masked Kolmogorov–Arnold Networks Yield an Interpretable Transcriptomic Score Associated with Colorectal Cancer Progression Independently of Clinical Stage

Khaled S. Allemailem a,*, Hajed Obaid Alharbi a, Faris Alrumaihi a, Ahmad Almatroudi a

a Department of Medical Laboratories, College of Applied Medical Sciences, Qassim University, Buraydah 51452, Saudi Arabia

* Corresponding author. Department of Medical Laboratories, College of Applied Medical Sciences, Qassim University, Buraydah 51452, Saudi Arabia.

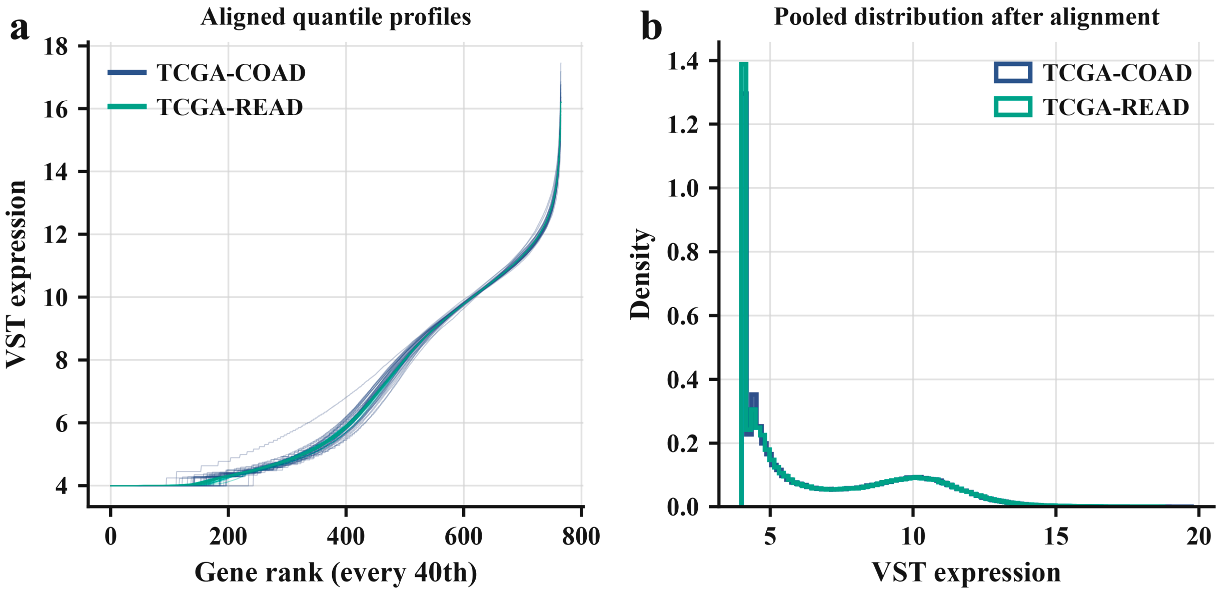

Figure S1. Alignment of the two variance-stabilised expression blocks. (a) Sorted expression profiles for 40 randomly chosen samples from each project. (b) Pooled expression distribution after alignment.

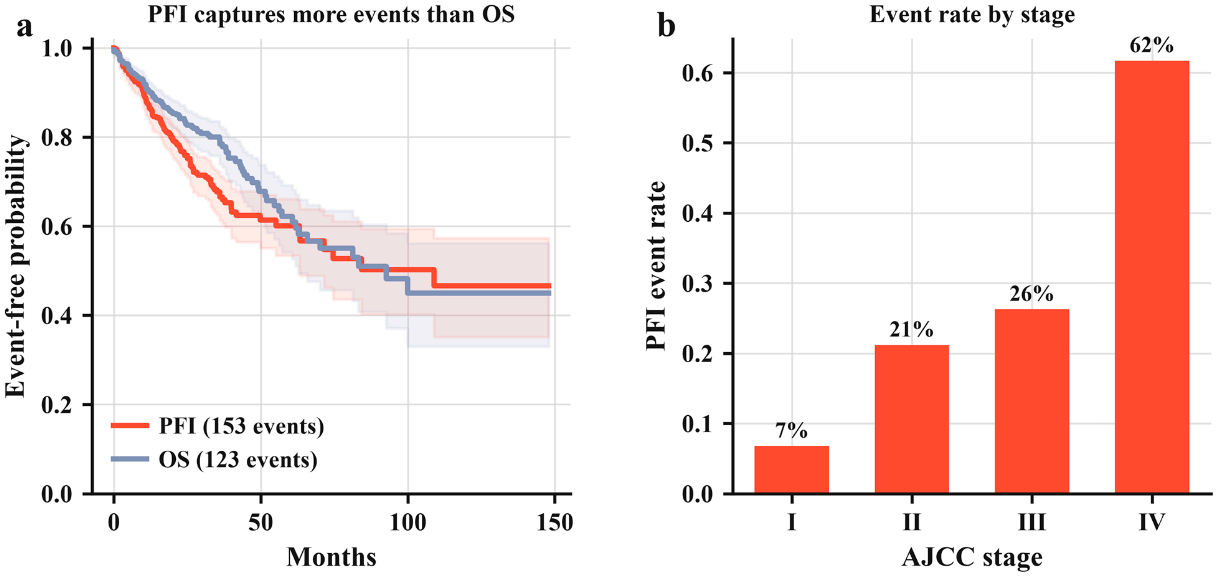

Figure S2. Endpoint comparison in the discovery cohort. (a) Progression-free interval and overall survival in the same patients. (b) Progression-free event rate by stage.

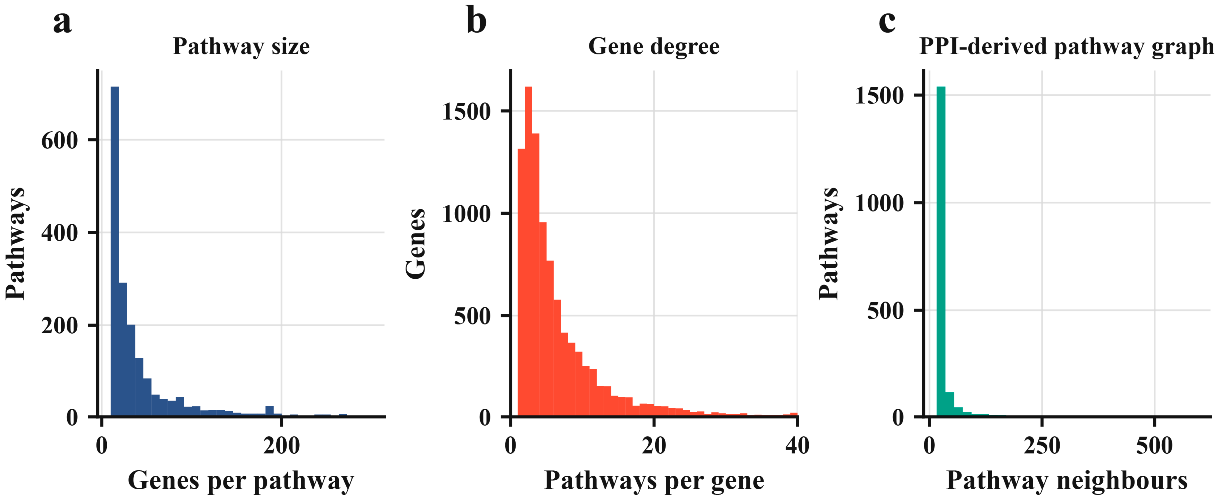

Figure S3. Structure of the prior-knowledge network. (a) Pathway sizes. (b) Number of pathways each gene belongs to. (c) Neighbour counts in the interaction-derived pathway graph.

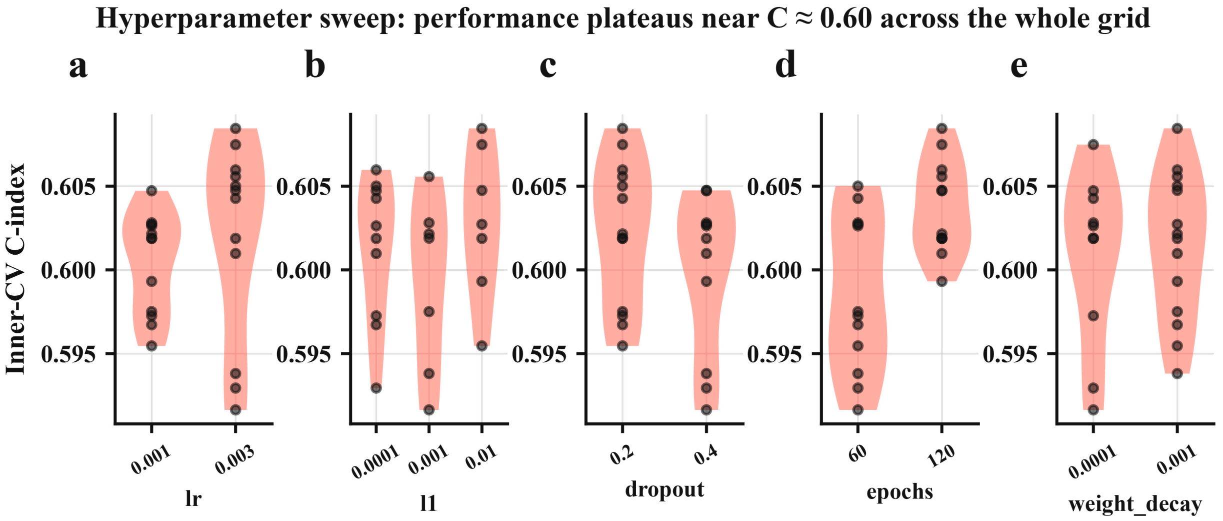

Figure S4. Inner cross-validated concordance across the 24-configuration hyperparameter sweep, shown separately for each searched parameter.

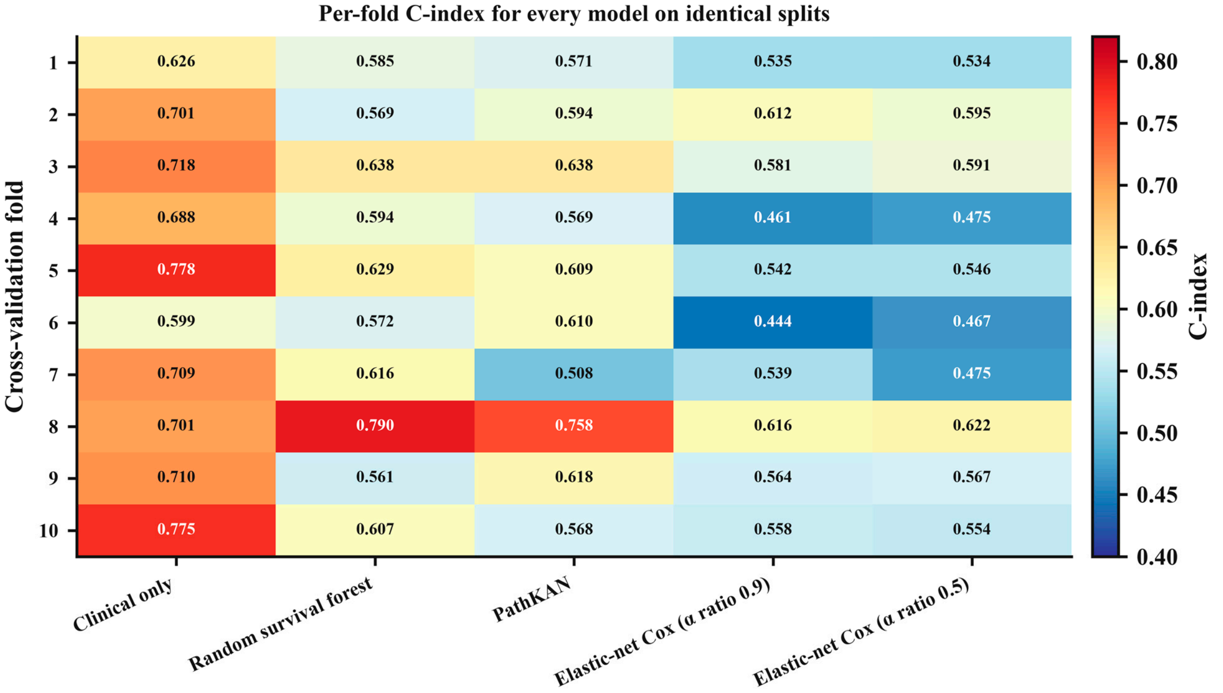

Figure S5. Concordance of every model in each cross-validation fold, on identical splits.

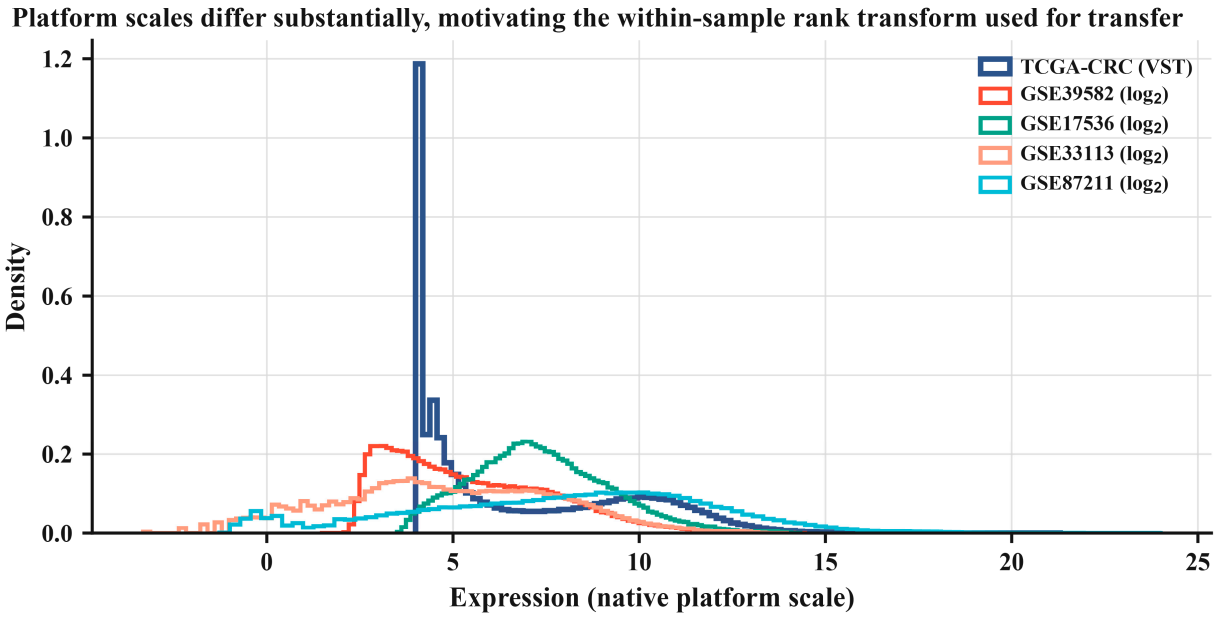

Figure S6. Expression distributions of the discovery cohort and the four validation series on their native scales.

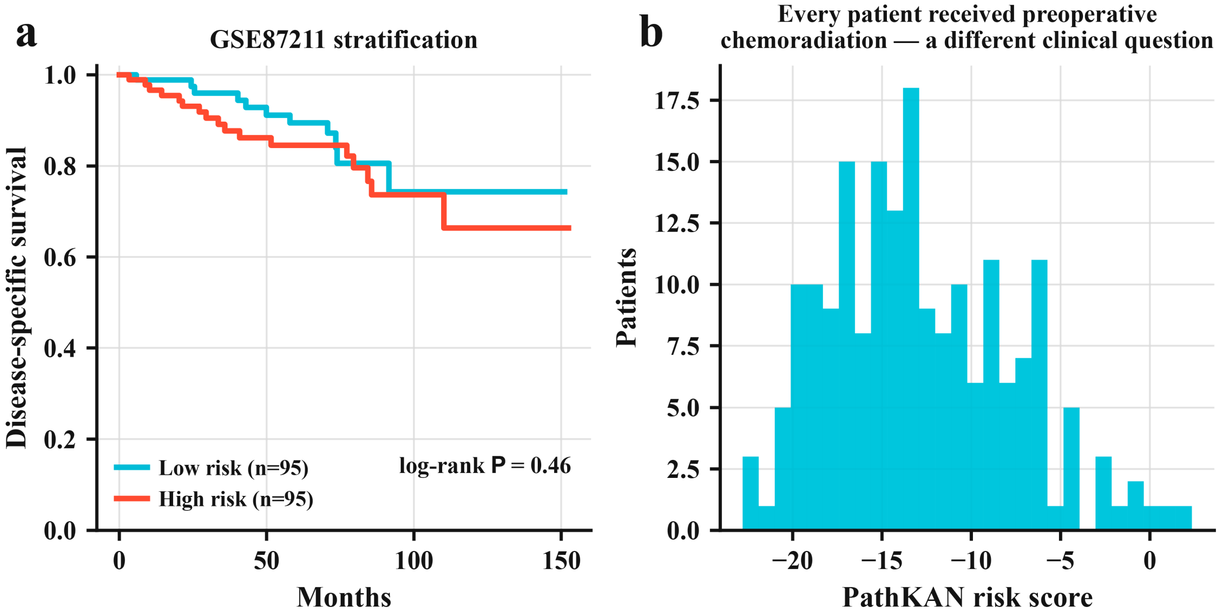

Figure S7. The post-chemoradiation rectal cohort. (a) Stratification by the frozen score. (b) Distribution of the risk score.

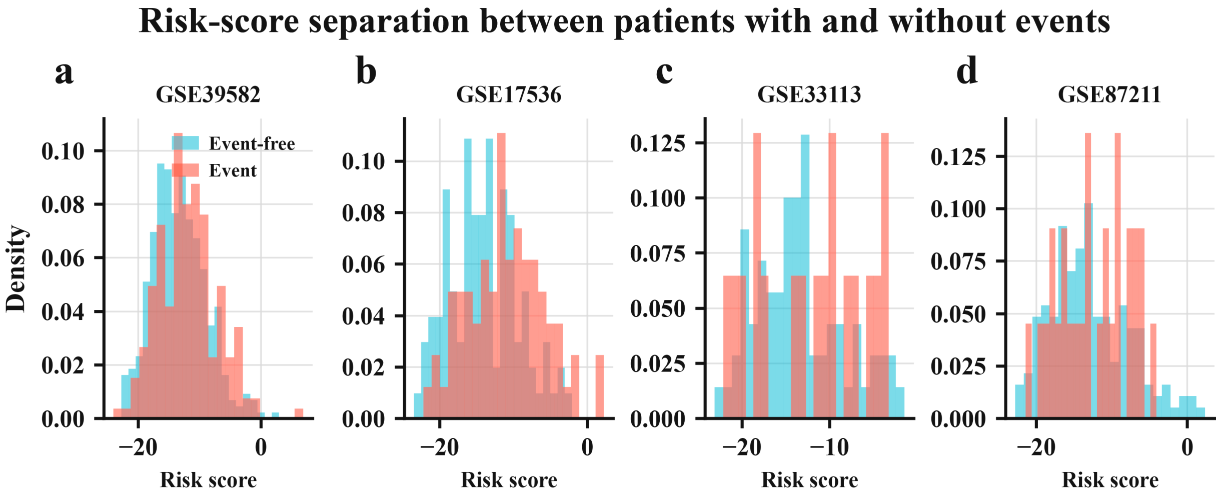

Figure S8. Distribution of the frozen risk score in patients with and without a recorded event, in each validation cohort.

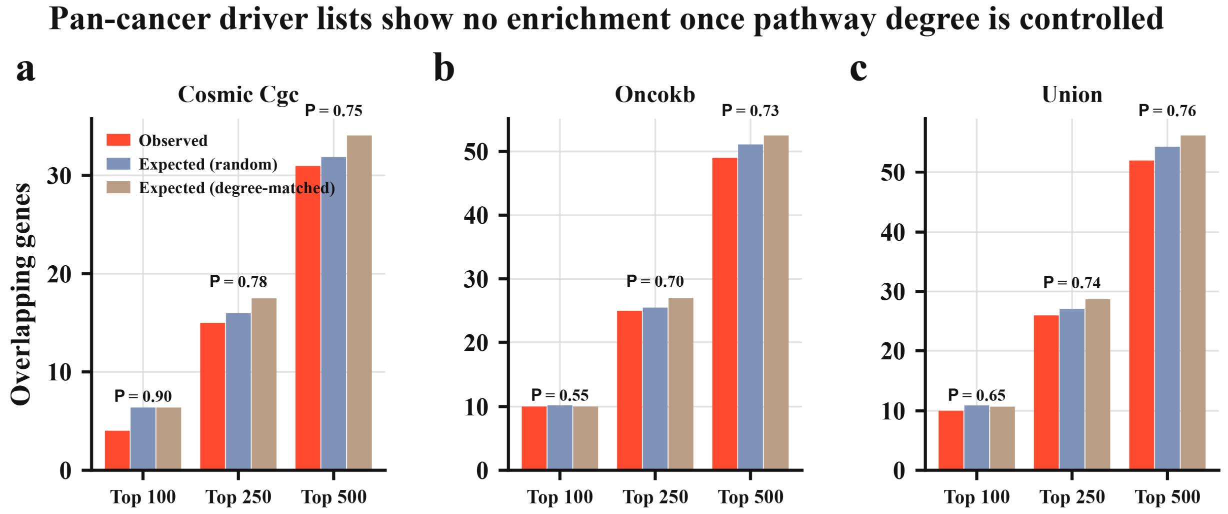

Figure S9. Overlap of the ranked genes with COSMIC, OncoKB and their union, compared with a random null and with a null matched on pathway connectivity.

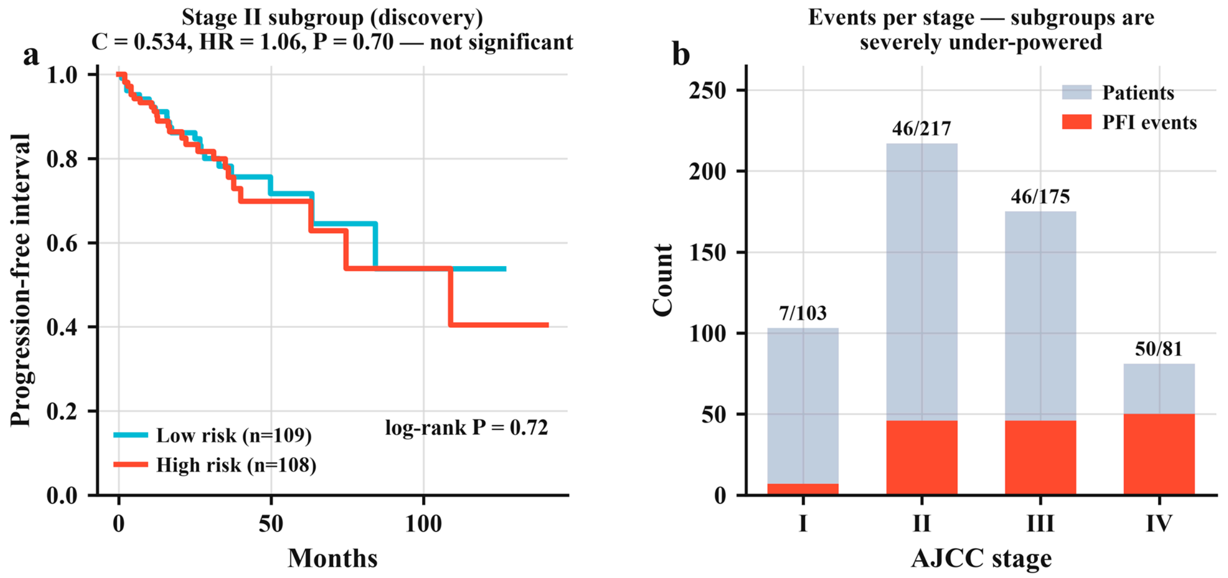

Figure S10. Stage II subgroup of the discovery cohort. (a) Stratification by the score within stage II. (b) Patients and events by stage. Stage completed from the TCGA Clinical Data Resource.

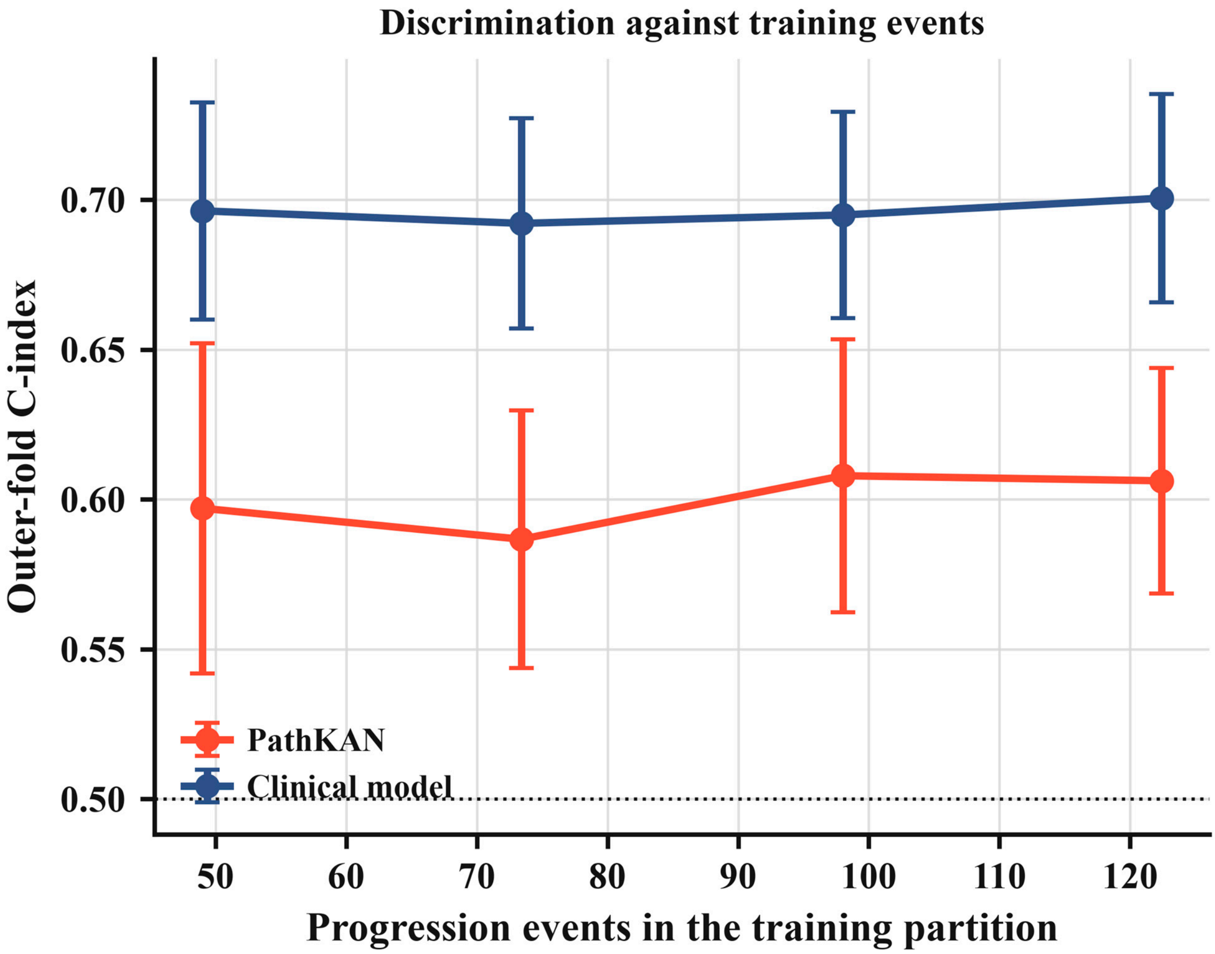

Figure S11. Learning curve. The training partition of every outer cross-validation fold was subsampled, stratified on event, to 40%, 60%, 80% and 100%; PathKAN (final configuration) and the clinical Cox model were refitted and scored on the untouched outer test fold. Points show means over the ten folds with 95% confidence intervals.

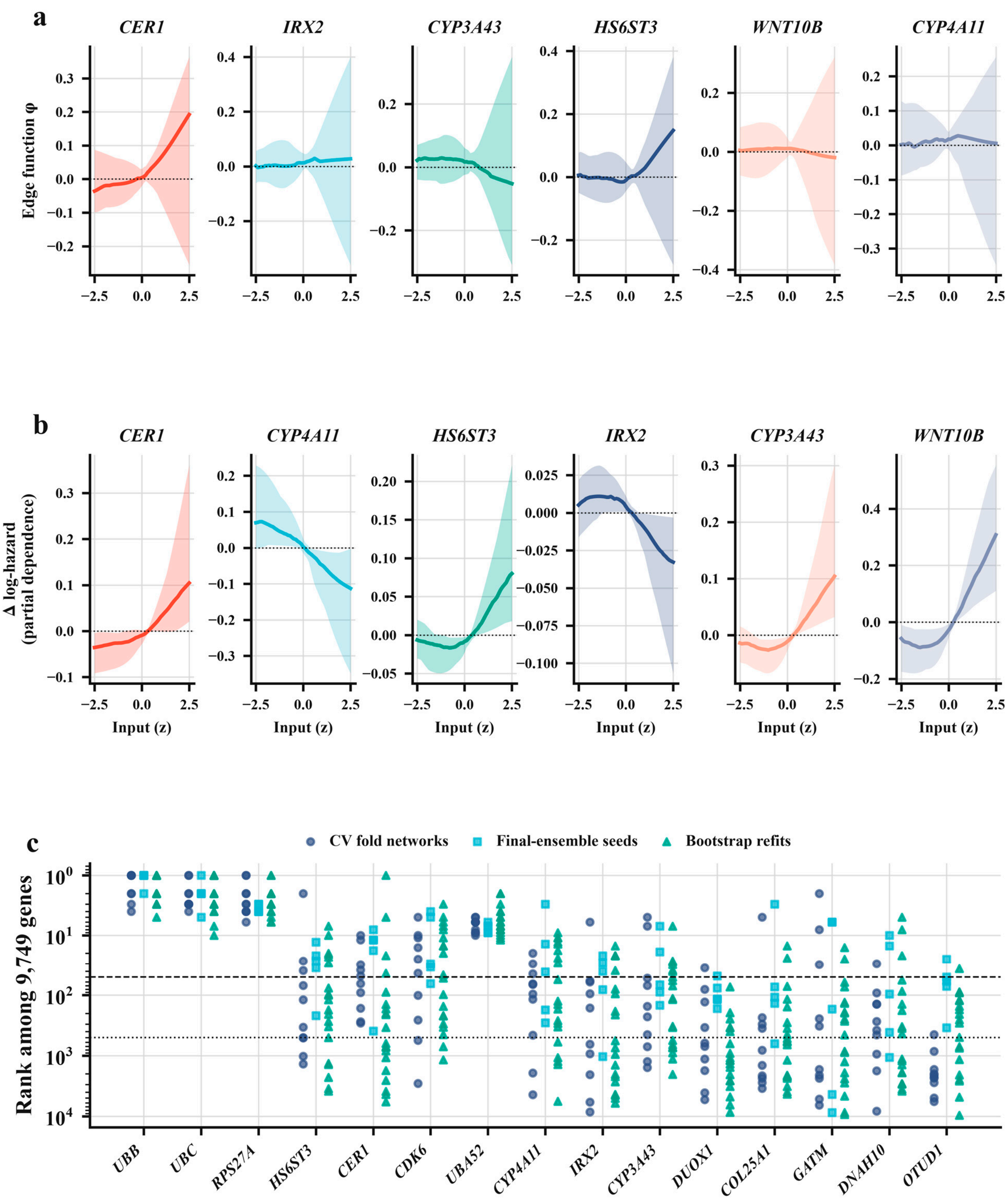

Figure S12. Stability of the interpretability readout across the ten cross-validation networks, the five final-ensemble seeds and 20 bootstrap refits. (a) The six edge functions of Figure 7a: median (line) and 2.5–97.5 percentile band across refits. (b) Gene-level partial dependence of the predicted log-hazard (mean change in model output as one gene's standardised input moves across its range, all other inputs at observed values). (c) Rank of the fifteen reported top genes among 9,749 genes in each refit; dashed and dotted lines mark ranks 50 and 500.

Table S1. Provenance of the four independent validation series, recorded at download.

GEO accession	Platform	Samples retrieved	Genes after collapse	Tumours analysed	Role	Endpoint(s) deposited
GSE39582	GPL570	585	21755	566	primary external	RFS + OS

					validation	
GSE17536	GPL570	177	21755	177	secondary external validation	OS + DFS
GSE33113	GPL570	96	21755	90	stage-II-specific validation	DFS
GSE87211	GPL13497	363	21754	203	rectal-specific validation	DFS + OS

Table S2. Full hyperparameter sweep: 24 configurations evaluated under inner three-fold cross-validation of the discovery cohort. This search was run once on the whole discovery cohort and selected the configuration of the final network; the nested selection used for all cross-validated estimates is given in Table S15.

Learning rate	L1 weight	Dropout	Epochs	Weight decay	C-index	SD	Runtime (s)
0.003	0.01	0.2	120	0.001	0.6084	0.0181	35.5
0.003	0.01	0.2	120	0.0001	0.6075	0.0152	37.1
0.003	0.0001	0.2	120	0.001	0.606	0.0215	35.4
0.003	0.001	0.2	120	0.001	0.6056	0.0247	35.3
0.003	0.0001	0.2	60	0.001	0.605	0.0114	17.9
0.003	0.01	0.4	120	0.001	0.6048	0.0233	35.4
0.001	0.0001	0.4	120	0.0001	0.6047	0.0348	35.6
0.003	0.0001	0.2	60	0.0001	0.6043	0.0117	18.0
0.001	0.001	0.4	60	0.0001	0.6028	0.0201	17.8
0.001	0.01	0.4	60	0.001	0.6027	0.0199	18.0
0.001	0.0001	0.4	60	0.0001	0.6026	0.0206	18.0
0.001	0.001	0.2	120	0.001	0.6021	0.0207	35.5
0.001	0.001	0.2	120	0.0001	0.6019	0.021	35.4
0.003	0.01	0.4	120	0.0001	0.6019	0.0232	35.5
0.001	0.0001	0.2	120	0.001	0.6019	0.0214	35.8
0.003	0.0001	0.4	120	0.001	0.601	0.0248	35.4
0.001	0.01	0.4	120	0.001	0.5993	0.0353	35.3
0.001	0.001	0.2	60	0.001	0.5975	0.0174	17.8
0.001	0.0001	0.2	60	0.0001	0.5973	0.0176	18.0
0.001	0.0001	0.2	60	0.001	0.5967	0.0181	17.7
0.001	0.01	0.2	60	0.001	0.5955	0.0193	18.0
0.003	0.001	0.4	60	0.001	0.5938	0.0235	17.8
0.003	0.0001	0.4	60	0.0001	0.5929	0.0219	17.9
0.003	0.001	0.4	60	0.0001	0.5916	0.0228	17.8

Table S3. Concordance of every model in each of the ten cross-validation folds, computed on identical splits. PathKAN values are from nested cross-validation; the clinical model uses stage completed from the TCGA Clinical Data Resource.

fold	clinical_only	coxnet_l1r0.5	coxnet_l1r0.9	pathkan	rsf
1	0.625	0.534	0.535	0.571	0.585
2	0.701	0.595	0.612	0.595	0.569
3	0.718	0.591	0.581	0.638	0.638
4	0.688	0.475	0.461	0.569	0.594
5	0.778	0.546	0.542	0.609	0.629
6	0.599	0.467	0.444	0.610	0.572
7	0.709	0.475	0.539	0.508	0.616
8	0.701	0.622	0.616	0.758	0.790
9	0.710	0.567	0.564	0.618	0.561
10	0.775	0.554	0.558	0.568	0.607

Table S4. Per-fold concordance for the clinical model, the molecular score alone, and the two combined. Out-of-fold PathKAN scores from nested cross-validation; stage completed from the TCGA Clinical Data Resource.

fold	clinical	clinical+pathkan	pathkan_only	delta
1	0.622	0.647	0.617	0.025
2	0.695	0.714	0.633	0.019
3	0.721	0.710	0.633	-0.011
4	0.689	0.703	0.579	0.013
5	0.774	0.792	0.608	0.018
6	0.597	0.610	0.614	0.013
7	0.709	0.709	0.540	0.001
8	0.701	0.746	0.771	0.045
9	0.709	0.720	0.635	0.010

10	0.777	0.758	0.570	-0.018

Table S5. Gene-level influence with connectivity adjustment, for the 1,000 highest-ranked genes.

Gene	Summed influence	Mean influence per edge	Max edge influence	Pathway edges	Degree bin	Influence z
UBB	28.0216	0.0865	0.1822	324	11	8.6896
UBC	27.3554	0.0877	0.201	312	11	8.453
RPS27A	23.5606	0.0725	0.1674	325	11	7.1052
HS6ST3	0.967	0.2418	0.3636	4	5	6.296
CER1	1.7468	0.2495	0.4321	7	8	6.2271
CDK6	3.3614	0.1867	0.3049	18	10	6.109
UBA52	20.0735	0.0623	0.1327	322	11	5.8666
CYP4A11	2.1798	0.1982	0.3382	11	9	5.8552
IRX2	0.5532	0.2766	0.4109	2	2	5.7926
CYP3A43	1.118	0.2236	0.3681	5	6	5.7271
DUOX1	0.5383	0.2692	0.2853	2	2	5.5541
COL25A1	1.0672	0.2134	0.2588	5	6	5.3098
GATM	0.2744	0.2744	0.2744	1	0	5.0914
DNAH10	0.6391	0.213	0.2989	3	3	5.0363
OTUD1	1.0233	0.2558	0.2944	4	6	4.949
IL36G	0.2693	0.2693	0.2693	1	0	4.9488
DAO	0.6311	0.2104	0.2846	3	3	4.9404
CHMP4A	2.9099	0.1617	0.2553	18	10	4.7998
LIPJ	0.4832	0.2416	0.2436	2	2	4.6703
GJB2	0.6439	0.2146	0.2775	3	4	4.6227
HRAS	16.5706	0.112	0.2255	148	11	4.6225
IRAK4	2.8435	0.1777	0.3011	16	10	4.6071
P4HA1	1.4662	0.1833	0.256	8	8	4.6061
ASPG	0.2565	0.2565	0.2565	1	0	4.5899
CYP1A1	2.8269	0.2175	0.3269	13	10	4.5591
CHN2	0.2547	0.2547	0.2547	1	0	4.5382
GRIK3	0.9668	0.1934	0.2437	5	6	4.4855
AKAP10	0.4037	0.2018	0.263	2	1	4.4038
CNPY3	0.4021	0.2011	0.2219	2	1	4.3797
EXT1	1.8447	0.1677	0.2425	11	9	4.3509
CTLA4	1.0998	0.1833	0.2175	6	7	4.3335
CD99	0.5804	0.1935	0.309	3	3	4.3288
DTNA	0.9465	0.1893	0.2714	5	6	4.3184
SYT1	2.7382	0.1825	0.2861	15	10	4.302
IL9R	0.6169	0.2056	0.2164	3	4	4.281
SLCO4C1	0.6165	0.2055	0.2293	3	4	4.2752
CHRNE	0.7627	0.1907	0.2175	4	5	4.2493
CYP17A1	1.0866	0.1811	0.3148	6	7	4.2365
LRP2	1.8156	0.1816	0.2404	10	9	4.2203
NCAPG2	0.6103	0.2034	0.2828	3	4	4.1971
GJA3	0.4536	0.2268	0.2512	2	2	4.1946
WNT10B	2.6988	0.2249	0.3384	12	10	4.1878
FOXH1	1.0776	0.1796	0.2587	6	7	4.1701
CAV3	0.754	0.1885	0.2825	4	5	4.1625
AQP10	0.3853	0.1926	0.2853	2	1	4.1103
DNAH9	0.5583	0.1861	0.2137	3	3	4.0629
RORB	0.599	0.1997	0.2476	3	4	4.0539
KCNJ3	1.0582	0.1764	0.2554	6	7	4.0272
CACNA2D4	0.5552	0.1851	0.1999	3	3	4.0246
MAPK1	14.8158	0.0909	0.1699	163	11	3.9992
CNOT10	1.0504	0.1751	0.2182	6	7	3.9697
MAT1A	1.3524	0.169	0.2523	8	8	3.9489
FAM169A	0.2326	0.2326	0.2326	1	0	3.9195
CSTA	0.3709	0.1855	0.1991	2	1	3.8812
MIP	0.4314	0.2157	0.271	2	2	3.8384
RBPJL	1.0297	0.2059	0.2423	5	7	3.8171
CCT6B	1.3273	0.1896	0.2428	7	8	3.8042
PTPRJ	1.7158	0.2145	0.3128	8	9	3.772
ANGPT1	1.3141	0.1643	0.3098	8	8	3.7279
HSPA6	0.8746	0.1749	0.2629	5	6	3.7274
BEGAIN	0.3608	0.1804	0.2275	2	1	3.7204
LPA	0.4237	0.2119	0.247	2	2	3.715
ANGPT2	1.0125	0.1687	0.2086	6	7	3.69
ALDH1L2	0.5264	0.1755	0.2075	3	3	3.6778
SLC6A20	1.0089	0.2018	0.3276	5	7	3.6639
MED28	1.0087	0.2017	0.3	5	7	3.662
AQP11	0.3568	0.1784	0.2527	2	1	3.6562
COL6A5	1.6855	0.1532	0.2389	11	9	3.6361
SRD5A2	0.7006	0.2335	0.2542	3	5	3.6272
GABRG2	0.6929	0.1732	0.2362	4	5	3.5507
GABRA5	0.5587	0.1862	0.2393	3	4	3.5434

DDX11	0.3488	0.1744	0.1862	2	1	3.5288
KLK8	0.8494	0.1699	0.19	5	6	3.5208
ALAS2	0.5119	0.1706	0.2115	3	3	3.5034
BBS5	0.6857	0.1714	0.2409	4	5	3.4783
NKX6-1	1.65	0.1833	0.2505	9	9	3.4766
CRYBA4	0.8439	0.1688	0.2485	5	6	3.4756
CLEC1B	0.5079	0.1693	0.2468	3	3	3.4541
EDA	0.4074	0.2037	0.2108	2	2	3.454
ADAMTS15	0.8411	0.1682	0.2141	5	6	3.452
MYLK3	0.2156	0.2156	0.2156	1	0	3.4404
ADAMTSL1	0.8393	0.1679	0.2139	5	6	3.4371
GCK	1.6406	0.1491	0.2232	11	9	3.4344
CHRD	0.2143	0.2143	0.2143	1	0	3.4062
CNGA4	0.9705	0.1618	0.2779	6	7	3.3809
CLEC10A	0.3394	0.1697	0.1933	2	1	3.3793
MAPK3	13.06	0.0816	0.154	160	11	3.3756
KCNQ2	0.83	0.166	0.2055	5	6	3.361
RBP1	1.2504	0.1786	0.3302	7	8	3.3598
KLC3	2.4086	0.172	0.2879	14	10	3.3462
DLK1	0.4988	0.1663	0.2133	3	3	3.3451
ELANE	1.6204	0.1473	0.2054	11	9	3.344
EXOC4	1.246	0.1557	0.2514	8	8	3.3341
KCTD15	0.2115	0.2115	0.2115	1	0	3.3279
ALOX15	0.9626	0.1604	0.2124	6	7	3.3225
GLP1R	0.9626	0.1604	0.2306	6	7	3.3225
PSMB2	12.894	0.1015	0.1679	127	11	3.3167
CALML5	0.6692	0.1673	0.2467	4	5	3.3135
DOCK11	0.4962	0.1654	0.1812	3	3	3.3133
KCNJ8	0.5403	0.1801	0.2444	3	4	3.31
PLEKHA4	0.5388	0.1796	0.2358	3	4	3.2914
FGF12	0.5378	0.1793	0.2378	3	4	3.2792
CABLES2	0.2095	0.2095	0.2095	1	0	3.2703
DPYS	0.4918	0.1639	0.205	3	3	3.261
DHRSX	0.4911	0.1637	0.1868	3	3	3.2516
PRKX	2.3728	0.1582	0.2857	15	10	3.2426
CYP26B1	1.5971	0.1597	0.2447	10	9	3.2394
SOS1	12.6706	0.0967	0.1655	131	11	3.2373
CLEC4M	0.3284	0.1642	0.2103	2	1	3.2035
HEYL	2.3593	0.1685	0.2484	14	10	3.2033
ADAMTS19	0.8094	0.1619	0.2252	5	6	3.1916
HTR7	0.8064	0.1613	0.1954	5	6	3.1669
FECH	0.6544	0.1636	0.2407	4	5	3.165
ATF5	0.6543	0.1636	0.2403	4	5	3.1642
EYA1	0.9383	0.1564	0.228	6	7	3.1428
TRDN	1.2102	0.1729	0.2229	7	8	3.1275
OCLN	1.2097	0.1512	0.1965	8	8	3.1246
GABRA4	0.5256	0.1752	0.1926	3	4	3.1243
TLE4	1.5698	0.157	0.2136	10	9	3.1168
MLLT3	0.3858	0.1929	0.213	2	2	3.1065
MTMR14	0.6465	0.1616	0.1986	4	5	3.0861
PSMD12	12.2288	0.0978	0.1864	125	11	3.0804
SLC4A8	0.5217	0.1739	0.2406	3	4	3.0745
GRB2	12.2052	0.0735	0.1408	166	11	3.072
IFI44L	0.7948	0.159	0.1995	5	6	3.0719
DAB1	0.2021	0.2021	0.2021	1	0	3.062
KCNK12	0.5206	0.1735	0.2238	3	4	3.0613
PXN	2.3068	0.1282	0.2025	18	10	3.0511
FLRT3	0.6428	0.1607	0.1748	4	5	3.0483
PSMA3	12.124	0.0947	0.1927	128	11	3.0432
CLVS1	0.4731	0.1577	0.223	3	3	3.0348
FTL	0.922	0.1537	0.1763	6	7	3.0233
PHKA1	0.5175	0.1725	0.2295	3	4	3.0219
ARSD	0.7879	0.1576	0.2569	5	6	3.015
NOD2	2.2909	0.1273	0.22	18	10	3.005
PRKACA	12.0042	0.1429	0.2414	84	11	3.0006
PELI1	1.5437	0.1544	0.2386	10	9	2.9997
MOBP	0.1992	0.1992	0.1992	1	0	2.9804
FOXM1	0.7817	0.1563	0.1879	5	6	2.9644
AOC2	0.3133	0.1566	0.1966	2	1	2.9623
KCNQ3	0.6338	0.1584	0.167	4	5	2.9583
PSMA8	1.5282	0.1698	0.2325	9	9	2.9299
CHRNA9	1.1751	0.1469	0.2202	8	8	2.9249
NEO1	0.3731	0.1865	0.2753	2	2	2.9025
KRAS	11.7177	0.0792	0.1625	148	11	2.8989
CYP4F12	0.7733	0.1547	0.2046	5	6	2.8951
HEY2	2.2518	0.1501	0.2415	15	10	2.8916
KRT38	0.3722	0.1861	0.1955	2	2	2.8891
PTPRZ1	0.372	0.186	0.26	2	2	2.8853
OPRM1	1.168	0.146	0.2166	8	8	2.8835

CAPN7	0.1955	0.1955	0.1955	1	0	2.8789
KCNH7	0.3714	0.1857	0.189	2	2	2.876
BNIP3	0.1953	0.1953	0.1953	1	0	2.872
CEBPE	0.1953	0.1953	0.1953	1	0	2.8719
PSMD6	11.6303	0.0938	0.1765	124	11	2.8678
MYRIP	0.9001	0.18	0.2331	5	7	2.8615
BBS4	0.624	0.156	0.197	4	5	2.8602
FBN2	0.6237	0.1559	0.2087	4	5	2.857
MCOLN3	0.5043	0.1681	0.1697	3	4	2.854
ATP6V1B1	1.511	0.1511	0.2103	10	9	2.8527
IL20RB	0.1946	0.1946	0.1946	1	0	2.8521
ALPP	0.3061	0.1531	0.1644	2	1	2.8482
ARPC5L	2.2363	0.1864	0.2836	12	10	2.8466
PSMC1	11.5624	0.0918	0.183	126	11	2.8437
BAG6	0.3056	0.1528	0.1576	2	1	2.8395
GRIA4	1.1599	0.145	0.1943	8	8	2.8369
GP9	1.5066	0.1507	0.2414	10	9	2.8331
PSMC3	11.5073	0.0921	0.1866	125	11	2.8242
OTUD7A	0.3681	0.1841	0.3028	2	2	2.8229
SIX2	0.4551	0.2276	0.2617	2	3	2.8182
HRG	0.7638	0.1528	0.2091	5	6	2.8168
CCNT1	2.2203	0.1234	0.188	18	10	2.8003
CRLF2	0.4535	0.1512	0.1908	3	3	2.7988
CAPNS1	1.1529	0.1441	0.2241	8	8	2.7966
STT3B	2.2156	0.1385	0.1923	16	10	2.7867
HACL1	0.8887	0.1481	0.2607	6	7	2.7771
PSMD1	11.3687	0.091	0.1814	125	11	2.7749
LPCAT4	1.1433	0.1633	0.2573	7	8	2.7412
KIF15	1.1418	0.1427	0.1793	8	8	2.7322
KIFAP3	1.4837	0.1349	0.1952	11	9	2.7301
IL19	0.3622	0.1811	0.1986	2	2	2.7284
PSMB3	11.2326	0.0891	0.168	126	11	2.7266
NRAS	11.2324	0.0791	0.1373	142	11	2.7265
SIX4	0.2983	0.2983	0.2983	1	1	2.7236
PSMA2	11.2192	0.0883	0.1513	127	11	2.7218
KIF7	0.8805	0.1468	0.1893	6	7	2.7171
IL17RB	0.6093	0.1523	0.2673	4	5	2.7135
EXOG	0.1892	0.1892	0.1892	1	0	2.7019
GNAL	1.4749	0.1341	0.2328	11	9	2.6906
MRPS21	0.4911	0.1637	0.2186	3	4	2.6869
PSMB1	11.109	0.0889	0.1676	125	11	2.6827
PLA1A	0.4905	0.1635	0.1829	3	4	2.679
DUSP14	0.1883	0.1883	0.1883	1	0	2.676
RPL27A	2.1764	0.1209	0.1882	18	10	2.673
PSMB4	11.0246	0.0882	0.1557	125	11	2.6527
GATA5	0.1874	0.1874	0.1874	1	0	2.6494
MSX2	0.3568	0.1784	0.1968	2	2	2.6423
SLC16A8	0.7422	0.1856	0.2012	4	6	2.6397
CNOT6L	0.8696	0.1449	0.2073	6	7	2.6368
IFNGR1	1.4626	0.133	0.2307	11	9	2.6354
ATG4A	0.4391	0.1464	0.1618	3	3	2.6257
NLGN1	0.3558	0.1779	0.2436	2	2	2.6253
SLC1A2	1.1221	0.187	0.2371	6	8	2.6187
FOXP2	0.8653	0.1442	0.1685	6	7	2.6047
PIK3CA	10.8892	0.0857	0.1601	127	11	2.6046
PSMA5	10.8858	0.0871	0.1682	125	11	2.6034
MUC1	2.1463	0.1192	0.1968	18	10	2.5858
CRTC3	0.8626	0.1438	0.183	6	7	2.5851
AKAP7	0.1849	0.1849	0.1849	1	0	2.58
LRRTM4	0.3529	0.1764	0.2211	2	2	2.5783
ONECUT1	1.1142	0.1393	0.2079	8	8	2.5729
COL9A2	1.4447	0.1313	0.1821	11	9	2.5549
CHCHD3	0.7314	0.1463	0.23	5	6	2.5508
LTBR	0.4797	0.1599	0.1853	3	4	2.5426
SLC13A1	0.4314	0.2157	0.269	2	3	2.5326
F3	1.4394	0.1439	0.2785	10	9	2.5313
USP18	2.1247	0.125	0.2009	17	10	2.5232
ALDH8A1	0.5903	0.1476	0.1975	4	5	2.5225
SRC	10.656	0.0839	0.1424	127	11	2.5218
HPN	0.4777	0.1592	0.1942	3	4	2.517
GGA2	0.5891	0.1473	0.1721	4	5	2.511
FGF11	0.4765	0.1588	0.197	3	4	2.5017
ATP13A5	0.2843	0.1421	0.1705	2	1	2.4996
HIF1AN	0.182	0.182	0.182	1	0	2.4994
PDE1C	0.8508	0.1418	0.1901	6	7	2.4984
PDS5B	1.43	0.1589	0.2681	9	9	2.4891
PSMD3	10.5315	0.0843	0.1583	125	11	2.4776
IFI44	0.5856	0.1464	0.1847	4	5	2.476
KCNK1	0.5855	0.1464	0.2325	4	5	2.4748

ILK	0.5852	0.1463	0.1953	4	5	2.4723
ABAT	0.7216	0.1443	0.1763	5	6	2.47
KIF6	0.7213	0.1443	0.1949	5	6	2.468
PSMD11	10.4905	0.0846	0.1783	124	11	2.463
CHST10	0.7203	0.144	0.2464	5	6	2.4593
COG3	1.0931	0.1562	0.295	7	8	2.4509
KRT14	1.4214	0.1421	0.1853	10	9	2.4503
THEM4	2.0977	0.1498	0.2123	14	10	2.4448
MMAA	1.0912	0.1364	0.2434	8	8	2.4401
MMP7	1.4189	0.1577	0.2396	9	9	2.4395
NEK11	0.4711	0.157	0.1835	3	4	2.4337
A4GNT	0.2801	0.1401	0.1406	2	1	2.4332
DIO3	0.3437	0.1718	0.2019	2	2	2.431
PSMB7	10.3891	0.0831	0.1636	125	11	2.427
ELOVL6	1.0879	0.136	0.2053	8	8	2.4208
GMPR	0.8396	0.1399	0.2079	6	7	2.4153
FBP2	0.5789	0.1447	0.1827	4	5	2.4087
PPP3CC	2.0841	0.1489	0.2143	14	10	2.4055
CPN2	0.1786	0.1786	0.1786	1	0	2.4035
BHLHE41	0.278	0.139	0.1401	2	1	2.3997
CCRL2	0.8373	0.1395	0.2686	6	7	2.3982
BDNF	2.0795	0.1223	0.1866	17	10	2.3919
CA6	0.2772	0.1386	0.1921	2	1	2.3869
LRP4	0.3407	0.1703	0.1923	2	2	2.3829
ACAN	1.4043	0.1404	0.2275	10	9	2.3739
CAMKK1	1.4029	0.1275	0.2326	11	9	2.3673
ESPNL	0.4657	0.1552	0.241	3	4	2.3655
ACR	0.2751	0.1375	0.1515	2	1	2.3522
GRIA1	2.0656	0.1475	0.2415	14	10	2.3516
GRM1	1.3991	0.1272	0.1701	11	9	2.3504
ESAM	0.1766	0.1766	0.1766	1	0	2.3459
POU3F3	0.3384	0.1692	0.2462	2	2	2.3456
KRT83	0.3381	0.169	0.1811	2	2	2.3414
HOXB1	0.3379	0.1689	0.2038	2	2	2.338
MAX	1.3962	0.1396	0.186	10	9	2.3375
GPR17	0.829	0.1382	0.1776	6	7	2.337
PSMD2	10.1345	0.0817	0.1835	124	11	2.3366
CD1A	0.176	0.176	0.176	1	0	2.3309
FZD10	0.828	0.138	0.176	6	7	2.3302
TERT	2.0578	0.1871	0.2818	11	10	2.3292
ACSS3	0.5703	0.1426	0.1861	4	5	2.3227
FOXG1	0.5699	0.1425	0.1708	4	5	2.319
ESRRB	0.1754	0.1754	0.1754	1	0	2.314
GC	0.4607	0.1536	0.2094	3	4	2.3023
CEP89	0.4112	0.1371	0.1857	3	3	2.289
TPH1	0.8224	0.1645	0.2172	5	7	2.2885
OAS2	1.0644	0.1521	0.2223	7	8	2.2852
DNAH3	0.4107	0.1369	0.177	3	3	2.2831
ALOX15B	0.5663	0.1416	0.164	4	5	2.2829
MCF2	2.0419	0.1571	0.267	13	10	2.2829
FOXD3	0.5656	0.1414	0.1946	4	5	2.2753
CPA3	0.2701	0.135	0.1611	2	1	2.2728
MECR	0.4577	0.1526	0.172	3	4	2.2636
COX6C	1.3789	0.1254	0.1627	11	9	2.2599
SLC6A15	0.4573	0.1524	0.2026	3	4	2.2586
PSMB6	9.9024	0.0792	0.138	125	11	2.2541
LGI3	0.1732	0.1732	0.1732	1	0	2.2516
PLEKHM1	0.3324	0.1662	0.2083	2	2	2.2507
JMJD6	0.6946	0.1389	0.1976	5	6	2.2481
CLEC4C	0.2684	0.1342	0.1517	2	1	2.2465
ALG11	0.6943	0.1389	0.2041	5	6	2.2462
BPIFB2	0.2684	0.1342	0.1415	2	1	2.2459
HPGD	1.057	0.151	0.237	7	8	2.2429
ANPEP	0.4072	0.1357	0.1814	3	3	2.2411
GPAM	2.0272	0.1267	0.1863	16	10	2.2404
PNPLA3	0.3318	0.1659	0.1753	2	2	2.24
MTMR6	0.455	0.1517	0.2304	3	4	2.2297
EPHA4	0.8142	0.1357	0.2194	6	7	2.2285
NKIRAS1	2.0224	0.1556	0.2489	13	10	2.2263
ELOVL7	0.4545	0.1515	0.1748	3	4	2.2232
MEP1B	0.172	0.172	0.172	1	0	2.2187
PSMB5	9.7936	0.0777	0.1831	126	11	2.2155
PSMC5	9.7888	0.0789	0.1508	124	11	2.2138
DNAH5	0.4049	0.135	0.1558	3	3	2.2132
OPCML	0.1717	0.1717	0.1717	1	0	2.2105
PSMC2	9.778	0.0782	0.1699	125	11	2.21
ADRM1	9.7741	0.077	0.1542	127	11	2.2086
ADH4	1.0509	0.1501	0.2045	7	8	2.2076
GNGT1	9.7689	0.1437	0.2393	68	11	2.2067

CALM1	9.7681	0.0864	0.1606	113	11	2.2064
FXYD1	1.0503	0.15	0.2397	7	8	2.2042
PCOLCE2	0.4529	0.151	0.2042	3	4	2.203
FFAR1	1.3657	0.1517	0.2205	9	9	2.2004
FXYD4	0.81	0.135	0.2022	6	7	2.1973
EFNB3	0.5576	0.1394	0.1793	4	5	2.195
PSMA1	9.7299	0.0772	0.1475	126	11	2.1929
OASL	0.8083	0.1347	0.2429	6	7	2.1844
CYP11A1	1.0463	0.1308	0.212	8	8	2.1806
GUCA1B	0.4511	0.1504	0.2079	3	4	2.18
CACNG8	1.0459	0.1307	0.2291	8	8	2.1787
KCNA10	0.3277	0.1639	0.167	2	2	2.1754
PIK3CB	9.6747	0.118	0.2149	82	11	2.1733
BST2	1.0448	0.1306	0.1852	8	8	2.1722
KIF2A	2.0034	0.1178	0.1753	17	10	2.1713
DSCAM	0.1703	0.1703	0.1703	1	0	2.1705
PPIL3	0.4503	0.1501	0.201	3	4	2.1703
WNT4	2.0006	0.1177	0.1862	17	10	2.1632
DEPTOR	0.6837	0.1367	0.193	5	6	2.1591
KRT31	0.4492	0.1497	0.2366	3	4	2.1567
BSN	0.2628	0.1314	0.1555	2	1	2.1566
ATRIP	1.998	0.1249	0.1725	16	10	2.1559
CCT5	1.3556	0.1232	0.1782	11	9	2.1551
A1BG	0.2625	0.1313	0.1687	2	1	2.1526
SLC30A8	0.8032	0.1606	0.2084	5	7	2.1471
CDK5R2	0.3994	0.1331	0.1599	3	3	2.146
CNP	0.2621	0.131	0.1484	2	1	2.1452
ACAT1	1.3533	0.123	0.1488	11	9	2.145
PSMA7	9.5934	0.0761	0.1433	126	11	2.1444
EFNA2	0.8028	0.1338	0.2256	6	7	2.1439
ASH2L	1.9934	0.1107	0.1649	18	10	2.1424
BDH2	0.2617	0.1308	0.183	2	1	2.1385
GPX7	0.5519	0.138	0.2318	4	5	2.138
DNAI1	0.3986	0.1329	0.1665	3	3	2.1364
PSMD8	9.57	0.0766	0.1314	125	11	2.1361
ICAM5	0.4475	0.1492	0.2443	3	4	2.1348
BAAT	1.0381	0.1483	0.2137	7	8	2.1335
PSMD13	9.5622	0.0765	0.1632	125	11	2.1333
DNAH1	0.3983	0.1328	0.1729	3	3	2.1328
NOSTRIN	0.1689	0.1689	0.1689	1	0	2.1301
NEU1	1.3498	0.135	0.1865	10	9	2.1292
CRP	0.5509	0.1377	0.2	4	5	2.1287
OSGEPL1	0.1686	0.1686	0.1686	1	0	2.1238
ACOT13	0.2606	0.1303	0.1307	2	1	2.1215
INHBC	0.1685	0.1685	0.1685	1	0	2.1203
GOLGA3	0.1684	0.1684	0.1684	1	0	2.1179
MADD	0.6784	0.1357	0.1594	5	6	2.115
MOGS	1.981	0.1165	0.1788	17	10	2.1063
DPPA4	0.3959	0.132	0.1681	3	3	2.1047
BAG3	0.3957	0.1319	0.1465	3	3	2.1019
BEST2	0.2593	0.1297	0.153	2	1	2.1014
GAS2	0.6767	0.1353	0.23	5	6	2.1012
TRIM5	0.5474	0.1825	0.2061	3	5	2.0928
ATP6V0A1	1.3409	0.1341	0.2091	10	9	2.0891
SMARCD2	1.974	0.1234	0.1694	16	10	2.0861
CPNE3	0.2584	0.1292	0.1375	2	1	2.0858
ATP11B	0.2583	0.1291	0.1383	2	1	2.0843
EDA2R	0.322	0.161	0.1653	2	2	2.0826
DCLRE1B	0.3941	0.1314	0.1632	3	3	2.0825
PSMA4	9.4111	0.0741	0.1476	127	11	2.0796
IFT74	0.4431	0.1477	0.204	3	4	2.079
CLEC2B	0.167	0.167	0.167	1	0	2.0788
BTBD6	0.2578	0.1289	0.1295	2	1	2.0777
PRMT7	0.3216	0.1608	0.229	2	2	2.0768
COX6B1	1.3377	0.1216	0.2055	11	9	2.0749
PCGF1	0.1668	0.1668	0.1668	1	0	2.0729
ATP1B2	1.0272	0.1284	0.189	8	8	2.0706
SENP8	0.4422	0.1474	0.1659	3	4	2.0678
BMP2K	0.2571	0.1285	0.1496	2	1	2.0658
IL1A	1.9657	0.1156	0.1794	17	10	2.062
LY6K	0.1664	0.1664	0.1664	1	0	2.0604
MORC2	0.5441	0.136	0.1653	4	5	2.0599
RAD51C	1.9649	0.1228	0.174	16	10	2.0599
CD9	1.334	0.1213	0.1457	11	9	2.0583
HAS2	0.6712	0.1342	0.1664	5	6	2.0566
KCNE1	0.4412	0.1471	0.1933	3	4	2.0556
NFIB	0.4412	0.1471	0.1979	3	4	2.0551
CRTAP	0.2564	0.1282	0.1492	2	1	2.0549
CHAT	0.7894	0.1316	0.201	6	7	2.0454

KDM5D	0.3193	0.1597	0.1598	2	2	2.0401
CYP4F3	1.3298	0.1478	0.2273	9	9	2.0394
PIK3R1	9.2929	0.0801	0.1552	116	11	2.0377
GBF1	1.9557	0.1304	0.2108	15	10	2.033
GATA3	1.954	0.1149	0.1728	17	10	2.0281
BCDIN3D	0.2547	0.1274	0.1304	2	1	2.0277
TBXAS1	1.3263	0.1474	0.2308	9	9	2.0238
G6PC2	0.5403	0.1351	0.1642	4	5	2.0226
USP7	1.9515	0.1301	0.2316	15	10	2.0209
PRDM4	0.4385	0.1462	0.1568	3	4	2.0206
MTMR4	1.0158	0.127	0.2007	8	8	2.0046
ADAMTS17	0.6649	0.133	0.2167	5	6	2.0046
DDHD2	0.3876	0.1292	0.1359	3	3	2.0045
ADAM20	0.253	0.1265	0.1394	2	1	2.0008
KRT37	0.3168	0.1584	0.1659	2	2	1.999
DNAH11	0.387	0.129	0.1555	3	3	1.9972
MDK	0.7827	0.1304	0.1764	6	7	1.9957
MAPT	1.9416	0.1079	0.2236	18	10	1.9922
WDR48	1.0134	0.1448	0.1988	7	8	1.9905
ATP6V1G2	1.0126	0.1266	0.1686	8	8	1.986
NEURL1B	1.317	0.1463	0.2051	9	9	1.9818
DNAJC24	0.1635	0.1635	0.1635	1	0	1.9794
DGKK	0.3855	0.1285	0.1553	3	3	1.9783
OR2C3	0.3149	0.1575	0.1898	2	2	1.97
ATG13	0.251	0.1255	0.1393	2	1	1.9686
ANGPT4	0.6605	0.1321	0.1807	5	6	1.968
GTF3A	0.3146	0.1573	0.1985	2	2	1.9647
CHSY3	0.5344	0.1336	0.1773	4	5	1.9632
PTH1R	0.6594	0.1648	0.2428	4	6	1.9592
RPL22L1	1.9298	0.1287	0.2004	15	10	1.9581
PSMC4	9.0536	0.0713	0.1445	127	11	1.9527
GCGR	0.7761	0.1293	0.2306	6	7	1.947
TRRAP	1.0053	0.1436	0.2314	7	8	1.9437
SIK1	0.4324	0.1441	0.2591	3	4	1.9431
ARSJ	0.6573	0.1315	0.1691	5	6	1.9417
MED7	1.0049	0.1256	0.173	8	8	1.9416
ATP1A1	1.0047	0.1256	0.1681	8	8	1.9404
GPR150	0.162	0.162	0.162	1	0	1.9375
HAVCR1	0.5318	0.1329	0.1925	4	5	1.9367
CUBN	1.3067	0.1307	0.2266	10	9	1.9354
CLDN19	0.6565	0.1313	0.2144	5	6	1.9353
AK3	0.3818	0.1273	0.1391	3	3	1.9349
IL20RA	0.3126	0.1563	0.1784	2	2	1.9328
CNGB1	1.3053	0.145	0.1961	9	9	1.9295
AQP2	0.3813	0.1271	0.1669	3	3	1.9286
EPHB4	0.6557	0.1311	0.1992	5	6	1.9286
TUBA8	8.9799	0.1247	0.2012	72	11	1.9265
ACOT11	0.2483	0.1241	0.1684	2	1	1.9254
GLIPR1	0.3121	0.1561	0.1652	2	2	1.9251
AKT1	8.9742	0.0816	0.1521	110	11	1.9244
GRM5	1.0015	0.1252	0.1996	8	8	1.9222
DERA	0.3118	0.1559	0.1749	2	2	1.9188
ALDH1A2	0.5298	0.1325	0.2213	4	5	1.9173
ENTPD3	0.3115	0.1558	0.1597	2	2	1.9147
BAIAP2L2	0.1611	0.1611	0.1611	1	0	1.9112
GGT1	1.9134	0.1367	0.2316	14	10	1.9104
GXYLT2	0.4297	0.1432	0.1725	3	4	1.9095
MED9	0.7709	0.1542	0.239	5	7	1.9089
GPR143	0.7709	0.1285	0.2139	6	7	1.9086
RPL32	1.912	0.1195	0.1771	16	10	1.9064
EOMES	0.9983	0.1248	0.1847	8	8	1.9038
RDH16	0.9983	0.1426	0.1654	7	8	1.9034
CDK8	1.9106	0.1124	0.1543	17	10	1.9025
SLC4A1	0.998	0.1426	0.2138	7	8	1.9019
CERS3	0.2464	0.1232	0.136	2	1	1.8953
GATA1	0.769	0.1282	0.1785	6	7	1.8951
CNTN5	0.1605	0.1605	0.1605	1	0	1.8946
EP300	8.886	0.0863	0.1645	103	11	1.8931
RBL1	1.9062	0.1466	0.2507	13	10	1.8895
BECN1	1.9061	0.1059	0.1514	18	10	1.8893
RORC	0.9955	0.1422	0.2453	7	8	1.8871
APOH	0.3778	0.1259	0.1346	3	3	1.8859
PTPRK	0.3096	0.1548	0.2342	2	2	1.8847
ABHD12	0.3776	0.1259	0.1897	3	3	1.8833
HS3ST2	0.5261	0.1315	0.1731	4	5	1.8802
CERS1	0.2453	0.1226	0.1488	2	1	1.8774
EXO1	1.9002	0.1056	0.1753	18	10	1.8723
ART3	0.2447	0.1224	0.1828	2	1	1.8685
EPHB1	0.5249	0.1312	0.1743	4	5	1.8677

ATP10B	0.2446	0.1223	0.1265	2	1	1.8672
GRK1	0.4263	0.1421	0.1674	3	4	1.8667
PYGL	0.6481	0.162	0.2805	4	6	1.8663
ELMOD3	0.1594	0.1594	0.1594	1	0	1.8644
THEM5	0.376	0.188	0.1958	2	3	1.8642
MKNK2	0.3083	0.1542	0.1675	2	2	1.8638
FBXO7	0.4257	0.1419	0.1745	3	4	1.8593
AGPAT2	0.764	0.1273	0.1924	6	7	1.8579
PIGL	0.4252	0.1417	0.1796	3	4	1.853
DPY30	1.8926	0.1051	0.1986	18	10	1.8502
RNASE1	0.7629	0.1271	0.1816	6	7	1.8499
NFKB1	8.7638	0.1109	0.187	79	11	1.8497
NAMPT	1.2867	0.117	0.1965	11	9	1.8459
C9	0.3744	0.1248	0.1504	3	3	1.8452
GJD4	0.3071	0.1536	0.1754	2	2	1.8444
PPARG	1.8895	0.105	0.1643	18	10	1.8411
TUBB2B	8.735	0.1149	0.1961	76	11	1.8395
ANKRD28	0.987	0.1234	0.1815	8	8	1.8385
DCP1B	0.2428	0.1214	0.1327	2	1	1.8378
IGFBP1	1.2848	0.1428	0.2023	9	9	1.8374
BRD9	0.3737	0.1246	0.1487	3	3	1.8367
KRT27	0.3065	0.1533	0.1956	2	2	1.8351
TAP1	1.2841	0.1284	0.2664	10	9	1.8342
POLR1D	1.8866	0.1258	0.2148	15	10	1.8329
GJC1	0.3063	0.1532	0.1763	2	2	1.8319
ARFRP1	0.2424	0.1212	0.1354	2	1	1.8315
PIGO	0.4231	0.141	0.1983	3	4	1.8261
CHAC1	0.7596	0.1266	0.1542	6	7	1.8254
STAR	1.2818	0.1602	0.2397	8	9	1.8236
ALPK1	0.9843	0.123	0.1968	8	8	1.8229
YBX1	1.8821	0.1176	0.1735	16	10	1.8198
GSTP1	1.2806	0.1281	0.1819	10	9	1.8186
ING2	0.5198	0.13	0.1531	4	5	1.8171
LPO	0.1577	0.1577	0.1577	1	0	1.8162
CTDP1	1.2801	0.1422	0.1869	9	9	1.816
B3GAT2	0.372	0.124	0.1474	3	3	1.8155
MYB	1.2799	0.1164	0.1687	11	9	1.8153
ATP13A2	0.2413	0.1207	0.1276	2	1	1.8141
ITGA3	1.8789	0.1342	0.1776	14	10	1.8104
POLR3GL	0.9822	0.1403	0.2254	7	8	1.8104
KRT75	0.3049	0.1524	0.2014	2	2	1.8081
PSMD7	8.6441	0.0692	0.1235	125	11	1.8072
MCM9	0.1573	0.1573	0.1573	1	0	1.807
CYP4B1	1.2781	0.1278	0.1906	10	9	1.807
S100A10	0.4215	0.1405	0.1775	3	4	1.8055
ATN1	0.3711	0.1237	0.1495	3	3	1.8054
IFT88	1.2772	0.1161	0.213	11	9	1.803
PKN3	0.7562	0.1512	0.2005	5	7	1.8006
IGLL1	0.1571	0.1571	0.1571	1	0	1.799
FOXP3	0.157	0.157	0.157	1	0	1.7968
KAT8	0.6395	0.1279	0.172	5	6	1.7956
ATP6V0A4	0.9792	0.1399	0.2095	7	8	1.793
PITPNM3	0.3039	0.152	0.155	2	2	1.7929
BORA	0.3701	0.1234	0.1389	3	3	1.7926
HOXC4	0.1567	0.1567	0.1567	1	0	1.7891
DIO1	0.5168	0.1292	0.1541	4	5	1.7868
NCAPD3	0.4197	0.1399	0.2129	3	4	1.7834
PRKACG	8.5619	0.1381	0.2223	62	11	1.778
FBXW12	0.3029	0.1514	0.1644	2	2	1.7765
ASS1	0.3685	0.1228	0.1345	3	3	1.7743
GALNT8	0.3027	0.1514	0.1765	2	2	1.7739
CLEC4E	0.2388	0.1194	0.1218	2	1	1.7735
DNALI1	0.3683	0.1228	0.1703	3	3	1.7717
NPY	0.7522	0.1504	0.3072	5	7	1.7712
CAND1	0.2386	0.1193	0.1401	2	1	1.7706
LIPC	0.5151	0.1288	0.1683	4	5	1.7701
MLKL	1.2693	0.141	0.1812	9	9	1.7677
MYL6B	0.4184	0.1395	0.1773	3	4	1.7661
CLCN4	0.2382	0.1191	0.1318	2	1	1.765
HTR2B	0.5144	0.1286	0.1865	4	5	1.7632
LIMK2	0.9737	0.1391	0.2016	7	8	1.7613
METTL22	0.1557	0.1557	0.1557	1	0	1.7607
DNAH12	0.3671	0.1224	0.142	3	3	1.7576
AFMID	0.2378	0.1189	0.1271	2	1	1.7575
EPM2A	0.7501	0.125	0.1812	6	7	1.7553
OTUD7B	0.75	0.125	0.2071	6	7	1.7551
CWF19L2	0.2375	0.1188	0.1355	2	1	1.754
GNA14	1.8593	0.1033	0.1728	18	10	1.7535
PARN	0.9722	0.1215	0.1982	8	8	1.7528

CD63	0.3667	0.1222	0.132	3	3	1.7521
GPD1L	0.513	0.1283	0.1514	4	5	1.7488
LEFTY1	0.1553	0.1553	0.1553	1	0	1.7486
KREMEN1	0.5128	0.1282	0.1571	4	5	1.7464
EGFR	8.4694	0.1046	0.2099	81	11	1.7452
BMPR1B	0.7485	0.1247	0.2569	6	7	1.7435
MGAM	0.3007	0.1503	0.1619	2	2	1.741
DYNC2H1	0.9698	0.1385	0.1996	7	8	1.7387
MOGAT1	0.3004	0.1502	0.1562	2	2	1.7367
WNT2B	1.2623	0.1262	0.1721	10	9	1.7364
CDS1	0.2364	0.1182	0.1419	2	1	1.7352
AOX1	0.3653	0.1218	0.1584	3	3	1.7352
IL21	0.4155	0.1385	0.1474	3	4	1.7297
F8	1.8498	0.1233	0.1824	15	10	1.7262
BCL2L13	0.1544	0.1544	0.1544	1	0	1.7242
ARPP21	0.1541	0.1541	0.1541	1	0	1.7168
EXOC8	0.9658	0.138	0.1589	7	8	1.716
CHGA	0.2352	0.1176	0.1426	2	1	1.7159
PITPNC1	0.2349	0.2349	0.2349	1	1	1.7124
HMG20B	0.5093	0.1273	0.1732	4	5	1.7119
CP	0.9651	0.1206	0.1626	8	8	1.7118
ATP7B	0.2349	0.1174	0.1249	2	1	1.7116
ATP8A2	0.2348	0.1174	0.1375	2	1	1.7105
MAP2K1	8.3666	0.1073	0.2545	78	11	1.7087
RAB39B	0.4138	0.1379	0.1906	3	4	1.7081
PSMD14	8.359	0.0658	0.114	127	11	1.706
CXCL16	0.6283	0.1257	0.1393	5	6	1.7039
P4HA2	0.9626	0.1375	0.1991	7	8	1.6975
GART	0.4128	0.1376	0.198	3	4	1.696
DISP2	0.2978	0.1489	0.1999	2	2	1.6951
PTPN21	0.2976	0.1488	0.1522	2	2	1.6918
ASNS	1.2521	0.1138	0.2038	11	9	1.6905
FBXW7	1.2518	0.1138	0.1633	11	9	1.6891
TP53	8.311	0.1026	0.1834	81	11	1.6889
THBD	0.741	0.1482	0.193	5	7	1.6882
GSTM3	0.961	0.1201	0.1895	8	8	1.6881
PTGER2	0.7409	0.1482	0.1913	5	7	1.688
SLC30A3	0.7407	0.1481	0.2232	5	7	1.6865
PTPN7	0.7406	0.1234	0.2139	6	7	1.6857
HIPK2	1.8358	0.1311	0.1835	14	10	1.6853
ADORA1	0.361	0.1203	0.1359	3	3	1.6839
MPO	0.2968	0.1484	0.1825	2	2	1.6796
SULT4A1	0.506	0.1687	0.2272	3	5	1.679
CLDN12	0.3606	0.1202	0.1546	3	3	1.6788
PAN2	0.4114	0.1371	0.1681	3	4	1.6776
ACTN3	0.7394	0.1232	0.1946	6	7	1.6769
SYCE3	0.5057	0.1686	0.1747	3	5	1.6755
ANO5	0.6247	0.1249	0.2159	5	6	1.6743
IFNGR2	1.247	0.1134	0.2025	11	9	1.6676
CAPN3	0.232	0.116	0.1311	2	1	1.6656
DNAH17	0.3594	0.1198	0.1564	3	3	1.6642
B3GNT4	0.7376	0.1229	0.1595	6	7	1.6634
AGTPBP1	0.1521	0.1521	0.1521	1	0	1.6607
CBS	0.3591	0.1197	0.1693	3	3	1.6602
PDE6B	0.9559	0.1195	0.191	8	8	1.6584
ANLN	0.5038	0.126	0.1807	4	5	1.657
LHX2	0.6225	0.1245	0.1628	5	6	1.656
DSCC1	0.7365	0.1228	0.18	6	7	1.6555
TNFRSF1B	1.2441	0.1555	0.226	8	9	1.6547
HSPA1B	1.244	0.1131	0.144	11	9	1.6543
FOLH1	0.1518	0.1518	0.1518	1	0	1.6525
CEP41	1.243	0.1381	0.2481	9	9	1.6495
PI4KA	0.4091	0.1364	0.175	3	4	1.6491
GRB14	0.2947	0.1473	0.176	2	2	1.6451
PDCD1LG2	0.735	0.1225	0.1608	6	7	1.6445
GRIK1	0.5025	0.1256	0.1852	4	5	1.6439
MYBL1	0.4085	0.1362	0.1722	3	4	1.6407
ANK3	1.2407	0.1128	0.142	11	9	1.6395
ATP4B	0.2304	0.1152	0.1295	2	1	1.6394
EPHA8	0.4082	0.1361	0.2154	3	4	1.6378
RPL15	1.8194	0.1137	0.1616	16	10	1.6378
SLC16A7	0.4082	0.1361	0.1661	3	4	1.6372
HSD17B12	0.62	0.124	0.1577	5	6	1.6351
ACTB	8.1547	0.1032	0.2129	79	11	1.6334
ARSG	0.6193	0.1239	0.1971	5	6	1.63
HAMP	0.1509	0.1509	0.1509	1	0	1.6263
KRT40	0.2935	0.1467	0.1857	2	2	1.6257
DCT	0.5006	0.1252	0.1432	4	5	1.6248
AZI2	0.2293	0.1146	0.1311	2	1	1.6224

NAGLU	0.9494	0.1356	0.2008	7	8	1.621
DNAH7	0.3558	0.1186	0.1409	3	3	1.621
LY6H	0.1507	0.1507	0.1507	1	0	1.6202
LAMA2	1.8129	0.1133	0.1767	16	10	1.6189
BCL2L14	0.2291	0.1145	0.1176	2	1	1.6185
TUBB1	8.1121	0.1082	0.1889	75	11	1.6183
CYTH1	0.6178	0.1236	0.1602	5	6	1.6173
TUBA1C	8.1086	0.1081	0.1832	75	11	1.617
CLINT1	0.2289	0.1145	0.1282	2	1	1.6161
GAST	0.2927	0.1464	0.1628	2	2	1.6137
AARS2	0.3552	0.1184	0.1511	3	3	1.613
MAPK8IP2	0.1504	0.1504	0.1504	1	0	1.612
PLEKHG4	0.4062	0.1354	0.1472	3	4	1.6119
CPB1	0.7305	0.1218	0.1768	6	7	1.6114
EVI5	0.1502	0.1502	0.1502	1	0	1.6077
ID4	0.6164	0.1233	0.1669	5	6	1.606
HELQ	0.1501	0.1501	0.1501	1	0	1.6042
PSMC6	8.0591	0.0625	0.1125	129	11	1.5995
ART1	0.2278	0.1139	0.1144	2	1	1.5983
ERCC4	1.8056	0.129	0.2411	14	10	1.5978
PLCD1	0.2917	0.1458	0.1499	2	2	1.5969
KRT12	0.2915	0.1458	0.2048	2	2	1.5942
EPHX3	0.1497	0.1497	0.1497	1	0	1.5934
HLCS	0.6147	0.1229	0.1733	5	6	1.5918
ALS2CL	0.2273	0.1137	0.1315	2	1	1.5912
IKZF4	0.1496	0.1496	0.1496	1	0	1.5899
GABRB2	0.4971	0.1243	0.1585	4	5	1.5892
ATG5	0.9436	0.118	0.1716	8	8	1.5877
PSME3	0.2911	0.1455	0.1829	2	2	1.5871
AK8	0.227	0.1135	0.1376	2	1	1.5864
IL17F	0.6138	0.1228	0.1612	5	6	1.5845
NDE1	1.8006	0.1059	0.1426	17	10	1.5835
TIAM2	0.9429	0.1347	0.1824	7	8	1.5835
CATSPERG	0.2268	0.1134	0.1158	2	1	1.5831
CNTF	0.3523	0.1174	0.1499	3	3	1.5785
ALDH4A1	0.1492	0.1492	0.1492	1	0	1.5785
ACP6	0.3522	0.1174	0.1339	3	3	1.5779
FUT1	0.613	0.1226	0.1618	5	6	1.5779
ARID3A	0.2263	0.1132	0.1223	2	1	1.5746
DMKN	0.352	0.1173	0.1614	3	3	1.5744
CENPW	0.4956	0.1239	0.1586	4	5	1.574
ADAT1	0.2262	0.1131	0.1283	2	1	1.5723
KCNAB1	0.2901	0.145	0.1491	2	2	1.5707
CACNA1S	0.7249	0.1208	0.2523	6	7	1.5699
SULT2A1	0.9402	0.1567	0.2222	6	8	1.5678
MAPKAP1	1.7951	0.1197	0.1584	15	10	1.5675
EEPD1	0.4025	0.1342	0.1991	3	4	1.5655
EGR4	0.4025	0.1342	0.162	3	4	1.5655
BBS10	0.4945	0.1236	0.1584	4	5	1.5637
CACNA1I	0.724	0.1207	0.1706	6	7	1.5632
SH3GL2	1.7936	0.0996	0.1646	18	10	1.5631
MKKS	0.4942	0.1236	0.1716	4	5	1.5607
HES5	1.2228	0.1112	0.1656	11	9	1.5587
SHH	1.7915	0.1378	0.1911	13	10	1.5571
NECAP1	0.4938	0.1235	0.1347	4	5	1.5565
KRT35	0.2891	0.1446	0.1722	2	2	1.5554
GLB1L2	1.2219	0.1358	0.2102	9	9	1.555
ARHGEF33	0.6099	0.122	0.1848	5	6	1.5529
KIF18B	0.9364	0.1338	0.2138	7	8	1.546
CYP4F22	1.2197	0.1109	0.2169	11	9	1.5452
IL22RA2	0.148	0.148	0.148	1	0	1.5452
FOXA1	0.6087	0.1217	0.155	5	6	1.543
DGKH	0.4924	0.1231	0.14	4	5	1.5422
DGKD	0.3492	0.1164	0.1175	3	3	1.5414
CD200	0.1479	0.1479	0.1479	1	0	1.5409
IFT140	0.6084	0.1217	0.1838	5	6	1.5401
EDAR	0.4005	0.1335	0.2105	3	4	1.5396
ARL4A	0.4921	0.123	0.2251	4	5	1.5392
FABP7	0.6078	0.1216	0.1426	5	6	1.5353
OXSM	0.1476	0.1476	0.1476	1	0	1.5344
CAPN13	0.1476	0.1476	0.1476	1	0	1.5343
RNASEL	0.6076	0.1519	0.2201	4	6	1.5334
ACAA1	1.2156	0.1105	0.1706	11	9	1.5264
ACAD9	0.3479	0.116	0.1818	3	3	1.5258
GABRQ	0.4905	0.1226	0.1365	4	5	1.5237
BBS2	0.4903	0.1226	0.1493	4	5	1.5216
CYB5R4	0.1472	0.1472	0.1472	1	0	1.5216
KAZN	0.399	0.133	0.1628	3	4	1.5204
MFNG	0.4901	0.1225	0.1936	4	5	1.5194

ABHD6	0.49	0.1225	0.1387	4	5	1.5187
KLHL3	0.2868	0.1434	0.1496	2	2	1.5181
CCNB3	0.2227	0.1114	0.1171	2	1	1.5177
FLT1	0.7178	0.1196	0.1565	6	7	1.5177
MAG	0.6056	0.1211	0.1568	5	6	1.5171
CYP2U1	0.9313	0.1164	0.2007	8	8	1.5163
DLX6	0.2864	0.1432	0.1915	2	2	1.5115
WASF2	1.7755	0.1184	0.1675	15	10	1.5105
BDH1	0.2222	0.1111	0.1291	2	1	1.5093
AKAP8L	0.3463	0.1154	0.1324	3	3	1.5057
ABHD14B	0.346	0.1153	0.1323	3	3	1.5031
KCNB2	0.2858	0.1429	0.145	2	2	1.5016
ADAM23	0.2217	0.1108	0.1151	2	1	1.5008
CEACAM5	0.2217	0.1108	0.1454	2	1	1.5005
GEMIN8	0.4882	0.122	0.1898	4	5	1.5001
GNB1	7.7737	0.1065	0.1977	73	11	1.4981
DZIP3	0.1463	0.1463	0.1463	1	0	1.4977
COQ10A	0.3456	0.1152	0.1354	3	3	1.4974
DYNLT3	0.4879	0.122	0.1526	4	5	1.4971
RPE	0.3969	0.1323	0.1584	3	4	1.4944
ANO4	0.6028	0.1206	0.1444	5	6	1.4939
CUL9	0.1461	0.1461	0.1461	1	0	1.4908
IFT57	0.6021	0.1204	0.1595	5	6	1.4886
AKR1B1	0.3448	0.1149	0.1473	3	3	1.4878
ALCAM	0.3448	0.1149	0.1498	3	3	1.4876
RAB32	0.3963	0.1321	0.1794	3	4	1.4871
GRM6	0.2848	0.1424	0.1661	2	2	1.4862
CSF2	1.2065	0.1097	0.1695	11	9	1.4858
CDH6	0.6016	0.1203	0.2211	5	6	1.4841
MAD1L1	1.2061	0.134	0.2468	9	9	1.4841
MFAP2	0.2846	0.1423	0.1941	2	2	1.483
ULBP2	0.2205	0.2205	0.2205	1	1	1.4813
SLC47A2	0.3442	0.1721	0.2113	2	3	1.4808
DLL3	0.6012	0.1202	0.1885	5	6	1.4807
HSF1	1.2053	0.1339	0.1823	9	9	1.4804
MRPS6	0.3958	0.1319	0.1902	3	4	1.4798
GRP	0.7127	0.1188	0.1382	6	7	1.4796
MAPK7	1.7641	0.1176	0.1494	15	10	1.4776
JAGN1	0.1456	0.1456	0.1456	1	0	1.476
CBY1	0.3438	0.1146	0.1333	3	3	1.476
CLCA1	0.22	0.11	0.1614	2	1	1.4742
ORM2	0.284	0.142	0.1801	2	2	1.4735
AHSP	0.1455	0.1455	0.1455	1	0	1.4734
STX16	0.6002	0.15	0.1934	4	6	1.4726
MYO9A	0.395	0.1317	0.138	3	4	1.47
PPBP	0.9232	0.1319	0.1841	7	8	1.4695
EFHD2	0.1452	0.1452	0.1452	1	0	1.4672
DNM3	0.9226	0.1318	0.1794	7	8	1.4664
CEACAM3	0.1452	0.1452	0.1452	1	0	1.4658
DCAF10	0.2194	0.1097	0.1313	2	1	1.4651
SLC9A2	0.3428	0.1714	0.1844	2	3	1.4644
PDCD6IP	1.2016	0.1202	0.1677	10	9	1.4639
RELB	0.7104	0.1184	0.182	6	7	1.4628
KCNJ1	0.2833	0.1416	0.1536	2	2	1.4616
USO1	1.2009	0.1334	0.242	9	9	1.4605
POLR3F	0.7098	0.1183	0.1775	6	7	1.4584
ASB11	0.2188	0.1094	0.1301	2	1	1.4545
SEC11C	1.7556	0.1097	0.1687	16	10	1.453
FANCE	0.7089	0.1181	0.144	6	7	1.4516
DKK4	0.5976	0.1195	0.182	5	6	1.4512
BMP4	1.7549	0.1097	0.1708	16	10	1.4509
CASK	1.7546	0.117	0.2182	15	10	1.45
DRP2	0.3933	0.1311	0.166	3	4	1.4487
ALDH1B1	0.4826	0.1207	0.1719	4	5	1.4446
PHGDH	0.2821	0.1411	0.155	2	2	1.4435
MMP1	1.1969	0.1197	0.1628	10	9	1.4429
HOMER3	0.282	0.141	0.175	2	2	1.442
EIF4A2	1.7518	0.1348	0.2476	13	10	1.4418
MX2	0.7075	0.1415	0.1865	5	7	1.4413
BMPR1A	0.7074	0.1179	0.1816	6	7	1.441
APIP	0.918	0.1312	0.1934	7	8	1.4399
MSRA	0.1442	0.1442	0.1442	1	0	1.4393
CD46	0.2178	0.1089	0.1321	2	1	1.4388
LCLAT1	0.4819	0.1205	0.1612	4	5	1.4375
STEAP3	1.1952	0.1195	0.1688	10	9	1.4352
CACNB3	1.1952	0.1087	0.2032	11	9	1.4349
CNR2	0.2175	0.1088	0.1264	2	1	1.4342
UNC80	0.3403	0.1701	0.1994	2	3	1.4338
AKR7A2	0.2174	0.1087	0.1346	2	1	1.4328

TYRP1	0.4814	0.1605	0.1948	3	5	1.4322
POLR1E	0.9167	0.1146	0.1922	8	8	1.4321
EPOR	0.706	0.1177	0.1466	6	7	1.4304
RPL8	1.7476	0.0971	0.1782	18	10	1.4298
IGFBP2	0.4811	0.1203	0.1553	4	5	1.4294
CNOT3	0.7058	0.1176	0.145	6	7	1.4292
RPA1	7.5774	0.1166	0.1885	65	11	1.4284
MRPS33	0.3917	0.1306	0.1644	3	4	1.4277
PEX1	0.3917	0.1306	0.1543	3	4	1.4276
USP44	0.3395	0.1698	0.1948	2	3	1.4248
PIGG	0.3914	0.1305	0.2006	3	4	1.4242
CDH7	0.3394	0.1131	0.1361	3	3	1.4225
ARFIP2	0.3394	0.1131	0.1511	3	3	1.4225
CAMP	0.1436	0.1436	0.1436	1	0	1.4221
CLEC4D	0.2167	0.1083	0.132	2	1	1.4214
CNN2	0.4802	0.1201	0.1832	4	5	1.4203
GP6	0.4802	0.12	0.1416	4	5	1.4198
SLC36A4	0.391	0.1303	0.1678	3	4	1.4195
DNAH2	0.3391	0.113	0.1479	3	3	1.4194
ADAM8	0.2166	0.1083	0.1546	2	1	1.419
DIAPH1	1.191	0.1083	0.1611	11	9	1.4159
ALOXE3	0.2163	0.1081	0.133	2	1	1.4143
KRT13	0.5931	0.1186	0.1575	5	6	1.4142
CATSPER3	0.2162	0.1081	0.1387	2	1	1.4133
FLOT1	0.9134	0.1142	0.1749	8	8	1.4133
CLCN3	0.4795	0.1199	0.2031	4	5	1.4128
ASPA	0.1432	0.1432	0.1432	1	0	1.4111
GNLY	0.1432	0.1432	0.1432	1	0	1.41
CHST4	0.3383	0.1128	0.1395	3	3	1.4094
CTRB2	0.5924	0.1185	0.1802	5	6	1.4088
ALOX12	0.3381	0.1127	0.1682	3	3	1.4078
CSGALNACT1	0.4789	0.1197	0.1516	4	5	1.4067
IL1RAP	0.9122	0.114	0.1545	8	8	1.4064
CHMP2B	1.7395	0.0966	0.135	18	10	1.4062
FGF1	7.5128	0.1342	0.2676	56	11	1.4054
MED31	0.9118	0.114	0.1533	8	8	1.4036
IL36B	0.143	0.143	0.143	1	0	1.4035
BIN1	0.3378	0.1126	0.1518	3	3	1.4035
CXADR	0.2155	0.1078	0.1193	2	1	1.4028
DNAH8	0.3377	0.1126	0.1418	3	3	1.4024
ROBO3	0.3896	0.1299	0.1668	3	4	1.4012
CDK14	0.1429	0.1429	0.1429	1	0	1.4012
ATP6V1C2	0.9113	0.1139	0.1569	8	8	1.4009
IFIT2	0.5913	0.1183	0.1411	5	6	1.3997
PRLHR	0.2793	0.1396	0.1491	2	2	1.3975
MUC6	1.1868	0.1187	0.166	10	9	1.3974
HDAC11	0.7015	0.1169	0.1649	6	7	1.3971
IGFBP3	0.9105	0.1138	0.1568	8	8	1.3963
GSTO2	0.9103	0.1138	0.1733	8	8	1.3954
ING4	0.2791	0.1396	0.1924	2	2	1.3953
ASH1L	0.215	0.1075	0.1169	2	1	1.395
HNRNPF	1.1862	0.1078	0.1503	11	9	1.3947
PSPN	0.7011	0.1168	0.1982	6	7	1.3942
MED11	0.7011	0.1402	0.1841	5	7	1.394
HTR1D	0.4774	0.1194	0.1402	4	5	1.3926
SCG2	0.3889	0.1296	0.176	3	4	1.3925
CDK13	0.2148	0.1074	0.1121	2	1	1.3907
GAPVD1	0.4772	0.1193	0.152	4	5	1.3905
ARL6IP5	0.3365	0.1122	0.1367	3	3	1.3882
TAF4	0.9091	0.1299	0.2208	7	8	1.388
CALR	1.7332	0.102	0.169	17	10	1.3878
AFF1	0.3363	0.1121	0.1897	3	3	1.3862
SFTPA2	0.9085	0.1298	0.146	7	8	1.3851
ACOT1	0.2144	0.1072	0.136	2	1	1.3844
TRIM72	0.3361	0.168	0.2015	2	3	1.3827
IRF2	1.1835	0.1184	0.2163	10	9	1.3827
LDLRAP1	1.183	0.1075	0.137	11	9	1.3804
IL6R	1.1825	0.1075	0.1478	11	9	1.3779
DDX18	0.2139	0.107	0.1179	2	1	1.3775
POLR3A	0.6987	0.1165	0.1444	6	7	1.3768
LGI1	0.142	0.142	0.142	1	0	1.3756
MAP2K2	7.4288	0.1077	0.1821	69	11	1.3756
ADM2	0.2138	0.1069	0.1524	2	1	1.375
FXYD2	0.6984	0.1164	0.155	6	7	1.3744
TGIF1	0.9067	0.1295	0.218	7	8	1.3743
ASB13	0.3353	0.1118	0.1243	3	3	1.3742
GPRC6A	0.2777	0.1389	0.1638	2	2	1.3729
CMC1	0.3351	0.1117	0.138	3	3	1.371
ACAD8	0.2135	0.1068	0.1258	2	1	1.3707

CLCNKB	0.2134	0.1067	0.1287	2	1	1.3694
AQP5	0.2134	0.1067	0.1268	2	1	1.3681
SLC39A2	0.5873	0.1468	0.1616	4	6	1.3666
CKM	0.3347	0.1116	0.1452	3	3	1.3658
AQR	0.9052	0.1131	0.2146	8	8	1.3657
TUBGCP2	0.9051	0.1293	0.1902	7	8	1.3651
FBXL16	0.2772	0.1386	0.1686	2	2	1.3649
ADCY10	0.213	0.1065	0.1297	2	1	1.3631
MYBPH	0.1415	0.1415	0.1415	1	0	1.3628
ADH5	0.4745	0.1186	0.1538	4	5	1.3628
FXYD7	0.6966	0.1161	0.1515	6	7	1.3612
RAC1	7.3837	0.09	0.2008	82	11	1.3596
CHIT1	0.2128	0.1064	0.1125	2	1	1.3596
FABP3	0.386	0.1287	0.1611	3	4	1.3564
KIF13A	0.2766	0.1383	0.1418	2	2	1.355
EIF4H	0.4736	0.1184	0.1658	4	5	1.3542
EHD3	0.1412	0.1412	0.1412	1	0	1.3538
HOXB4	0.3858	0.1286	0.1647	3	4	1.3536
KIF5A	1.7212	0.1012	0.1704	17	10	1.3531
BTG2	1.1763	0.1176	0.1474	10	9	1.3503
DHX58	0.3334	0.1111	0.1295	3	3	1.3502
IQCE	0.3855	0.1285	0.1729	3	4	1.3498
CARD11	1.176	0.1307	0.2127	9	9	1.349
KIAA0319	0.2762	0.1381	0.1465	2	2	1.3487
CAMK2B	7.3506	0.1564	0.2727	47	11	1.3478
CITED1	0.5849	0.117	0.172	5	6	1.3475
RASGRF1	0.902	0.1289	0.1606	7	8	1.3471
TRAPPC8	0.3331	0.1665	0.2045	2	3	1.3468
ARHGEF26	0.9019	0.1288	0.2291	7	8	1.3464
MC3R	0.6945	0.1389	0.2251	5	7	1.3456
CA4	0.3329	0.111	0.1193	3	3	1.3453
GSG1	0.1409	0.1409	0.1409	1	0	1.3451
AKR1D1	0.9014	0.1127	0.1776	8	8	1.3438
MGAT5	0.9011	0.1126	0.1411	8	8	1.3423
KRT74	0.2758	0.1379	0.1595	2	2	1.3414
CPOX	0.4723	0.1181	0.1335	4	5	1.3414
IGSF1	0.1408	0.1408	0.1408	1	0	1.3413
DLGAP3	0.2757	0.1379	0.1565	2	2	1.3406
CDH15	0.5839	0.1168	0.1767	5	6	1.3391
DPP4	0.9006	0.1287	0.1973	7	8	1.339
NIPAL4	0.1406	0.1406	0.1406	1	0	1.3372
ANXA9	0.2114	0.1057	0.127	2	1	1.337
SLCO1B1	1.7154	0.1559	0.2293	11	10	1.3364
IL2	1.1722	0.1172	0.1682	10	9	1.3319
NID1	0.2752	0.1376	0.1608	2	2	1.3316
PRKD2	0.3839	0.128	0.133	3	4	1.33
EIF4E2	0.3838	0.1279	0.206	3	4	1.3286
BPHL	0.471	0.1178	0.1483	4	5	1.3284
AFG3L2	0.4708	0.1177	0.1356	4	5	1.3262
CYTH2	0.6918	0.1153	0.1836	6	7	1.3259
POFUT2	0.2748	0.1374	0.1521	2	2	1.3258
PTGFR	0.6917	0.1153	0.1709	6	7	1.325
MPP1	0.4705	0.1176	0.1544	4	5	1.3227
DPF3	1.1702	0.1064	0.1612	11	9	1.3226
NPY2R	0.3833	0.1278	0.1562	3	4	1.3213
PLA2G12A	1.1698	0.13	0.195	9	9	1.3209
BLK	0.3309	0.1103	0.1236	3	3	1.3202
CLDN23	0.4702	0.1175	0.1481	4	5	1.3197
SLC6A13	0.6909	0.1382	0.1707	5	7	1.3193
MT3	0.2743	0.1371	0.1754	2	2	1.3171
RPS19BP1	0.3306	0.1653	0.1931	2	3	1.3166
SLC5A4	0.3305	0.1653	0.2023	2	3	1.316
FARS2	0.3827	0.1276	0.2088	3	4	1.3137
KLK13	0.3826	0.1275	0.1372	3	4	1.3128
CSPG5	1.168	0.1062	0.1737	11	9	1.3128
CDC14A	0.4694	0.1173	0.1675	4	5	1.3118
MAMLD1	1.7068	0.0948	0.1543	18	10	1.3115
ACOXL	0.2098	0.1049	0.1269	2	1	1.3115
CTNNB1	7.2448	0.105	0.1998	69	11	1.3102
IMPACT	0.2738	0.1369	0.1434	2	2	1.3092
DCTD	0.2095	0.1048	0.1654	2	1	1.3068
PDCD4	0.6891	0.1149	0.1682	6	7	1.3059
COL6A6	1.1664	0.106	0.1627	11	9	1.3055
PGM1	1.1662	0.1166	0.1597	10	9	1.305
CDC42BPB	0.3296	0.1099	0.1351	3	3	1.3044
VAMP1	0.4686	0.1562	0.1661	3	5	1.3036
CC2D1B	0.3294	0.1098	0.126	3	3	1.3031
DLG5	0.6885	0.1147	0.1865	6	7	1.3012
M6PR	1.1654	0.1059	0.1599	11	9	1.3011

RPL30	1.7031	0.1064	0.2103	16	10	1.3008
OR6A2	0.2732	0.1366	0.1666	2	2	1.3004
HSP90AA1	7.214	0.1127	0.1864	64	11	1.2993
NEDD4	1.1646	0.1294	0.2566	9	9	1.2978
SMAD6	0.6876	0.1375	0.1832	5	7	1.2945
AXIN2	1.701	0.1134	0.161	15	10	1.2945
SNAPC5	0.5781	0.1445	0.1673	4	6	1.2913
NAE1	0.1389	0.1389	0.1389	1	0	1.2907
ARHGAP17	0.687	0.1145	0.148	6	7	1.2906
SLC12A5	0.3284	0.1642	0.174	2	3	1.2899
PPP2R2D	0.5773	0.1443	0.202	4	6	1.2849
STRA6	0.5773	0.1443	0.187	4	6	1.2849
EPHA10	0.3804	0.1268	0.1411	3	4	1.2846
NRCAM	0.3803	0.1268	0.1601	3	4	1.2844
PLA2G2E	0.8911	0.1114	0.1498	8	8	1.2841
POMP	0.2081	0.2081	0.2081	1	1	1.2835
CDH10	0.3278	0.1093	0.1386	3	3	1.2832
LMO2	0.5771	0.1154	0.1842	5	6	1.2829
ZSCAN10	0.2078	0.2078	0.2078	1	1	1.2791
HUS1	1.6956	0.0997	0.1622	17	10	1.279
IL13RA2	0.3799	0.1266	0.1442	3	4	1.2785
IL22RA1	0.2718	0.1359	0.1643	2	2	1.278
CEPT1	0.4658	0.1165	0.1453	4	5	1.2764
NR1I3	0.1384	0.1384	0.1384	1	0	1.2753
TSPAN7	0.8895	0.1483	0.2067	6	8	1.2753
GNG4	7.1444	0.1116	0.1914	64	11	1.2746
DEPDC5	0.3271	0.109	0.1571	3	3	1.2743
SYP	0.327	0.1635	0.1893	2	3	1.274
FGD1	0.6845	0.1141	0.1705	6	7	1.272
KCNK6	0.4653	0.1163	0.1299	4	5	1.2712
CUL1	7.1323	0.0991	0.1777	72	11	1.2703
SLCO2A1	0.8886	0.1481	0.2063	6	8	1.2701
APOF	0.2072	0.1036	0.1122	2	1	1.2692
PIGC	0.3791	0.1264	0.1523	3	4	1.2691
PTPRR	0.2712	0.1356	0.1614	2	2	1.2677
ACOT9	0.207	0.1035	0.1107	2	1	1.2675
INO80C	0.6838	0.114	0.1443	6	7	1.2668
XRCC3	1.1577	0.1286	0.1918	9	9	1.2666
NKX2-2	0.3789	0.1263	0.1594	3	4	1.2663
BACE1	0.138	0.138	0.138	1	0	1.2654
EIF5A2	0.138	0.138	0.138	1	0	1.2653
BRD8	0.2069	0.1034	0.1232	2	1	1.2648
PARVB	0.3788	0.1263	0.1898	3	4	1.2647
CUX1	0.6835	0.1139	0.1544	6	7	1.2645
GLYAT	0.4645	0.1161	0.1291	4	5	1.2634
FGGY	0.138	0.138	0.138	1	0	1.2628
NUDT18	0.3786	0.1262	0.1409	3	4	1.2619
EPHB6	0.4643	0.1161	0.1319	4	5	1.2613
CLCA2	0.326	0.1087	0.152	3	3	1.2611
CAMLG	0.2066	0.1033	0.123	2	1	1.2606
ARHGAP44	0.3259	0.1086	0.2045	3	3	1.2601
MTMR3	0.4642	0.116	0.1312	4	5	1.2597
KRT1	0.8868	0.1267	0.1674	7	8	1.2596
ADORA2B	1.156	0.1051	0.1567	11	9	1.259
ISG20	0.8867	0.1108	0.1564	8	8	1.2587
CMPK2	0.3257	0.1086	0.1678	3	3	1.2581
TESK1	0.464	0.1547	0.2174	3	5	1.2579
NFIC	0.2705	0.1353	0.1752	2	2	1.2575
CTDSP2	0.2061	0.1031	0.13	2	1	1.2526
PTHLH	0.3777	0.1259	0.1701	3	4	1.2511

Table S6. Pathway-level influence with adjustment for set size, for all 1,792 retained pathways.

Pathway	Summed influence	Mean influence per gene	Member genes	Influence z
REACTOME_PASSIVE_TRANSPORT_BY_AQUAPORINS	1.9252	0.175	11	4.3915
REACTOME_FATTY_ACIDS	2.099	0.1499	14	3.36
REACTOME_MISCELLANEOUS_SUBSTRATES	2.0529	0.1711	12	3.1744
REACTOME_FORMATION_OF_THE_CORNIFIED_ENVELOPE	9.6643	0.1124	86	3.1505
KEGG_MEDICUS_REFERENCE_CXCR4_GNAQ_PLCB_G_CALCINEURIN_SIGNALING_PATHWAY	2.5705	0.1353	19	3.1487

REACTOME_ACETYLCHOLINE_BINDING_AND_DOWNSTREAM_EVENTS	1.6508	0.1376	12	3.0626
KEGG_MEDICUS_REFERENCE_GF_RTK_RAS_ERK_SIGNALING_PATHWAY	9.5559	0.1098	87	3.0604
REACTOME_METABOLISM_OF_AMINE_DERIVED_HORMONES	3.0624	0.1392	22	2.8922
REACTOME_DEFECTIVE_EXT2_CAUSES_EXOSTOSES_2	1.9766	0.1412	14	2.8676
REACTOME_RETROGRADE_TRANSPORT_AT_THE_TRANS_GOLGI_NETWORK	5.3992	0.1149	47	2.8366
REACTOME_EICOSANOIDS	1.9666	0.1639	12	2.8274
REACTOME_DOPAMINE_NEUROTRANSMITTER_RELEASE_CYCLE	3.0086	0.1433	21	2.7433
REACTOME_TRANSLATION	23.9562	0.0799	300	2.742
REACTOME_NEPHRON_DEVELOPMENT	1.9244	0.1604	12	2.6577
REACTOME_TRANSMISSION_ACROSS_CHEMICAL_SYNAPSES	23.4886	0.0947	248	2.6266
REACTOME_MITOCHONDRIAL_FATTY_ACID_BETA_OXIDATION	3.805	0.1227	31	2.5983
REACTOME_SLC_MEDIATED_TRANSPORT_OF_NEUROTRANSMITTERS	2.3951	0.1331	18	2.5877
KEGG_MEDICUS_REFERENCE_IFN_RIPK1_3_SIGNALING_PATHWAY	2.3748	0.125	19	2.523
REACTOME_METABOLISM_OF_CARBOHYDRATES_AND_CARBOHYDRATE_DERIVATIVES	23.0219	0.0846	272	2.5115
REACTOME_TRAFFICKING_OF_GLUR2_CONTAINING_AMPA_RECEPTORS	2.3675	0.1393	17	2.4996
REACTOME_DEVELOPMENTAL_LINEAGE_OF_MULTIPOTENT_PANCREATIC_PROGENITOR_CELLS	2.3657	0.1314	18	2.4938
REACTOME_SARS_COV_2_INFECTION	22.8588	0.0859	266	2.4713
REACTOME_METABOLISM_OF_FAT_SOLUBLE_VITAMINS	5.1612	0.1173	44	2.4464
REACTOME_SLC_MEDIATED_TRANSMEMBRANE_TRANSPORT	22.7319	0.1019	223	2.44
REACTOME_GLUCAGON_SIGNALING_IN_METABOLIC_REGULATION	3.71	0.1124	33	2.3732
REACTOME_METABOLISM_OF_PORPHYRINS	2.866	0.1194	24	2.3492
REACTOME_NEUROTRANSMITTER_RELEASE_CYCLE	5.0998	0.1109	46	2.3458
KEGG_MEDICUS_REFERENCE_ARNO_ARF_ACTB_G_SIGNALING_PATHWAY	2.8634	0.1145	25	2.3419
KEGG_MEDICUS_REFERENCE_ANTIGEN_PROCESSING_AND_PRESENTATION_BY_MHC_CLASS_I_MOLECULES	1.5008	0.1251	12	2.3357
REACTOME_ASPARAGINE_N_LINKED_GLYCOSYLATION	22.2313	0.0777	286	2.3165
REACTOME_PROCESSING_OF_CAPPED_INTRON_CONTAINING_PRE_MRNA	22.1967	0.0841	264	2.308
REACTOME_COBALAMIN_CBL_VITAMIN_B12_TRANSPORT_AND_METABOLISM	2.3028	0.1279	18	2.2925
REACTOME_ACTIVATION_OF_THE_PHOTOTRANSDUCTION_CASCADE	1.4917	0.1492	10	2.2914
KEGG_MEDICUS_VARIANT_NOTCH_OVEREXPRESSION_TO_NOTCH_SIGNALING_PATHWAY	2.2902	0.1431	16	2.2523
KEGG_MEDICUS_REFERENCE_IL1_IL1R_JNK_SIGNALING_PATHWAY	2.2854	0.1203	19	2.2371
KEGG_MEDICUS_REFERENCE_GPI_ANCHOR_BIOSYNTHESIS	2.8156	0.1224	23	2.2099
REACTOME_TNFR1_INDUCED_NF_KAPPA_B_SIGNALING_PATHWAY	3.6348	0.1136	32	2.1949
KEGG_MEDICUS_REFERENCE_TRH_TRHR_PLCB_PKC_SIGNALING_PATHWAY	1.4703	0.1225	12	2.1878
HALLMARK_IL6_JAK_STAT3_SIGNALING	8.4973	0.0988	86	2.1806
REACTOME_RSV_HOST_INTERACTIONS	8.4935	0.1075	79	2.1775
KEGG_MEDICUS_REFERENCE_INTRINSIC_APOPTOTIC_PATHWAY	1.4655	0.1221	12	2.1646
REACTOME_AMINE_LIGAND_BINDING_RECEPTORS	3.6208	0.1168	31	2.1618
REACTOME_CLEC7A_DECTIN_1_SIGNALING	8.4684	0.102	83	2.1566
KEGG_MEDICUS_REFERENCE_GNRH_GNRHR_PLCB_PKC_SIGNALING_PATHWAY	2.258	0.1188	19	2.1494
KEGG_MEDICUS_REFERENCE_REGULATION_OF_GF_RTK_RAS_ERK_SIGNALING_PTP	2.2576	0.1188	19	2.1482
REACTOME_HCMV_LATE_EVENTS	4.976	0.105	47	2.142

	1	9		9
REACTOME_OLFACTORY_SIGNALING_PATHWAY	3.6064	0.1127	32	2.1277
KEGG_MEDICUS_PATHOGEN_ESCHERICHIA_EAE_TIR_TCCP_TO_ACTIN_SIGNALING_PATHWAY	1.781	0.1272	14	2.0808
KEGG_MEDICUS_REFERENCE_PTH_PTH1R_PKA_SIGNALING_PATHWAY	1.781	0.1272	14	2.0807
REACTOME_HS_GAG_BIOSYNTHESIS	3.5846	0.1086	33	2.076
KEGG_MEDICUS_VARIANT_MLL_AF4_FUSION_TO_TRANSCRIPTIONAL_ACTIVATION	2.7665	0.1153	24	2.0742
REACTOME_ANTIGEN_PROCESSING_UBIQUITINATION_PROTEASOME_DEGRADATION	21.2079	0.0783	271	2.064
REACTOME_ESTROGEN_DEPENDENT_GENE_EXPRESSION	8.3565	0.0983	85	2.0636
REACTOME_ANTIGEN_PRESENTATION_FOLDING_ASSEMBLY_AND_PEPTIDE_LOADING_OF_CLASS_I_MHC	3.5731	0.1232	29	2.0486
REACTOME_CLASS_A_1_RHODOPSIN_LIKE_RECEPTORS	21.0952	0.0796	265	2.0362
REACTOME_IRS_MEDIATED_SIGNALLING	4.9044	0.1043	47	2.0253
KEGG_MEDICUS_PATHOGEN_SALMONELLA_SOPB_TO_ARNO_ARF_ACTB_G_SIGNALING_PATHWAY	3.5599	0.1187	30	2.0175
KEGG_MEDICUS_VARIANT_MUTATION_CAUSED_ABERRANT_HTT_TO_RETROGRADE_AXONAL_TRANSPORT	4.8942	0.1165	42	2.0086
KEGG_MEDICUS_REFERENCE_TLR2_4_MAPK_SIGNALING_PATHWAY	2.7426	0.1097	25	2.008
KEGG_MEDICUS_REFERENCE_ASSEMBLY_AND_TRAFFICKING_OF_TELOMERASE	1.7622	0.1175	15	2.0054
KEGG_MEDICUS_REFERENCE_AUTOPHAGOSOME_AND_LYSOSOME_FUSION_TETHERING_FACTOR	1.761	0.1174	15	2.0004
KEGG_MEDICUS_REFERENCE_MGLUR1_TRPC3_SIGNALING_PATHWAY	1.7601	0.1354	13	1.997
REACTOME_DNA_REPAIR	20.8713	0.0797	262	1.981
KEGG_MEDICUS_VARIANT_MLL_ENL_FUSION_TO_TRANSCRIPTIONAL_ACTIVATION	1.4227	0.1293	11	1.9574
REACTOME_DISEASES_OF_IMMUNE_SYSTEM	3.5326	0.1104	32	1.9526
KEGG_MEDICUS_REFERENCE_TLR1_2_4_NFKB_SIGNALING_PATHWAY	2.722	0.1361	20	1.951
REACTOME_TIE2_SIGNALING	2.195	0.1219	18	1.9479
REACTOME_CLASS_C_3_METABOTROPIC_GLUTAMATE_PHEROMONE_RECEPTORS	2.7205	0.1183	23	1.947
KEGG_MEDICUS_REFERENCE_TSH_DUOX2_TG_SIGNALING_PATHWAY	1.42	0.1183	12	1.9446
REACTOME_SIGNALING_BY_NODAL	2.1924	0.1218	18	1.9395
REACTOME_CELL_CELL_COMMUNICATION	20.6852	0.0837	247	1.9351
KEGG_MEDICUS_REFERENCE_HOMOLOGOUS_RECOMBINATION	3.5252	0.1102	32	1.935
REACTOME_REGULATION_OF_PLK1_ACTIVITY_AT_G2_M_TRANSITION	8.1942	0.0987	83	1.9287
REACTOME_TNFR2_NON_CANONICAL_NF_KB_PATHWAY	8.1889	0.0975	84	1.9243
REACTOME_DAG_AND_IP3_SIGNALING	4.8354	0.1209	40	1.9122
REACTOME_ASSOCIATION_OF_TRIC_CCT_WITH_TARGET_PROTEINS_DURING_BIOSYNTHESIS	3.5107	0.1097	32	1.9009
REACTOME_G_ALPHA_I_SIGNALLING_EVENTS	20.538	0.0761	270	1.8988
REACTOME_DISEASES_OF_METABOLISM	20.5157	0.0834	246	1.8933
REACTOME_METABOLISM_OF_NUCLEOTIDES	8.1475	0.0994	82	1.8899
REACTOME_ASSEMBLY_OF_ACTIVE_LPL_AND_LIPC_LIPASE_COMPLEXES	2.176	0.128	17	1.887
REACTOME_MITOTIC_PROPHASE	8.128	0.1069	76	1.8737
KEGG_MEDICUS_REFERENCE_CARDIAC_TYPE_VGCC_RYR_SIGNALING	2.1716	0.1143	19	1.873
REACTOME_TP53_REGULATES_TRANSCRIPTION_OF_CASPASE_ACTIVATORS_AND_CASPASES	1.4046	0.1277	11	1.8697
KEGG_MEDICUS_REFERENCE_CYTOKINE_JAK_STAT_SIGNALING_PATHWAY	4.7988	0.1116	43	1.8522
REACTOME_INTERLEUKIN_2_FAMILY_SIGNALING	4.7886	0.114	42	1.8354
REACTOME_FORMATION_OF_THE_BETA_CATENIN_TCF_TRANSACTIVATING_COMPLEX	3.4829	0.1088	32	1.8349
REACTOME_TRANSFERRIN_ENDOCYTOSIS_AND_RECYCLING	3.482	0.124	28	1.833

	3	4		6
REACTOME_SENSORY_PERCEPTION	20.2643	0.0947	214	1.8313
REACTOME_EPIGENETIC_REGULATION_OF_GENE_EXPRESSION_BY_MLL3_AND_MLL4_COMPLEXES	8.0759	0.1122	72	1.8304
REACTOME_NOTCH3_ACTIVATION_AND_TRANSMISSION_OF_SIGNAL_TO_THE_NUCLEUS	2.6758	0.1115	24	1.8234
REACTOME_RIPK1_MEDIATED_REGULATED_NECROSIS	3.4778	0.1122	31	1.8228
REACTOME_PARACETAMOL_ADME	2.6752	0.1216	22	1.8217
KEGG_MEDICUS_VARIANT_MUTATION_CAUSED_ABERRANT_HTT_TO_MGLUR5_CA2_APOPTOTIC_PATHWAY	2.6741	0.1215	22	1.8186
REACTOME_CELL_EXTRACELLULAR_MATRIX_INTERACTIONS	2.1508	0.1195	18	1.8066
KEGG_MEDICUS_PATHOGEN_HIV_GP120_TO_TNF_NFKB_SIGNALING_PATHWAY	1.7119	0.1141	15	1.8031
REACTOME_ALPHA_PROTEIN_KINASE_1_SIGNALING_PATHWAY	1.3906	0.1264	11	1.8019
KEGG_MEDICUS_REFERENCE_ACTIVATION_OF_PRC2.2_BY_UBIQUITINATION_OF_H2AK119_IN_GERMLINE_GENES	2.6677	0.1067	25	1.8008
REACTOME_HEME_SIGNALING	4.7658	0.1059	45	1.798
KEGG_MEDICUS_PATHOGEN_HCV_CORE_TO_ERK_SIGNALING_PATHWAY	1.7084	0.1314	13	1.789
REACTOME_MAPK_FAMILY_SIGNALING_CASCADES	20.0792	0.0685	293	1.7857
REACTOME_GASTRIN_CREB_SIGNALLING_PATHWAY_VIA_PKC_AND_MAPK	2.1434	0.1191	18	1.7828
REACTOME_NCAM1_INTERACTIONS	4.7559	0.116	41	1.7818
REACTOME_SHC1_EVENTS_IN_ERBB2_SIGNALING	2.6607	0.1209	22	1.7815
REACTOME_SIGNALING_BY_FGFR	8.0138	0.0943	85	1.7788
REACTOME_REGULATION_OF_GENE_EXPRESSION_IN_BETA_CELLS	2.1385	0.1188	18	1.7672
REACTOME_REPRODUCTION	7.9892	0.0963	83	1.7583
REACTOME_GLUCAGON_LIKE_PEPTIDE_1_GLP1_REGULATES_INSULIN_SECRETION	4.7332	0.1127	42	1.7446
REACTOME_TRANSCRIPTIONAL_REGULATION_BY_RUNX3	7.9681	0.0972	82	1.7408
REACTOME_SIGNALING_BY_ACTIVATED_POINT_MUTANTS_OF_FGFR1	1.3777	0.1378	10	1.7395
REACTOME_PEPTIDE_HORMONE_METABOLISM	7.9553	0.1075	74	1.7302
REACTOME_THYROXINE_BIOSYNTHESIS	1.6839	0.1203	14	1.6903
REACTOME_CHROMOSOME_MAINTENANCE	7.9065	0.0953	83	1.6896
REACTOME_MAPK1_MAPK3_SIGNALING	19.6626	0.0762	258	1.6829
REACTOME_SYNTHESIS_SECRETION_AND_INACTIVATION_OF_GLUCOSE_DEPENDENT_INSULINOTROPIC_POLYPEPTIDE_GIP	1.3655	0.1241	11	1.6803
REACTOME_GROWTH_HORMONE_RECEPTOR_SIGNALING	2.6225	0.1249	21	1.6758
REACTOME_ANTIMICROBIAL_PEPTIDES	4.6907	0.1066	44	1.675
HALLMARK_NOTCH_SIGNALING	3.4151	0.1102	31	1.6743
REACTOME_ORGANELLE_BIOGENESIS_AND_MAINTENANCE	19.5587	0.0818	239	1.6573
REACTOME_SIGNALING_BY_WNT_IN_CANCER	3.4075	0.1099	31	1.6562
REACTOME_POST_TRANSLATIONAL_MODIFICATION_SYNTHESIS_OF_GPI_ANCHORED_PROTEINS	7.8559	0.102	77	1.6475
KEGG_MEDICUS_REFERENCE_ELECTRON_TRANSFER_IN_COMPLEX_IV	1.6681	0.1112	15	1.6267
REACTOME_INTERFERON_SIGNALING	19.4204	0.0796	244	1.6232
REACTOME_ORGANIC_ANION_TRANSPORT_BY_SLCO_TRANSPORTERS	1.3535	0.1354	10	1.6223
REACTOME_CELL_JUNCTION_ORGANIZATION	19.3695	0.0905	214	1.6106
REACTOME_BBSOME_MEDIATED_CARGO_TARGETING_TO_CILIUM	2.5947	0.1236	21	1.5992
REACTOME_PHASE_2_PLATEAU_PHASE	1.6608	0.1278	13	1.5975
KEGG_MEDICUS_REFERENCE_CX3CR1_GNAI_AC_PKA_SIGNALING_PATHWAY	2.0851	0.1227	17	1.5965

KEGG_MEDICUS_PATHOGEN_HPV_E5_TO_EGFR_RAS_ERK_SIGNALING_PATHWAY	1.6545	0.1103	15	1.5723
REACTOME_PLATELET_ACTIVATION_SIGNALING_AND_AGGREGATION	19.1664	0.0764	251	1.5605
KEGG_MEDICUS_REFERENCE_RETROGRADE_AXONAL_TRANSPORT	4.6161	0.1154	40	1.5526
REACTOME_RETROGRADE_NEUROTROPHIN_SIGNALLING	1.6492	0.1178	14	1.551
REACTOME_FANCONI_ANEMIA_PATHWAY	3.3628	0.1019	33	1.5501
KEGG_MEDICUS_REFERENCE_ATR_SIGNALING	1.3363	0.1114	12	1.539
REACTOME_INTERLEUKIN_7_SIGNALING	2.572	0.1169	22	1.5364
REACTOME_PENTOSE_PHOSPHATE_PATHWAY	1.6426	0.1173	14	1.5242
REACTOME_DISEASES_OF_PROGRAMMED_CELL_DEATH	4.5965	0.1069	43	1.5204
REACTOME_FCGAMMA_RECEPTOR_FCGR_DEPENDENT_PHAGOCYTOSIS	7.7002	0.0928	83	1.5182
REACTOME_INTERLEUKIN_20_FAMILY_SIGNALING	2.5614	0.1164	22	1.507
REACTOME_RRNA_PROCESSING_IN_THE_MITOCHONDRION	1.3297	0.133	10	1.5067
REACTOME_FGFR1_LIGAND_BINDING_AND_ACTIVATION	2.0561	0.1285	16	1.5036
REACTOME_HDACS_DEACETYLATE_HISTONES	3.3398	0.1044	32	1.4956
REACTOME_G_ALPHA_Q_SIGNALLING_EVENTS	18.8954	0.0969	195	1.4937
REACTOME_INTERLEUKIN_3_INTERLEUKIN_5_AND_GM_CSF_SIGNALING	4.577	0.0974	47	1.4885
REACTOME_RHO_GTPASE_EFFECTORS	18.8733	0.0767	246	1.4882
KEGG_MEDICUS_PATHOGEN_KSHV_VGPCR_TO_GNB_G_ERK_SIGNALING_PATHWAY	3.3335	0.1075	31	1.4808
KEGG_MEDICUS_VARIANT_EML4_ALK_FUSION_KINASE_TO_RAS_ERK_SIGNALING_PATHWAY	1.324	0.1103	12	1.4795
REACTOME_GLYCOGEN_METABOLISM	2.5502	0.1063	24	1.476
REACTOME_RECRUITMENT_OF_NUMA_TO_MITOTIC_CENTROSOMES	7.6483	0.0889	86	1.4751
REACTOME_METABOLIC_DISORDERS_OF_BIOLOGICAL_OXIDATION_ENZYMES	3.3294	0.1233	27	1.471
KEGG_MEDICUS_REFERENCE_CA2_CAM_CAMK_SIGNALING_PATHWAY	2.0454	0.1136	18	1.4695
KEGG_MEDICUS_REFERENCE_REGULATION_OF_GF_RTK_RAS_ERK_SIGNALING_UBIQUITINATION_OF_RTK_BY_CBL	2.5456	0.1018	25	1.4634
REACTOME_RESPIRATORY_SYNCYTIAL_VIRUS_RSV_ATTACHMENT_AND_ENTRY	2.5453	0.1157	22	1.4624
KEGG_MEDICUS_REFERENCE_GF_RTK_RAS_PI3K_SIGNALING_PATHWAY	7.6303	0.0908	84	1.4601
KEGG_MEDICUS_VARIANT_DELETED_DMD_TO_DYSTROPHIN_ASSOCIATED_PROTEIN_COMPLEX	1.3197	0.132	10	1.4584
REACTOME_TRANSCRIPTIONAL_REGULATION_OF_WHITE_ADIPOCYTE_DIFFERENTIATION	7.628	0.0954	80	1.4582
REACTOME_COLLAGEN_FORMATION	7.6215	0.0929	82	1.4528
REACTOME_RESPONSE_OF_EIF2AK1_HRI_TO_HEME_DEFICIENCY	1.6225	0.1159	14	1.4435
KEGG_MEDICUS_REFERENCE_CROSSTALK_BETWEEN_EXTRINSIC_AND_INTRINSIC_APOPTOTIC_PATHWAYS	1.6225	0.1082	15	1.4435
REACTOME_EXPRESSION_OF_BMAL_ARNTL_CLOCK_AND_NPAS2	3.3178	0.1005	33	1.4435
KEGG_MEDICUS_ENV_FACTOR_TCDD_TO_AHR_SIGNALING_PATHWAY	2.0363	0.1198	17	1.4404
REACTOME_BASIGIN_INTERACTIONS	2.5356	0.1056	24	1.4357
REACTOME_CONSTITUTIVE_SIGNALING_BY_ABERRANT_PI3K_IN_CANCER	7.5995	0.0916	83	1.4345
REACTOME_SYNTHESIS_OF_BILE_ACIDS_AND_BILE_SALTS_VIA_27_HYDROXYCHOLESTEROL	1.6189	0.1245	13	1.4289
KEGG_MEDICUS_REFERENCE_MACHR_CA2_APOPTOTIC_PATHWAY	2.032	0.1069	19	1.4265
REACTOME_SHC1_EVENTS_IN_ERBB4_SIGNALING	1.6169	0.1155	14	1.421
REACTOME_GBP_MEDIATED_HOST_DEFENSE	1.6153	0.1154	14	1.4146
REACTOME_DECTIN_2_FAMILY	2.5274	0.1053	24	1.4131
REACTOME_INTRACELLULAR_SIGNALING_BY_SECOND_MESSENGERS	18.56	0.064	287	1.412

	56	7		3
REACTOME_N_GLYCAN_ANTENNAE_ELONGATION_IN_THE_MEDIAL_TRANS_GOLGI	2.5239	0.1097	23	1.4035
HALLMARK_P53_PATHWAY	18.515	0.0974	190	1.3998
KEGG_MEDICUS_PATHOGEN_HPV_E6_TO_NOTCH_SIGNALING_PATHWAY_N00381	1.3074	0.1089	12	1.3987
REACTOME_SIGNALING_BY_WNT	18.4783	0.0757	244	1.3908
REACTOME_SIGNALING_BY_NUCLEAR_RECEPTORS	18.4546	0.0828	223	1.3849
REACTOME_RAB_GEFS_EXCHANGE_GTP_FOR_GDP_ON_RABS	7.5382	0.0954	79	1.3836
REACTOME_NEUROTRANSMITTER_RECEPTORS_AND_POSTSYNAPTIC_SIGNAL_TRANSMISSION	18.4455	0.0976	189	1.3827
REACTOME_NEGATIVE_REGULATION_OF_FGFR1_SIGNALING	3.2888	0.1061	31	1.3749
REACTOME_ACTIVATED_NOTCH1_TRANSMITS_SIGNAL_TO_THE_NUCLEUS	3.2879	0.1134	29	1.3727
REACTOME_ASPARTATE_AND_ASPARAGINE_METABOLISM	1.3018	0.1302	10	1.3719
KEGG_MEDICUS_REFERENCE_LHCGR_GNAS_PKA_SIGNALING_PATHWAY	1.6029	0.1145	14	1.3648
REACTOME_CONDENSATION_OF_PROPHASE_CHROMOSOMES	1.6026	0.1336	12	1.3636
REACTOME_KSRP_KHSRP_BINDS_AND_DESTABILIZES_MRNA	2.0119	0.1183	17	1.3622
REACTOME_FATTY_ACYL_COA_BIOSYNTHESIS	3.2821	0.1172	28	1.359
KEGG_MEDICUS_ENV_FACTOR_METALS_TO_NFKB_SIGNALING_PATHWAY	1.5996	0.1066	15	1.3514
KEGG_MEDICUS_PATHOGEN_SHIGELLA_IPGD_TO_ARNO_ARF_ACTB_G_SIGNALING_PATHWAY	3.2785	0.1131	29	1.3505
REACTOME_HEPARAN_SULFATE_HEPARIN_HS_GAG_METABOLISM	4.4909	0.1123	40	1.3474
REACTOME_PROTEIN_PROTEIN_INTERACTIONS_AT_SYNAPSES	7.489	0.0913	82	1.3427
REACTOME_SELECTIVE_AUTOPHAGY	7.4879	0.0972	77	1.3418
REACTOME_TELOMERE_EXTENSION_BY_TELOMERASE	2.5001	0.1136	22	1.3375
REACTOME_APOPTOTIC_EXECUTION_PHASE	4.4844	0.1019	44	1.3366
KEGG_MEDICUS_REFERENCE_WNT_SIGNALING_MODULATION_WNT_INHIBITOR	2.4994	0.1087	23	1.3356
REACTOME_AMINO_ACIDS_REGULATE_MTORC1	4.4834	0.0954	47	1.3351
KEGG_MEDICUS_PATHOGEN_SALMONELLA_SIFA_TO_MICROTUBULE_PLUS_END_DIRECTED_TRANSPORT	2.4989	0.1136	22	1.3343
REACTOME_REGULATION_OF_LOCALIZATION_OF_FOXO_TRANSCRIPTION_FACTORS	1.2931	0.1176	11	1.3296
REACTOME_ACYL_CHAIN_REMODELLING_OF_PG	1.9993	0.125	16	1.3219
KEGG_MEDICUS_PATHOGEN_HTLV_1_TAX_TO_NFY_MEDIATED_TRANSCRIPTION	1.2908	0.1173	11	1.3184
REACTOME_NUCLEAR_EVENTS_MEDIATED_BY_NFE2L2	7.4573	0.0898	83	1.3163
REACTOME_PLASMA_LIPOPROTEIN_ASSEMBLY_REMODELING_AND_CLEARANCE	7.4559	0.1036	72	1.3152
REACTOME_THE_ACTIVATION_OF_ARYLSULFATASES	1.2899	0.1173	11	1.3141
REACTOME_BIOSYNTHESIS_OF_SPECIALIZED_PRORESOLVING_MEDIATORS_SPMS	1.9967	0.1175	17	1.3135
REACTOME_DENGUE_VIRUS_GENOME_TRANSLATION_AND_REPLICATION	7.4489	0.0993	75	1.3093
REACTOME_TP53_REGULATES_TRANSCRIPTION_OF_CELL_CYCLE_GENES	4.4677	0.1015	44	1.3093
REACTOME_BETA_OXIDATION_OF_VERY_LONG_CHAIN_FATTY_ACIDS	1.2887	0.1172	11	1.3085
REACTOME_RAC2_GTPASE_CYCLE	7.4465	0.0866	86	1.3073
REACTOME_DOWNSTREAM_TCR_SIGNALING	7.4419	0.0966	77	1.3035
REACTOME_NUCLEAR_PORE_COMPLEX_NPC_DISASSEMBLY	3.2574	0.1018	32	1.3004
REACTOME_ATTACHMENT_AND_ENTRY	1.9917	0.1107	18	1.2978
REACTOME_ABERRANT_REGULATION_OF_MITOTIC_G1_S_TRANSITION_IN_CANCER_DUE_TO_RB1_DEFECTS	1.9913	0.1171	17	1.2963
REACTOME_RECYCLING_PATHWAY_OF_L1	4.4531	0.106	42	1.2854

REACTOME_ADRENALINE_NORADRENALINE_INHIBITS_INSULIN_SECRETION	3.249	0.116	28	1.2806
REACTOME_REGULATION_OF_PD_L1_CD274_POST_TRANSLATIONAL_MODIFICATION	7.4117	0.0893	83	1.2784
REACTOME_EPIGENETIC_REGULATION_OF_GENE_EXPRESSION	17.999	0.0756	238	1.2725
REACTOME_KETONE_BODY_METABOLISM	1.2801	0.128	10	1.2664
REACTOME_BINDING_AND_UPTAKE_OF_LIGANDS_BY_SCAVENGER_RECEPTORS	4.4414	0.1009	44	1.2662
KEGG_MEDICUS_VARIANT_MUTATION_CAUSED_ABERRANT_SNCA_TO_ANTEROGRADE_AXONAL_TRANSPORT	2.4734	0.1124	22	1.2637
REACTOME_GENE_AND_PROTEIN_EXPRESSION_BY_JAK_STAT_SIGNALING_AFTER_INTERLEUKIN_12_STIMULATION	3.2399	0.1012	32	1.2589
REACTOME_MUCOPOLYSACCHARIDOSES	1.278	0.1278	10	1.2565
KEGG_MEDICUS_REFERENCE_KEAP1_NRF2_SIGNALING_PATHWAY	1.9784	0.1099	18	1.255
REACTOME_CELL_CYCLE_CHECKPOINTS	17.9191	0.0756	237	1.2529
REACTOME_DEADENYLATION_OF_MRNA	2.467	0.1298	19	1.2461
HALLMARK_INTERFERON_GAMMA_RESPONSE	17.8749	0.0941	190	1.2419
KEGG_MEDICUS_REFERENCE_IL2_FAMILY_TO_JAK_STAT_SIGNALING_PATHWAY	2.4639	0.112	22	1.2374
REACTOME_PLASMA_LIPOPROTEIN_REMODELING	3.2261	0.1008	32	1.2264
REACTOME_NUCLEOTIDE_LIKE_PURINERGIC_RECEPTORS	1.5659	0.1118	14	1.2157
REACTOME_PHOSPHOLIPID_METABOLISM	17.7413	0.0959	185	1.209
KEGG_MEDICUS_ENV_FACTOR_BENZO_A_PYRENRE_TO_CYP_MEDIATED_METABOLISM	1.2682	0.1268	10	1.2088
KEGG_MEDICUS_REFERENCE_HORMONE_LIKE_CYTOKINE_TO_JAK_STAT_SIGNALING_PATHWAY	1.5634	0.1042	15	1.2056
KEGG_MEDICUS_REFERENCE_HGF_MET_RAS_ERK_SIGNALING_PATHWAY	1.5609	0.1041	15	1.1955
KEGG_MEDICUS_REFERENCE_HEDGEHOG_SIGNALING_PATHWAY	1.5608	0.1115	14	1.1953
KEGG_MEDICUS_REFERENCE_DEUBIQUITINATION_OF_H2AK119	2.4473	0.0979	25	1.1915
REACTOME_FRS_MEDIATED_FGFR4_SIGNALING	2.447	0.1165	21	1.1907
KEGG_MEDICUS_REFERENCE_PROMOTION_OF_MICROTUBULE_GROWTH	1.958	0.103	19	1.1897
REACTOME_DEFECTIVE_B4GALT7_CAUSES_EDS_PROGEROID_TYPE	2.4447	0.1222	20	1.1844
REACTOME_PLATELET_HOMEOSTASIS	7.2982	0.0879	83	1.1841
HALLMARK_ADIPOGENESIS	17.6369	0.0959	184	1.1832
REACTOME_METAL_ION_SLC_TRANSPORTERS	4.3908	0.1071	41	1.1832
KEGG_MEDICUS_REFERENCE_WNT_SIGNALING_MODULATION_WNT_ACYLATION	1.9554	0.1222	16	1.1817
REACTOME_SIGNALING_BY_MODERATE_KINASE_ACTIVITY_BRAF_MUTANTS	4.3883	0.0997	44	1.1792
REACTOME_HIV_TRANSCRIPTION_INITIATION	4.388	0.0954	46	1.1787
REACTOME_VITAMIN_D_CALCIFEROL_METABOLISM	1.2617	0.1147	11	1.1776
REACTOME_CYCLIN_D_ASSOCIATED_EVENTS_IN_G1	4.3852	0.0933	47	1.174
KEGG_MEDICUS_REFERENCE_BMP_SIGNALING_PATHWAY	1.953	0.1028	19	1.174
REACTOME_DEUBIQUITINATION	17.5992	0.0822	214	1.1739
HALLMARK_KRAS_SIGNALING_DN	17.5953	0.1011	174	1.173
REACTOME_CLASS_B_2_SECRETIN_FAMILY_RECEPTORS	7.2784	0.0921	79	1.1676
REACTOME_ADP_SIGNALLING_THROUGH_P2Y_PURINOCEPTOR_12	2.4368	0.1108	22	1.1625
REACTOME_ZINC_TRANSPORTERS	1.9483	0.1299	15	1.1589
REACTOME_SIGNALING_BY_CSF1_M_CSF_IN_MYELOID_CELLS	3.1948	0.1031	31	1.1521
REACTOME_NEDDYLATION	17.4979	0.081	216	1.149
KEGG_MEDICUS_PATHOGEN_HPV_E6_TO_NOTCH_SIGNALING_PATHWAY_N00380	1.255	0.104	12	1.147

	5	6		3
REACTOME_CALNEXIN_CALRETICULIN_CYCLE	2.4305	0.1013	24	1.145
REACTOME_RHOF_GTPASE_CYCLE	4.3651	0.1091	40	1.1411
REACTOME_RESOLUTION_OF_D_LOOP_STRUCTURES	3.1894	0.0997	32	1.1394
REACTOME_NEGATIVE_REGULATION_OF_TCF_DEPENDENT_SIGNALING_BY_WNT_LIGAND_ANTAGONISTS	1.5463	0.1104	14	1.1369
HALLMARK_ESTROGEN_RESPONSE_LATE	17.4034	0.0911	191	1.1256
REACTOME_MET_ACTIVATES_RAS_SIGNALING	1.2507	0.1137	11	1.1244
REACTOME_FBXL7_DOWN_REGULATES_AURKA_DURING_MITOTIC_ENTRY_AND_IN_EARLY_MITOSIS	4.3547	0.1089	40	1.1241
REACTOME_ACYL_CHAIN_REMODELLING_OF_PC	2.4227	0.1053	23	1.1236
REACTOME_ADORA2B_MEDIATED_ANTI_INFLAMMATORY_CYTOKINES_PRODUCTION	4.3517	0.1012	43	1.1191
REACTOME_TRISTETRAPROLIN_TTP_ZFP36_BINDS_AND_DESTABILIZES_MRNA	1.9343	0.1138	17	1.114
KEGG_MEDICUS_REFERENCE_FSHR_GNAS_PKA_SIGNALING_PATHWAY	1.54	0.11	14	1.1116
REACTOME_N_GLYCAN_ANTENNAE_ELONGATION	1.5399	0.1185	13	1.1114
REACTOME_BASE_EXCISION_REPAIR	4.343	0.101	43	1.1049
REACTOME_HIV_INFECTION	17.3169	0.0866	200	1.1043
REACTOME_REGULATION_OF_T_CELL_ACTIVATION_BY_CD28_FAMILY	17.2974	0.0896	193	1.0995
KEGG_MEDICUS_REFERENCE_MITOCHONDRIAL_COMPLEX_UCP1_IN_THERMOGENESIS	4.337	0.0943	46	1.095
KEGG_MEDICUS_REFERENCE_TESTOSTERONE_BIOSYNTHESIS	1.2438	0.1244	10	1.0906
REACTOME_VXPX_CARGO_TARGETING_TO_CILIUM	1.9269	0.107	18	1.0904
REACTOME_SARS_COV_2_MODULATES_AUTOPHAGY	1.2435	0.113	11	1.0892
REACTOME_SIGNALING_BY_MET	7.1837	0.0921	78	1.0889
REACTOME_GP1B_IX_V_ACTIVATION_SIGNALLING	1.2428	0.113	11	1.086
KEGG_MEDICUS_REFERENCE_NAD_BIOSYNTHESIS	1.2425	0.1035	12	1.0845
KEGG_MEDICUS_VARIANT_AMPLIFIED_MYC_TO_CELL_CYCLE_G1_S	1.241	0.1128	11	1.0771
HALLMARK_APICAL_SURFACE	4.3244	0.1006	43	1.0744
REACTOME_REGULATION_OF_ENDOGENOUS_RETROELEMENTS_BY_KRAB_ZFP_PROTEINS	4.3195	0.0982	44	1.0663
REACTOME_PYRIMIDINE_CATABOLISM	1.2387	0.1126	11	1.0662
REACTOME_INACTIVATION_OF_CSF3_G_CSF_SIGNALING	2.4017	0.1044	23	1.0656
REACTOME_FORMATION_OF_THE_POLYBROMO_BAF_PBAF_COMPLEX	1.9188	0.1066	18	1.0646
KEGG_MEDICUS_REFERENCE_TSH_TG_SIGNALING_PATHWAY	3.1578	0.1089	29	1.0643
KEGG_MEDICUS_REFERENCE_GF_RTK_PI3K_SIGNALING_PATHWAY	7.1521	0.0917	78	1.0627
HALLMARK_IL2_STAT5_SIGNALING	17.1373	0.0897	191	1.06
REACTOME_REGULATION_OF_PYRUVATE_METABOLISM	3.1547	0.1088	29	1.0571
REACTOME_ABORTIVE_ELONGATION_OF_HIV_1_TRANSCRIPT_IN_THE_ABSENCE_OF_TAT	2.3971	0.1262	19	1.0529
KEGG_MEDICUS_REFERENCE_ACTH_CORTISOL_SIGNALING_PATHWAY	3.1523	0.1017	31	1.0514
REACTOME_YAP1_AND_WWTR1_TAZ_STIMULATED_GENE_EXPRESSION	1.5243	0.1173	13	1.0486
KEGG_MEDICUS_REFERENCE_TIGHT_JUNCTION_ACTIN_SIGNALING_PATHWAY	3.151	0.0985	32	1.0482
REACTOME_MITOCHONDRIAL_TRNA_AMINOACYLATION	1.9118	0.1125	17	1.0422
REACTOME_REGULATION_OF_GLYCOLYSIS_BY_FRUCTOSE_2_6_BISPHOSPHATE_METABOLISM	1.5226	0.1269	12	1.0418
KEGG_MEDICUS_VARIANT_LRP6_OVEREXPRESSION_TO_WNT_SIGNALING_PATHWAY	4.3037	0.105	41	1.0403
REACTOME_REGULATION_OF_INSULIN_SECRETION	7.1248	0.0937	76	1.04

REACTOME_SIGNALING_BY_SCF_KIT	4.3013	0.1	43	1.0364
KEGG_MEDICUS_REFERENCE_SKELETAL_TYPE_VGCC_RYR_SIGNALING	1.9092	0.1005	19	1.0339
REACTOME_INOSITOL_PHOSPHATE_METABOLISM	4.2983	0.0915	47	1.0316
HALLMARK_COMPLEMENT	17.0134	0.0895	190	1.0295
KEGG_MEDICUS_REFERENCE_DEPHOSPHORYLATION_OF_KINETOCHORE	1.231	0.1026	12	1.0288
KEGG_MEDICUS_REFERENCE_CXCR_GNB_G_PI3K_AKT_SIGNALING_PATHWAY	2.3862	0.0954	25	1.0228
KEGG_MEDICUS_VARIANT_MUTATION_CAUSED_ABERRANT_ABETA_TO_CROSSTALK_BETWEEN_EXTRINSIC_AND_INTRINSIC_APOPTOTIC_PATHWAYS	1.2295	0.1118	11	1.0213
KEGG_MEDICUS_REFERENCE_RAB7_REGULATED_MICROTUBULE_MINUS_END_DIRECTED_TRANSPORT	4.2886	0.1072	40	1.0157
REACTOME_FGFR3_LIGAND_BINDING_AND_ACTIVATION	1.5161	0.1166	13	1.0155
REACTOME_REGULATION_OF_MITF_M_DEPENDENT_GENES_INVOLVED_IN_PIGMENTATION	4.2876	0.1021	42	1.0141
REACTOME_DISEASES_OF_DNA_REPAIR	4.2859	0.0932	46	1.0112
REACTOME_FORMATION_OF_THE_URETERIC_BUD	2.3805	0.1134	21	1.0068
REACTOME_INCRETIN_SYNTHESIS_SECRETION_AND_INACTIVATION	2.3799	0.1133	21	1.0051
KEGG_MEDICUS_VARIANT_OLIGOMERIC_CONFORMATION_PRPC_TO_ANTEROGRADE_AXONAL_TRANSPORT	1.2257	0.1021	12	1.0031
REACTOME_REGULATION_OF_RUNX3_EXPRESSION_AND_ACTIVITY	4.2808	0.0996	43	1.0028
KEGG_MEDICUS_VARIANT_MUTATION_CAUSED_ABERRANT_SNCA_TO_VGCC_CA2_APOPTOTIC_PATHWAY	1.2255	0.1021	12	1.002
REACTOME_ACYL_CHAIN_REMODELLING_OF_PS	1.8986	0.1055	18	0.9999
REACTOME_DISEASES_OF_HEMOSTASIS	1.8977	0.1116	17	0.997
KEGG_MEDICUS_REFERENCE_KISS1_KISS1R_PLCB_PKC_SIGNALING_PATHWAY	1.2243	0.1113	11	0.9964
REACTOME_FORMATION_OF_AXIAL_MESODERM	1.5108	0.1259	12	0.9944
REACTOME_INTERFERON_ALPHA_BETA_SIGNALING	7.0676	0.1104	64	0.9925
REACTOME_EGFR_DOWNREGULATION	3.1255	0.1042	30	0.9879
REACTOME_GAP_JUNCTION_ASSEMBLY	3.1253	0.1116	28	0.9873
REACTOME_TGF_BETA_RECEPTOR_SIGNALING_ACTIVATES_SMADS	4.2658	0.097	44	0.9783
REACTOME_GLUTATHIONE_CONJUGATION	3.1197	0.104	30	0.9742
REACTOME_BIOLOGICAL_OXIDATIONS	16.7762	0.0999	168	0.9709
REACTOME_HEDGEHOG_ON_STATE	7.0403	0.102	69	0.9698
REACTOME_UNFOLDED_PROTEIN_RESPONSE_UPR	7.031	0.0847	83	0.962
REACTOME_FGFR1_MUTANT_RECEPTOR_ACTIVATION	3.1135	0.1074	29	0.9594
KEGG_MEDICUS_REFERENCE_MDM2_P21_CELL_CYCLE_G1_S_N00536	1.2164	0.1106	11	0.9578
REACTOME_DDX58_IFIH1_MEDIATED_INDUCTION_OF_INTERFERON_ALPHA_BETA	7.0249	0.1098	64	0.957
REACTOME_THROMBIN_SIGNALLING_THROUGH_PROTEINASE_ACTIVATED_RECEPTORS_PARS	3.1091	0.0972	32	0.949
REACTOME_RNA_POLYMERASE_III_TRANSCRIPTION_TERMINATION	2.3587	0.1026	23	0.9467
REACTOME_MUSCLE_CONTRACTION	16.6709	0.0887	188	0.945
HALLMARK_HEME_METABOLISM	16.6615	0.0921	181	0.9426
REACTOME_FLT3_SIGNALING	4.2412	0.1116	38	0.938
HALLMARK_INFLAMMATORY_RESPONSE	16.6309	0.0849	196	0.9351
REACTOME_M_DECAY_DEGRADATION_OF_MATERNAL_MRNAS_BY_MATERNALLY_STORED_FACTORS	2.3521	0.1069	22	0.9284
REACTOME_DEFECTIVE_INTRINSIC_PATHWAY_FOR_APOPTOSIS	2.3506	0.0979	24	0.9243
KEGG_MEDICUS_VARIANT_MUTATION_CAUSED_ABERRANT_HTT_TO_TRANSPORT_OF_CALCIUM	1.8746	0.1041	18	0.9231
REACTOME_SLC_MEDIATED_TRANSPORT_OF_INORGANIC_ANIONS	4.230	0.120	35	0.921

	9	9		1
REACTOME_DNA_REPLICATION_PRE_INITIATION	6.9816	0.0812	86	0.921
KEGG_MEDICUS_PATHOGEN_HBV_HBX_TO_RAS_ERK_SIGNALING_PATHWAY	1.4918	0.0995	15	0.918
REACTOME_SIGNALING_BY_NTRK2_TRKB	2.3477	0.0978	24	0.9161
KEGG_MEDICUS_REFERENCE_MICROTUBULE_NUCLEATION	2.347	0.0939	25	0.9143
REACTOME_IRE1ALPHA_ACTIVATES_CHAPERONES	4.2249	0.096	44	0.9112
REACTOME_INTRA_GOLGI_AND_RETROGRADE_GOLGI_TO_ER_TRAFFIC	16.5073	0.0873	189	0.9046
REACTOME_RHOG_GTPASE_CYCLE	6.9591	0.0967	72	0.9023
HALLMARK_REACTIVE_OXYGEN_SPECIES_PATHWAY	4.2152	0.0937	45	0.8953
REACTOME_PHASE_II_CONJUGATION_OF_COMPOUNDS	6.9438	0.0951	73	0.8895
REACTOME_PROGRAMMED_CELL_DEATH	16.4174	0.0902	182	0.8824
REACTOME_MRNA_SPLICING	16.4153	0.0855	192	0.8819
REACTOME_DEGRADATION_OF_GLI1_BY_THE_PROTEASOME	4.2066	0.0935	45	0.8812
REACTOME_NOREPINEPHRINE_NEUROTRANSMITTER_RELEASE_CYCLE	1.8611	0.1095	17	0.88
REACTOME_REGULATION_OF_HSF1_MEDIATED_HEAT_SHOCK_RESPONSE	6.9251	0.0949	73	0.874
KEGG_MEDICUS_REFERENCE_RAD51_DSDNA_DESTABILIZATION	1.1986	0.109	11	0.8718
REACTOME_CREB1_PHOSPHORYLATION_THROUGH_THE_ACTIVATION_OF_ADENYLATE_CYCLASE	1.4802	0.1233	12	0.8711
REACTOME_SIGNALING_BY_ROBO_RECEPTORS	16.3205	0.0907	180	0.8585
REACTOME_RUNX2_REGULATES_BONE_DEVELOPMENT	3.0707	0.1059	29	0.858
REACTOME_CILIUM_ASSEMBLY	16.2961	0.0953	171	0.8525
REACTOME_EXTRA_NUCLEAR_ESTROGEN_SIGNALING	6.8969	0.0932	74	0.8506
KEGG_MEDICUS_REFERENCE_DNA_END_RESECTION_AND_RPA_LOADING	1.4751	0.0983	15	0.8505
REACTOME_CELL_DEATH_SIGNALLING_VIA_NRAGE_NRIF_AND_NADE	6.8958	0.0945	73	0.8497
REACTOME_RET_SIGNALING	4.1873	0.1074	39	0.8496
KEGG_MEDICUS_VARIANT_MUTATION_ACTIVATED_EGFR_TO_PLCG_ERK_SIGNALING_PATHWAY	1.4748	0.1053	14	0.8494
KEGG_MEDICUS_REFERENCE_BMP_HAMP_SIGNALING_PATHWAY	1.1936	0.1085	11	0.8478
REACTOME_MITOTIC_G2_G2_M_PHASES	16.2628	0.0914	178	0.8443
HALLMARK_HYPOXIA	16.2508	0.0869	187	0.8414
REACTOME_MODULATION_OF_HOST_RESPONSES_BY_IFN_STIMULATED_GENES	1.8487	0.1087	17	0.8402
REACTOME_RIBAVIRIN_ADME	1.192	0.1192	10	0.8397
REACTOME_CLEC7A_DECTIN_1_INDUCES_NFAT_ACTIVATION	1.1917	0.1083	11	0.8384
REACTOME_DISEASES_ASSOCIATED_WITH_O_GLYCOSYLATION_OF_PROTEINS	6.8799	0.1166	59	0.8365
HALLMARK_EPITHELIAL_MESENCHYMAL_TRANSITION	16.2205	0.084	193	0.8339
REACTOME_FRS_MEDIATED_FGFR1_SIGNALING	2.3178	0.1054	22	0.8335
REACTOME_APOPTOTIC_CLEAVAGE_OF_CELL_ADHESION_PROTEINS	1.1894	0.1081	11	0.8274
REACTOME_CARGO_TRAFFICKING_TO_THE_PERICILIARY_MEMBRANE	6.8689	0.1108	62	0.8273
REACTOME_MATURATION_OF_SARS_COV_2_SPIKE_PROTEIN	4.1718	0.107	39	0.8242
REACTOME_TRAFFICKING_OF_AMPA_RECEPTORS	3.0564	0.1054	29	0.8241
REACTOME_GLUCOSE_METABOLISM	6.862	0.0915	75	0.8216
REACTOME_DISEASES_OF_CARBOHYDRATE_METABOLISM	3.0541	0.1018	30	0.8187
REACTOME_REGULATION_OF_IFNG_SIGNALING	1.4669	0.1048	14	0.8175

REACTOME_FREE_FATTY_ACIDS_REGULATE_INSULIN_SECRETION	1.1873	0.1079	11	0.8169
REACTOME_GLYCOSPHINGOLIPID_CATABOLISM	3.0525	0.0954	32	0.8149
REACTOME_VOLTAGE_GATED_POTASSIUM_CHANNELS	4.1638	0.1016	41	0.8111
REACTOME_MAP2K_AND_MAPK_ACTIVATION	4.1578	0.1039	40	0.8012
REACTOME_DIGESTION_AND_ABSORPTION	1.8355	0.108	17	0.7982
REACTOME_SIGNALING_BY_NOTCH2	3.0443	0.0951	32	0.7954
REACTOME_INTERLEUKIN_12_SIGNALING	4.1526	0.1013	41	0.7926
REACTOME_DISSOLUTION_OF_FIBRIN_CLOT	1.4595	0.1123	13	0.7879
REACTOME_SIGNALING_BY_ALK	3.0403	0.1086	28	0.7858
REACTOME_DEFECTS_OF_CONTACT_ACTIVATION_SYSTEM_CAS_AND_KALLIKREIN_KININ_SYSTEM_KKS	1.4581	0.1041	14	0.7822
REACTOME_SIGNALING_BY_FGFR2_IIIA_TM	2.2988	0.121	19	0.781
REACTOME_RESPONSE_OF_MTB_TO_PHAGOCYTOSIS	2.2988	0.0999	23	0.7809
REACTOME_DEGRADATION_OF_CRY_AND_PER_PROTEINS	4.1433	0.0921	45	0.7774
KEGG_MEDICUS_VARIANT_MUTATION_ACTIVATED_CACNA1D_H_TO_ANGIOTENSIN_ALDOSTERONE_SIGNALING_PATHWAY	2.2951	0.1043	22	0.7709
KEGG_MEDICUS_REFERENCE_CRHR_PKA_ACTH_SIGNALING_PATHWAY	2.2949	0.0918	25	0.7701
REACTOME_INTERLEUKIN_10_SIGNALING	4.1383	0.0962	43	0.7692
KEGG_MEDICUS_REFERENCE_EGF_EGFR_PLCG_CALCINEURIN_SIGNALING_PATHWAY	1.454	0.1039	14	0.7659
REACTOME_TAK1_DEPENDENT_IKK_AND_NF_KAPPA_B_ACTIVATION	4.1362	0.0985	42	0.7658
KEGG_MEDICUS_REFERENCE_EP_NE_ADRB_CAMP_SIGNALING_PATHWAY	1.4537	0.0969	15	0.7646
HALLMARK_APICAL_JUNCTION	15.9255	0.0856	186	0.7611
REACTOME_APOPTOTIC_CLEAVAGE_OF_CELLULAR_PROTEINS	4.1326	0.1148	36	0.7598
KEGG_MEDICUS_REFERENCE_EREG_EGFR_RAS_ERK_SIGNALING_PATHWAY	1.4514	0.0968	15	0.7555
REACTOME_RECYCLING_OF_BILE_ACIDS_AND_SALTS	1.8219	0.1215	15	0.7547
REACTOME_PRC2_METHYLATES_HISTONES_AND_DNA	1.4511	0.1036	14	0.754
REACTOME_NEPHRIN_FAMILY_INTERACTIONS	2.2888	0.104	22	0.7534
REACTOME_FCERI_MEDIATED_CA_2_MOBILIZATION	3.0243	0.0945	32	0.7479
REACTOME_REGULATION_OF_ENDOGENOUS_RETROELEMENTS	6.7699	0.0903	75	0.745
REACTOME_RHO_GTPASES_ACTIVATE_WASPS_AND_WAVES	4.1234	0.1145	36	0.7448
KEGG_MEDICUS_ENV_FACTOR_E2_TO_RAS_ERK_SIGNALING_PATHWAY	1.8186	0.1137	16	0.7442
REACTOME_CA_DEPENDENT_EVENTS	4.1217	0.1145	36	0.7421
REACTOME_DENGUE_VIRUS_HOST_INTERACTIONS	15.8422	0.0744	213	0.7406
KEGG_MEDICUS_PATHOGEN_HBV_LHBS_TO_PKC_ERK_SIGNALING_PATHWAY	1.1714	0.0976	12	0.74
KEGG_MEDICUS_REFERENCE_WNT_SIGNALING_PATHWAY	4.1188	0.0981	42	0.7373
HALLMARK_MTORC1_SIGNALING	15.8218	0.0842	188	0.7355
REACTOME_HDMS_DEMETHYLATE_HISTONES	2.2814	0.1086	21	0.733
REACTOME_INTERACTION_BETWEEN_L1_AND_ANKYRINS	3.0178	0.1006	30	0.7325
REACTOME_SYNTHESIS_OF_BILE_ACIDS_AND_BILE_SALTS_VIA_7ALPHA_HYDROXYCHOLESTEROL	2.2811	0.1037	22	0.732
REACTOME_PCP_CE_PATHWAY	6.7473	0.0888	76	0.7263
REACTOME_THROMBOXANE_SIGNALLING_THROUGH_TP_RECEPTOR	2.2788	0.095	24	0.7257
REACTOME_SYNTHESIS_OF_BILE_ACIDS_AND_BILE_SALTS	3.0144	0.0942	32	0.7244
REACTOME_RNA_POLYMERASE_III_CHAIN_ELONGATION	1.811	0.100	18	0.722

	8	7		1
KEGG_MEDICUS_REFERENCE_IL10_FAMILY_TO_JAK_STAT_SIGNALING_PATHWAY	1.442 6	0.111	13	0.72
HALLMARK_TNFA_SIGNALING_VIA_NFKB	15.74 21	0.082 4	191	0.715 9
KEGG_MEDICUS_REFERENCE_DOUBLE_STRAND_BREAK_SIGNALING	1.166 1	0.106	11	0.714 3
REACTOME_CRISTAE_FORMATION	1.165 6	0.106	11	0.711 9
REACTOME_SIGNALING_BY_FGFR2	6.727 5	0.094 8	71	0.709 8
HALLMARK_GLYCOLYSIS	15.71 57	0.082 7	190	0.709 4
REACTOME_GAB1_SIGNALOSOME	1.807 7	0.113	16	0.709 1
HALLMARK_ALLOGRAFT_REJECTION	15.70 21	0.082 2	191	0.706
REACTOME_GLYCOSAMINOGLYCAN_PROTEIN_LINKAGE_REGION_BIOSYNTHESIS	3.005 4	0.107 3	28	0.703 2
REACTOME_FGFRL1_MODULATION_OF_FGFR1_SIGNALING	1.438 4	0.110 6	13	0.702 9
REACTOME_SYNTHESIS_OF_LEUKOTRIENES_LT_AND_EOXINS_EX	2.269 1	0.119 4	19	0.698 8
REACTOME_KIDNEY_DEVELOPMENT	4.093	0.091	45	0.694 9
KEGG_MEDICUS_REFERENCE_TAC3_TACR3_PLC_PKC_SIGNALING_PATHWAY	1.161 6	0.116 2	10	0.692 8
REACTOME_CHROMATIN_ORGANIZATION	15.61 57	0.081 3	192	0.684 7
REACTOME_NUCLEOTIDE_CATABOLISM	2.996	0.099 9	30	0.681
KEGG_MEDICUS_REFERENCE_EGF_EGFR_RAS_JNK_SIGNALING_PATHWAY	1.432 9	0.110 2	13	0.680 8
REACTOME_GAP_JUNCTION_TRAFFICKING_AND_REGULATION	4.082 6	0.099 6	41	0.677 8
REACTOME_CREATINE_METABOLISM	1.158 1	0.115 8	10	0.675 8
REACTOME_RORA_B_C_AND_NR1D1_REV_ERBA_REGULATE_GENE_EXPRESSION	2.260 3	0.094 2	24	0.674 6
KEGG_MEDICUS_VARIANT_SCRAPIE_CONFORMATION_PRPSC_TO_TRANSPORT_OF_CALCIUM	1.796 6	0.105 7	17	0.673 8
KEGG_MEDICUS_VARIANT_MUTATION_CAUSED_ABERRANT_HTT_TO_ANTEROGRADE_AXONAL_TRANSPORT	2.258 9	0.098 2	23	0.670 8
REACTOME_RNA_POLYMERASE_I_TRANSCRIPTION_INITIATION	4.078	0.094 8	43	0.670 4
KEGG_MEDICUS_REFERENCE_METHIONINE_DEGRADATION	1.156 6	0.115 7	10	0.668 3
KEGG_MEDICUS_VARIANT_MUTATION_INACTIVATED_APC_TO_WNT_SIGNALING_PATHWAY	1.156 4	0.096 4	12	0.667 4
REACTOME_VASOPRESSIN_REGULATES_RENAL_WATER_HOMEOSTASIS_VIA_AQUAPORINS	4.075 3	0.097	42	0.665 9
REACTOME_SLC_MEDIATED_TRANSPORT_OF_ORGANIC_CATIONS	1.156	0.105 1	11	0.665 6
REACTOME_THE_CRY_PER_KINASE_COMPLEX_REPRESSES_TRANSACTIVATION_BY_THE_BMAL_CLOCK_ARNTL_C LOCK_COMPLEX	1.156	0.105 1	11	0.665 4
KEGG_MEDICUS_PATHOGEN_HPV_E6_TO_NOTCH_SIGNALING_PATHWAY_N00382	1.429	0.109 9	13	0.665 2
REACTOME_COMPLEMENT_CASCADE	4.073 5	0.086 7	47	0.663
REACTOME_CAMK_IV_MEDIATED_PHOSPHORYLATION_OF_CREB	1.155 2	0.115 5	10	0.661 5
REACTOME_REGULATION_OF_KIT_SIGNALING	1.792 7	0.112	16	0.661 4
REACTOME_LDL_CLEARANCE	2.251 5	0.118 5	19	0.650 2
KEGG_MEDICUS_ENV_FACTOR_NNK_NNN_TO_PI3K_SIGNALING_PATHWAY_N01350	1.152 6	0.096 1	12	0.649 2
REACTOME_FCERI_MEDIATED_MAPK_ACTIVATION	2.979 7	0.090 3	33	0.642 3
REACTOME_TP53_REGULATES_TRANSCRIPTION_OF_CELL_DEATH_GENES	4.059 5	0.099	41	0.64
REACTOME_EXPORT_OF_VIRAL_RIBONUCLEOPROTEINS_FROM_NUCLEUS	2.978 3	0.106 4	28	0.638 9
REACTOME_DEVELOPMENTAL_LINEAGE_OF_PANCREATIC_DUCTAL_CELLS	4.057 9	0.088 2	46	0.637 4
REACTOME_RETINOID_CYCLE_DISEASE_EVENTS	1.150 1	0.104 6	11	0.636 9
REACTOME_FORMATION_OF_WDR5_CONTAINING_HISTONE_MODIFYING_COMPLEXES	2.977 4	0.090 2	33	0.636 8
REACTOME_INTERLEUKIN_21_SIGNALING	1.149 9	0.115	10	0.636 1

REACTOME_FERTILIZATION	1.7841	0.0991	18	0.6336
REACTOME_DECTIN_1_MEDIATED_NONCANONICAL_NF_KB_SIGNALING	4.0539	0.0863	47	0.6309
REACTOME_TRANSCRIPTIONAL_REGULATION_OF_GRANULOPOIESIS	2.9722	0.1061	28	0.6244
REACTOME_NEGATIVE_REGULATION_OF_MAPK_PATHWAY	4.0498	0.0964	42	0.6241
KEGG_MEDICUS_REFERENCE_BLOCKING_UBIQUITINATION_OF_H2AK119_BY_CK2	1.1474	0.0956	12	0.6236
REACTOME_REGULATION_OF_PD_L1_CD274_TRANSCRIPTION	4.0493	0.1012	40	0.6233
REACTOME_SIGNALING_BY_BMP	2.9713	0.1061	28	0.6224
REACTOME_PRE_NOTCH_EXPRESSION_AND_PROCESSING	4.0482	0.09	45	0.6215
REACTOME_DOWNREGULATION_OF_ERBB2_SIGNALING	2.9709	0.1061	28	0.6214
REACTOME_RESPONSE_OF_EIF2AK4_GCN2_TO_AMINO_ACID_DEFICIENCY	6.6207	0.0798	83	0.6211
REACTOME_TRANSCRIPTIONAL_REGULATION_OF_BROWN_AND_BEIGE_ADIPOCYTE_DIFFERENTIATION	2.9705	0.099	30	0.6204
REACTOME_SYNTHESIS_OF_VERY_LONG_CHAIN_FATTY_ACYL_COAS	1.7797	0.1047	17	0.6197
REACTOME_POLYMERASE_SWITCHING_ON_THE_C_STRAND_OF_THE_TELOMERE	2.2388	0.0933	24	0.615
KEGG_MEDICUS_VARIANT_SCRAPIE_CONFORMATION_PRPSC_TO_MGLUR5_CA2_APOPTOTIC_PATHWAY	1.778	0.0988	18	0.6141
REACTOME_INTERLEUKIN_6_FAMILY_SIGNALING	2.237	0.0973	23	0.6102
KEGG_MEDICUS_REFERENCE_CXCL12_CXCR4_PKC_ERK_SIGNALING_PATHAWAY	1.7759	0.0935	19	0.6073
REACTOME_SEMA4D_IN_SEMAPHORIN_SIGNALING	2.2355	0.0931	24	0.6059
HALLMARK_ESTROGEN_RESPONSE_EARLY	15.2826	0.0809	189	0.6025
KEGG_MEDICUS_REFERENCE_TETHERING_OF_LATE_ENDOSOMES_AND_LYSOSOMES	1.1425	0.1143	10	0.6003
KEGG_MEDICUS_REFERENCE_BILE_ACID_BIOSYNTHESIS	1.4111	0.1008	14	0.5934
REACTOME_REGULATED_PROTEOLYSIS_OF_P75NTR	1.1398	0.1036	11	0.5872
KEGG_MEDICUS_REFERENCE_PDGF_PDGFR_PLCG_CAMK_SIGNALING_PATHWAY	1.7682	0.1105	16	0.583
REACTOME_MEIOTIC_RECOMBINATION	2.2268	0.0968	23	0.5819
REACTOME_GABA_B_RECEPTOR_ACTIVATION	4.0223	0.0958	42	0.579
REACTOME_RUNX3_REGULATES_NOTCH_SIGNALING	1.4072	0.1005	14	0.5777
REACTOME_PRESYNAPTIC_FUNCTION_OF_KAINATE_RECEPTORS	2.2249	0.1059	21	0.5768
REACTOME_IONOTROPIC_ACTIVITY_OF_KAINATE_RECEPTORS	1.1375	0.1138	10	0.5759
REACTOME_ACTIVATION_OF_BAD_AND_TRANSLOCATION_TO_MITOCHONDRIA	1.7659	0.1177	15	0.5755
REACTOME_PLATELET_CALCIUM_HOMEOSTASIS	2.9514	0.1054	28	0.5751
REACTOME_TRANSCRIPTION_OF_THE_HIV_GENOME	6.5645	0.1076	61	0.5744
HALLMARK_OXIDATIVE_PHOSPHORYLATION	15.1412	0.0851	178	0.5677
REACTOME_CONVERSION_FROM_APC_C_CDC20_TO_APC_C_CDH1_IN_LATE_ANAPHASE	1.7629	0.0979	18	0.566
REACTOME_SIGNALING_BY_CTNNB1_PHOSPHO_SITE_MUTANTS	1.4042	0.1003	14	0.5654
KEGG_MEDICUS_REFERENCE_ANTEROGRADE_AXONAL_TRANSPORT	2.2196	0.1057	21	0.5621
REACTOME_PKA_ACTIVATION_IN_GLUCAGON_SIGNALLING	1.7614	0.1036	17	0.5612
REACTOME_ERK_MAPK_TARGETS	2.2191	0.1009	22	0.5607
KEGG_MEDICUS_REFERENCE_NRG_ERBB4_PI3K_SIGNALING_PATHWAY	1.1343	0.1031	11	0.5604
REACTOME_TBC_RABGAPS	4.009	0.0955	42	0.5572
KEGG_MEDICUS_REFERENCE_WNT_SIGNALING_MODULATION_SOST_LRP4	1.402	0.1078	13	0.5568
REACTOME_THE_PHOTOTRANSDUCTION_CASCADE	2.9434	0.0981	30	0.5562
REACTOME_NETRIN_1_SIGNALING	4.007	0.093	43	0.555

	8	2		3
KEGG_MEDICUS_REFERENCE_N_GLYCAN_PRECURSOR_BIOSYNTHESIS_ALG6_TO_OST	1.1331	0.103	11	0.5546
REACTOME_MISCELLANEOUS_TRANSPORT_AND_BINDING_EVENTS	2.2158	0.0923	24	0.5516
REACTOME_REGULATION_OF_MITF_M_DEPENDENT_GENES_INVOLVED_IN_LYSOSOME_BIOGENESIS_AND_AUTOPHAGY	1.7574	0.1098	16	0.5484
REACTOME_METABOLISM_OF_ANGIOTENSINOGEN_TO_ANGIOTENSINS	1.7573	0.1098	16	0.5479
REACTOME_TP53_REGULATES_METABOLIC_GENES	6.5287	0.092	71	0.5446
REACTOME_NON_INTEGRIN_MEMBRANE_ECM_INTERACTIONS	6.5284	0.0848	77	0.5443
REACTOME_SEROTONIN_NEUROTRANSMITTER_RELEASE_CYCLE	1.7551	0.1032	17	0.5409
REACTOME_PHASE_0_RAPID_DEPOLARISATION	2.9368	0.0947	31	0.5406
KEGG_MEDICUS_VARIANT_MUTATION_ACTIVATED_PRKACA_TO_ACTH_CORTISOL_SIGNALING_PATHWAY	1.7549	0.1097	16	0.5403
REACTOME_GLYCOGEN_STORAGE_DISEASES	1.3977	0.1075	13	0.5392
REACTOME_RECRUITMENT_OF_MITOTIC_CENTROSOME_PROTEINS_AND_COMPLEXES	6.5217	0.0858	76	0.5388
REACTOME_SYNTHESIS_OF_PROSTAGLANDINS_PG_AND_THROMBOXANES_TX	1.3974	0.0998	14	0.5382
KEGG_MEDICUS_REFERENCE_MDA5_IRF7_3_SIGNALING_PATHWAY	1.1297	0.1027	11	0.5379
KEGG_MEDICUS_REFERENCE_PP2A_AKT_SIGNALING_PATHWAY	1.7539	0.0923	19	0.5371
REACTOME_TRAF6_MEDIATED_IRF7_ACTIVATION	1.7536	0.1096	16	0.5363
REACTOME_CASPASE_MEDIATED_CLEAVAGE_OF_CYTOSKELETAL_PROTEINS	1.1292	0.1027	11	0.5357
REACTOME_EARLY_SARS_COV_2_INFECTION_EVENTS	3.9933	0.1141	35	0.5316
REACTOME_ETHANOL_OXIDATION	1.1277	0.1025	11	0.5284
REACTOME_OTHER_INTERLEUKIN_SIGNALING	2.2069	0.1003	22	0.527
REACTOME_GENE_SILENCING_BY_RNA	3.99	0.095	42	0.526
KEGG_MEDICUS_PATHOGEN_KSHV_VGPCR_TO_GNB_G_PI3K_AKT_SIGNALING_PATHWAY	2.2062	0.0919	24	0.5249
REACTOME_RAC1_GTPASE_CYCLE	14.9629	0.085	176	0.5237
KEGG_MEDICUS_REFERENCE_P300_P21_CELL_CYCLE_G1_S	1.7496	0.1166	15	0.5235
REACTOME_CS_GAG_BIOSYNTHESIS	2.2054	0.105	21	0.5227
REACTOME_SIGNALING_BY_ACTIVIN	1.749	0.1029	17	0.5213
REACTOME_SIGNALING_BY_FGFR_IN_DISEASE	6.4986	0.1065	61	0.5196
REACTOME_SIGNALING_BY_ALK_IN_CANCER	6.4968	0.0764	85	0.5181
REACTOME_NOTCH4_ACTIVATION_AND_TRANSMISSION_OF_SIGNAL_TO_THE_NUCLEUS	1.1246	0.1022	11	0.5135
REACTOME_HEME_BIOSYNTHESIS	1.3912	0.0994	14	0.5134
REACTOME_ONCOGENE_INDUCED_SENESCENCE	2.924	0.0975	30	0.5102
REACTOME_FORMATION_OF_THE_DYSTROPHIN_GLYCOPROTEIN_COMPLEX_DGC	3.9789	0.1105	36	0.5079
REACTOME_NUCLEAR_RNA_DECAY	2.923	0.0943	31	0.5079
REACTOME_GABA_SYNTHESIS_RELEASE_REUPTAKE_AND_DEGRADATION	1.7446	0.109	16	0.5073
KEGG_MEDICUS_REFERENCE_CCR2_GNB_G_PI3K_NFKB_SIGNALING_PATHWAY	2.9206	0.0942	31	0.5022
REACTOME_NICOTINATE_METABOLISM	2.1972	0.0999	22	0.5
KEGG_MEDICUS_VARIANT_MUTATION_ACTIVATED_KCNJ5_TO_ANGIOTENSIN_ALDOSTERONE_SIGNALING_PATHWAY	2.1952	0.0954	23	0.4945
REACTOME_ACTIVATION_OF_KAINATE_RECEPTORS_UPON_GLUTAMATE_BINDING	2.9171	0.0972	30	0.4939
REACTOME_EUKARYOTIC_TRANSLATION_ELONGATION	6.4659	0.0862	75	0.4924
REACTOME_SIGNALING_BY_KIT_IN_DISEASE	2.194	0.1097	20	0.4912
REACTOME_REGULATED_NECROSIS	6.4644	0.1154	56	0.4911
REACTOME_HATS_ACETYLATE_HISTONES	6.463	0.095	68	0.490

	8	1		6
REACTOME_MATERNAL_TO_ZYGOTIC_TRANSITION_MZT	3.9665	0.0922	43	0.4876
REACTOME_ERYTHROPOIETIN_ACTIVATES_RAS	1.3848	0.0989	14	0.4876
REACTOME_DRUG_ADME	6.4596	0.0807	80	0.4872
KEGG_MEDICUS_REFERENCE_BCR_PLCG_CALCINEURIN_SIGNALING_PATHWAY	1.3847	0.0923	15	0.487
HALLMARK_WNT_BETA_CATENIN_SIGNALING	3.9633	0.0967	41	0.4823
REACTOME_ION_CHANNEL_TRANSPORT	14.7904	0.088	168	0.4811
REACTOME_ACETYLCHOLINE_REGULATES_INSULIN_SECRETION	1.1174	0.1117	10	0.4784
HALLMARK_MYOGENESIS	14.7471	0.0784	188	0.4704
REACTOME_GLUCAGON_TYPE_LIGAND_RECEPTORS	2.9054	0.0908	32	0.4662
REACTOME_GLUCONEOGENESIS	2.1846	0.0993	22	0.4653
KEGG_MEDICUS_REFERENCE_KINETOCHORE_MICROTUBULE_ATTACHMENT	1.7312	0.1018	17	0.4645
REACTOME_TRIGLYCERIDE_METABOLISM	2.9034	0.0907	32	0.4615
REACTOME_SIGNALING_BY_CYTOSOLIC_FGFR1_FUSION_MUTANTS	1.7288	0.1017	17	0.4569
KEGG_MEDICUS_PATHOGEN_ESCHERICHIA_ESPG_TO_MICROTUBULE_RHOA_SIGNALING_PATHWAY	1.3768	0.0983	14	0.4551
REACTOME_COLLAGEN_BIOSYNTHESIS_AND_MODIFYING_ENZYMES	6.4206	0.1053	61	0.4547
REACTOME_DOWNSTREAM_SIGNALING_OF_ACTIVATED_FGFR2	2.8994	0.1	29	0.4519
REACTOME_FOXO_MEDIATED_TRANSCRIPTION_OF_CELL_CYCLE_GENES	1.726	0.1015	17	0.4478
KEGG_MEDICUS_PATHOGEN_HCMV_UL33_TO_GNB_G_RHO_SIGNALING_PATHWAY	2.8968	0.0878	33	0.4457
REACTOME_RND1_GTPASE_CYCLE	3.9409	0.0985	40	0.4455
KEGG_MEDICUS_VARIANT_BCR_ABL_FUSION_KINASE_TO_RAS_ERK_SIGNALING_PATHWAY	1.3731	0.0981	14	0.4403
REACTOME_DNA_DOUBLE_STRAND_BREAK_RESPONSE	3.9368	0.0984	40	0.4389
KEGG_MEDICUS_VARIANT_LOSS_OF_VHL_TO_HIF_1_SIGNALING_PATHWAY	1.1086	0.0924	12	0.4357
REACTOME_RNA_POLYMERASE_III_TRANSCRIPTION	3.9342	0.096	41	0.4345
REACTOME_ABC_FAMILY_PROTEINS_MEDIATED_TRANSPORT	6.395	0.0752	85	0.4335
REACTOME_ESR_MEDIATED_SIGNALING	14.5964	0.0942	155	0.4333
KEGG_MEDICUS_VARIANT_MUTATION_ACTIVATED_GNAS_TO_ACTH_CORTISOL_SIGNALING_PATHWAY	2.8897	0.1032	28	0.429
REACTOME_REGULATION_OF_NF_KAPPA_B_SIGNALING	1.7199	0.0956	18	0.4284
REACTOME_FORMATION_OF_THE_EARLY_ELONGATION_COMPLEX	2.8882	0.1031	28	0.4253
REACTOME_TOLL_LIKE_RECEPTOR_CASCADES	14.5605	0.0916	159	0.4244
KEGG_MEDICUS_ENV_FACTOR_DCE_TO_DNA_ADDUCTS	1.369	0.1053	13	0.4241
KEGG_MEDICUS_REFERENCE_AUTOPHAGY_VESICLE_NUCLEATION_ELONGATION_MATURATION_LC3_II_FORMATION	1.1057	0.0921	12	0.4217
REACTOME_ACTIVATION_OF_THE_AP_1_FAMILY_OF_TRANSCRIPTION_FACTORS	1.1047	0.1105	10	0.4169
REACTOME_TRANSLESION_SYNTHESIS_BY_POLK	1.7155	0.1009	17	0.4143
REACTOME_ACETYLCHOLINE_NEUROTRANSMITTER_RELEASE_CYCLE	1.7148	0.1072	16	0.4121
REACTOME_IRAK4_DEFICIENCY_TLR2_4	1.7147	0.0953	18	0.4119
REACTOME_INSULIN_PROCESSING	2.1647	0.0984	22	0.4102
REACTOME_NF_KB_IS_ACTIVATED_AND_SIGNALS_SURVIVAL	1.3655	0.105	13	0.4098
REACTOME_ACTIVATION_OF_BH3_ONLY_PROTEINS	2.8797	0.0993	29	0.4053
REACTOME_SYNTHESIS_OF_BILE_ACIDS_AND_BILE_SALTS_VIA_24_HYDROXYCHOLESTEROL	1.3643	0.1137	12	0.4051
REACTOME_ADENYLATE_CYCLASE_INHIBITORY_PATHWAY	1.3639	0.0974	14	0.4036

REACTOME_SERINE_METABOLISM	1.1018	0.1102	10	0.4029
REACTOME_TRANSCRIPTIONAL_REGULATION_OF_PLURIPOTENT_STEM_CELLS	2.8769	0.1066	27	0.3987
REACTOME_SYNAPTIC_ADHESION_LIKE_MOLECULES	2.1601	0.1029	21	0.3976
REACTOME_ACYL_CHAIN_REMODELLING_OF_PE	2.1601	0.1029	21	0.3975
REACTOME_CYCLIN_A_CDK2_ASSOCIATED_EVENTS_AT_S_PHASE_ENTRY	6.3506	0.087	73	0.3966
REACTOME_PHOSPHORYLATION_OF_CD3_AND_TCR_ZETA_CHAINS	1.7095	0.095	18	0.395
REACTOME_REGULATION_OF_MRNA_STABILITY_BY_PROTEINS_THAT_BIND_AU_RICH_ELEMENTS	6.3473	0.0907	70	0.3938
REACTOME_PKA_MEDIATED_PHOSPHORYLATION_OF_CREB	2.1587	0.1079	20	0.3937
REACTOME_FATTY_ACID_METABOLISM	14.4273	0.0937	154	0.3916
REACTOME_TRANSCRIPTION_COUPLED_NUCLEOTIDE_EXCISION_REPAIR_TC_NER	6.337	0.0856	74	0.3853
REACTOME_RRNA_PROCESSING	14.3893	0.0841	171	0.3822
KEGG_MEDICUS_ENV_FACTOR_METALS_TO_RAS_ERK_SIGNALING_PATHWAY	1.0975	0.0915	12	0.3819
REACTOME_AEROBIC_RESPIRATION_AND_RESPIRATORY_ELECTRON_TRANSPORT	14.3828	0.0753	191	0.3806
REACTOME_THE_CANONICAL_RETINOID_CYCLE_IN_RODS_TWILIGHT_VISION	2.1538	0.1026	21	0.3802
REACTOME_SLC_MEDIATED_TRANSPORT_OF_AMINO_ACIDS	3.9007	0.1084	36	0.3797
REACTOME_SUMOYLATION_OF_DNA_DAMAGE_RESPONSE_AND_REPAIR_PROTEINS	6.3268	0.0904	70	0.3768
REACTOME_FCGR3A_MEDIATED_IL10_SYNTHESIS	3.8986	0.1054	37	0.3761
REACTOME_G_PROTEIN_BETA_GAMMA_SIGNALLING	2.8668	0.0896	32	0.3747
REACTOME_CELLULAR_RESPONSE_TO_CHEMICAL_STRESS	14.3588	0.0793	181	0.3747
REACTOME_INTERLEUKIN_27_SIGNALING	1.0959	0.0996	11	0.3744
REACTOME_COLLAGEN_DEGRADATION	6.3202	0.1036	61	0.3713
REACTOME_G0_AND_EARLY_G1	2.8651	0.1061	27	0.3706
REACTOME_ACTIVATION_OF_GENE_EXPRESSION_BY_SREBF_SREBP	3.8914	0.0998	39	0.3644
REACTOME_RNA_POLYMERASE_III_TRANSCRIPTION_INITIATION_FROM_TYPE_3_PROMOTER	2.8623	0.1022	28	0.364
REACTOME_ONCOGENIC_MAPK_SIGNALING	6.3113	0.0809	78	0.364
REACTOME_DIFFERENTIATION_OF_NAIVE_CD_T_CELLS_TO_T_HELPER_1_CELLS_TH1_CELLS	1.0936	0.0994	11	0.363
KEGG_MEDICUS_REFERENCE_CCR5_GNB_G_PLCB_G_PKC_SIGNALING_PATHWAY	2.861	0.11	26	0.361
REACTOME_REVERSIBLE_HYDRATION_OF_CARBON_DIOXIDE	1.0925	0.0993	11	0.3581
REACTOME_TFAP2_AP_2_FAMILY_REGULATES_TRANSCRIPTION_OF_GROWTH_FACTORS_AND_THEIR_RECEPTORS	1.0924	0.0993	11	0.3572
REACTOME_ANTIGEN_ACTIVATES_B_CELL_RECEPTOR_BCR_LEADING_TO_GENERATION_OF_SECOND_MESSENGERS	2.8578	0.0922	31	0.3533
REACTOME_SIGNALING_BY_INSULIN_RECEPTOR	6.298	0.0818	77	0.3529
REACTOME_TELOMERE_MAINTENANCE	6.2951	0.1015	62	0.3505
REACTOME_NEGATIVE_REGULATION_OF_NOTCH4_SIGNALING	3.8829	0.0924	42	0.3504
KEGG_MEDICUS_PATHOGEN_HIV_GP120_TO_CXCR4_GNAQ_PLCB_G_CALCINEURIN	1.3507	0.09	15	0.3504
REACTOME_KERATAN_SULFATE_BIOSYNTHESIS	2.8565	0.102	28	0.3502
REACTOME_WNT_LIGAND_BIOGENESIS_AND_TRAFFICKING	2.1428	0.0932	23	0.3498
KEGG_MEDICUS_PATHOGEN_HCMV_US28_TO_GNAQ_PLCB_G_CALCINEURIN_SIGNALING_PATHWAY	1.695	0.1059	16	0.3486
REACTOME_TRANSLOCATION_OF_SLC2A4_GLUT4_TO_THE_PLASMA_MEMBRANE	6.2921	0.0968	65	0.3479
REACTOME_NEUREXINS_AND_NEUROLIGINS	6.291	0.121	52	0.347
REACTOME_INTEGRIN_CELL_SURFACE_INTERACTIONS	6.2888	0.0767	82	0.3452
REACTOME_DEGRADATION_OF_BETA_CATENIN_BY_THE_DESTRUCTION_COMPLEX	6.2887	0.0925	68	0.3451
REACTOME_INTRAFLAGELLAR_TRANSPORT	3.879	0.097	40	0.344

	4			7
REACTOME_METABOLISM_OF_VITAMINS_AND_COFACTORS	14.23<br>27	0.082<br>3	173	0.343<br>6
HALLMARK_KRAS_SIGNALING_UP	14.22<br>58	0.074<br>9	190	0.341<br>9
REACTOME_AKT_PHOSPHORYLATES_TARGETS_IN_THE_CYTOSOL	1.348	0.096<br>3	14	0.339<br>5
REACTOME_MITOTIC_PROMETAPHASE	14.21<br>47	0.077<br>3	184	0.339<br>1
REACTOME_PYRUVATE_METABOLISM	3.875<br>6	0.092<br>3	42	0.338<br>5
REACTOME_MITOTIC_METAPHASE_AND_ANAPHASE	14.20<br>44	0.070<br>7	201	0.336<br>6
REACTOME_BMAL1_CLOCK_NPAS2_ACTIVATES_CIRCADIAN_EXPRESSION	2.137<br>9	0.101<br>8	21	0.336<br>3
REACTOME_SEMA3A_PAK_DEPENDENT_AXON_REPULSION	1.690<br>9	0.105<br>7	16	0.335<br>6
REACTOME_TRANSPORT_OF_MATURE_MRNAS_DERIVED_FROM_INTRONLESS_TRANSCRIPTS	3.873<br>3	0.104<br>7	37	0.334<br>8
REACTOME_PROTEIN_METHYLATION	1.087<br>2	0.108<br>7	10	0.332<br>1
REACTOME_PHASE_4_RESTING_MEMBRANE_POTENTIAL	1.689<br>4	0.112<br>6	15	0.330<br>8
REACTOME_RECOGNITION_OF_DNA_DAMAGE_BY_PCNA_CONTAINING_REPLICATION_COMPLEX	2.847<br>7	0.094<br>9	30	0.329<br>5
KEGG_MEDICUS_REFERENCE_IL12_23_TO_JAK_STAT_SIGNALING_PATHWAY	1.085<br>8	0.108<br>6	10	0.325<br>3
REACTOME_SIGNALING_BY_FGFR2_IN_DISEASE	3.866<br>7	0.092<br>1	42	0.323<br>8
REACTOME_NEGATIVE_REGULATION_OF_FGFR4_SIGNALING	2.844<br>4	0.094<br>8	30	0.321<br>7
REACTOME_REGULATION_OF_ENDOGENOUS_RETROELEMENTS_BY_PIWI_INTERACTING_RNAS_PIRNAS	3.865<br>3	0.107<br>4	36	0.321<br>7
REACTOME_SLC_MEDIATED_TRANSPORT_OF_ORGANIC_ANIONS	3.865<br>2	0.117<br>1	33	0.321<br>4
REACTOME_SARS_COV_1_HOST_INTERACTIONS	6.26	0.075<br>4	83	0.321<br>3
KEGG_MEDICUS_REFERENCE_FGF_FGFR_PI3K_SIGNALING_PATHWAY	2.842<br>9	0.094<br>8	30	0.318
REACTOME_ACYL_CHAIN_REMODELLING_OF_PI	1.342<br>1	0.095<br>9	14	0.315<br>9
HALLMARK_XENOBIOTIC_METABOLISM	14.11<br>97	0.075<br>1	188	0.315<br>7
REACTOME_GLYCOGEN_BREAKDOWN_GLYCOGENOLYSIS	1.684<br>2	0.112<br>3	15	0.314<br>2
REACTOME_ADHERENS_JUNCTIONS_INTERACTIONS	14.10<br>61	0.088<br>7	159	0.312<br>3
REACTOME_SYNTHESIS_OF_SUBSTRATES_IN_N_GLYCAN_BIOSYTHESIS	6.249	0.109<br>6	57	0.312<br>2
REACTOME_ANTIGEN_PROCESSING_UB_ATP_INDEPENDENT_PROTEASOMAL_DEGRADATION	1.682<br>8	0.099	17	0.309<br>8
REACTOME_REGULATION_OF_BETA_CELL_DEVELOPMENT	3.854<br>1	0.101<br>4	38	0.303<br>2
REACTOME_SWITCHING_OF_ORIGINS_TO_A_POST_REPLICATIVE_STATE	6.232	0.083<br>1	75	0.298
REACTOME_INFECTION_WITH_MYCOBACTERIUM_TUBERCULOSIS	2.834	0.105	27	0.297<br>1
REACTOME_GPER1_SIGNALING	3.849<br>9	0.087<br>5	44	0.296<br>3
REACTOME_ERYTHROCYTES_TAKE_UP_CARBON_DIOXIDE_AND_RELEASE_OXYGEN	1.337	0.111<br>4	12	0.295<br>2
REACTOME_NUCLEAR_IMPORT_OF_REV_PROTEIN	2.831<br>2	0.094<br>4	30	0.290<br>4
REACTOME_MITOCHONDRIAL_PROTEIN_DEGRADATION	6.218<br>7	0.078<br>7	79	0.287
KEGG_MEDICUS_REFERENCE_EGF_EGFR_PLCG_ERK_SIGNALING_PATHWAY	1.334<br>9	0.089	15	0.286<br>8
REACTOME_REGULATION_OF_GENE_EXPRESSION_BY_HYPOXIA_INDUCIBLE_FACTOR				
